# In vitro α-glucosidase inhibition, molecular dynamics and docking study of phenyl carbamoyl methoxy thiosemicarbazone derivatives as potential anti-diabetic agents

**DOI:** 10.1080/14756366.2025.2580515

**Published:** 2025-11-04

**Authors:** Shirin Valadbeigi, Reza Saghiri, Zahra Kianmehr, Roghieh Mirzazadeh, Shohreh Khatami

**Affiliations:** ^a^ Department of Biochemistry, NT.C., Islamic Azad University, Tehran, Iran; ^b^ Department of Biochemistry, Pasteur Institute of Iran, Tehran, Iran

**Keywords:** α-glucosidase, molecular dynamic, docking study, thiosemicarbazone

## Abstract

Alpha-glucosidase inhibitors have been considered as the most effective agents in preventing hyperglycaemia and alternative targets for the treatment of Diabetes mellitus (DM). This study aimed to synthesise novel phenyl carbamoyl methoxy thiosemicarbazone derivatives and evaluate their potential as α-glucosidase inhibitors through biochemical assays, cytotoxicity screening, molecular docking, and molecular dynamics simulations. The tested derivatives exhibited a range of inhibitory potential, from moderate to strong as compared to acarbose. Derivative 7e revealed the least IC50 value among the tested compounds. 7e in the kinetic assay acted as a competitive inhibitor of the α-glucosidase. The cytotoxic effect 7e was assessed against the A549 and MDA-MB-453 cell lines. MD simulation revealed that **7e** could affect the stability, flexibility, thermodynamics, and structure of α-glucosidase enzymes such as acarbose. Compound 7e demonstrates strong α-glucosidase inhibitory activity with low cytotoxicity in both cell lines, underscoring its potential as a lead candidate for antidiabetic drug development.

## Introduction

Diabetes mellitus (DM) is a chronic metabolic disorder characterised by dysfunctional insulin secretion, insulin resistance, or a combination of both, resulting in dysregulated blood glucose levels^1–4^. The worldwide incidence of DM is on the rise, with projections indicating that over 642 million individuals will be affected by the condition by 2040^1^.

Chronic hyperglycaemia can lead to severe complications, such as obesity, blindness, nerve damage, cardiovascular disease, and kidney failure^5^^,^^6^. Several pharmacological interventions have been used to control hyperglycaemia, including insulin therapy for type 1 diabetes mellitus (T1DM) and non-insulin medications for type 2 diabetes mellitus (T2DM).

Non-insulin pharmaceuticals regulate blood glucose by inhibiting gastrointestinal glucose absorption and hepatic gluconeogenesis, while simultaneously augmenting renal glucose reabsorption^5^^,^^7^. Inhibition of enzymes responsible for the hydrolysis of complex carbohydrates, particularly α-glucosidase, represents a significant strategy for attenuating glucose absorption within the gastrointestinal tract^8^^,^^9^. The hydrolase, α-glucosidase, located in the small intestine’s brush border, facilitates the hydrolysis of complex carbohydrates, yielding glucose and other monosaccharides^10–14^.

α-glucosidase inhibitors that inhibit the digestion of carbohydrates and glucose absorption can effectively control hyperglycaemia^15^. In recent years, common α-glucosidase inhibitors such as acarbose, voglibose, and miglitol have been used in clinics^11^^,^^15^^,^^16^. However, these drugs have adverse effects, including abdominal pain, nausea, diarrhoea, and flatulence^17^^,^^18^. Therefore, much research has focused on developing novel, safe, and efficient α-glucosidase inhibitors^2^^,^^11^.

Thiosemicarbazones have the general formula R1R2C = N–NH (C = S) NHR, and several of their derivatives are found in synthetic and natural products. They exhibit remarkable biological activities, including antituberculosis, antiviral, antibacterial, anticancer, antifungal, anti-inflammatory, antimalarial, antiurease, and antioxidant properties^19–27^. Thiosemicarbazone can regulate enzyme activity and is an attractive option for α-glucosidase inhibition. For example, Zahra et al. observed that coumarin-derived thiosemicarbazones had anti-diabetic properties^28^. Gul and his colleagues reported that *para*-substituted thiosemicarbazone derivatives had remarkable in-vitro α-glucosidase and α-amylase inhibitory activities^29^. Similarly, Bakherad et al. evaluated the α-glucosidase inhibitory activity of several thiosemicarbazone-indole hybrid compounds and documented their significant anti-diabetic properties, oral bio-availability, and good drug similarity^30^. However, no studies to date have explored the effect of phenyl carbamoyl methoxy substitutions on thiosemicarbazone scaffolds in the context of α-glucosidase inhibition.

Therefore, this study aimed to synthesise novel phenyl carbamoyl methoxy thiosemicarbazone derivatives and evaluate their potential as α-glucosidase inhibitors through biochemical assays, cytotoxicity screening, molecular docking, and molecular dynamics simulations.

## Materials and methods

### Material

pNPG (p-nitrophenyl Glucopyranoside), α-glucosidase (from *Saccharomyces cerevisiae* EC 3.2.1.20), acarbose, DMEM (Dulbecco’s modified Eagle’s medium), FBS (foetal bovine serum), RPMI (Roswell Park Memorial Institute), DMSO (dimethyl sulfoxide), MTT (3–4, 5 dimethyl thiazol- 2 yl)-2, 5- diphenyl tetrazolium bromide) were purchased from Sigma company^31^.

### General procedure for the synthesis of phenyl carbamoyl methoxy thiosemicarbazone

**Figure 1.**
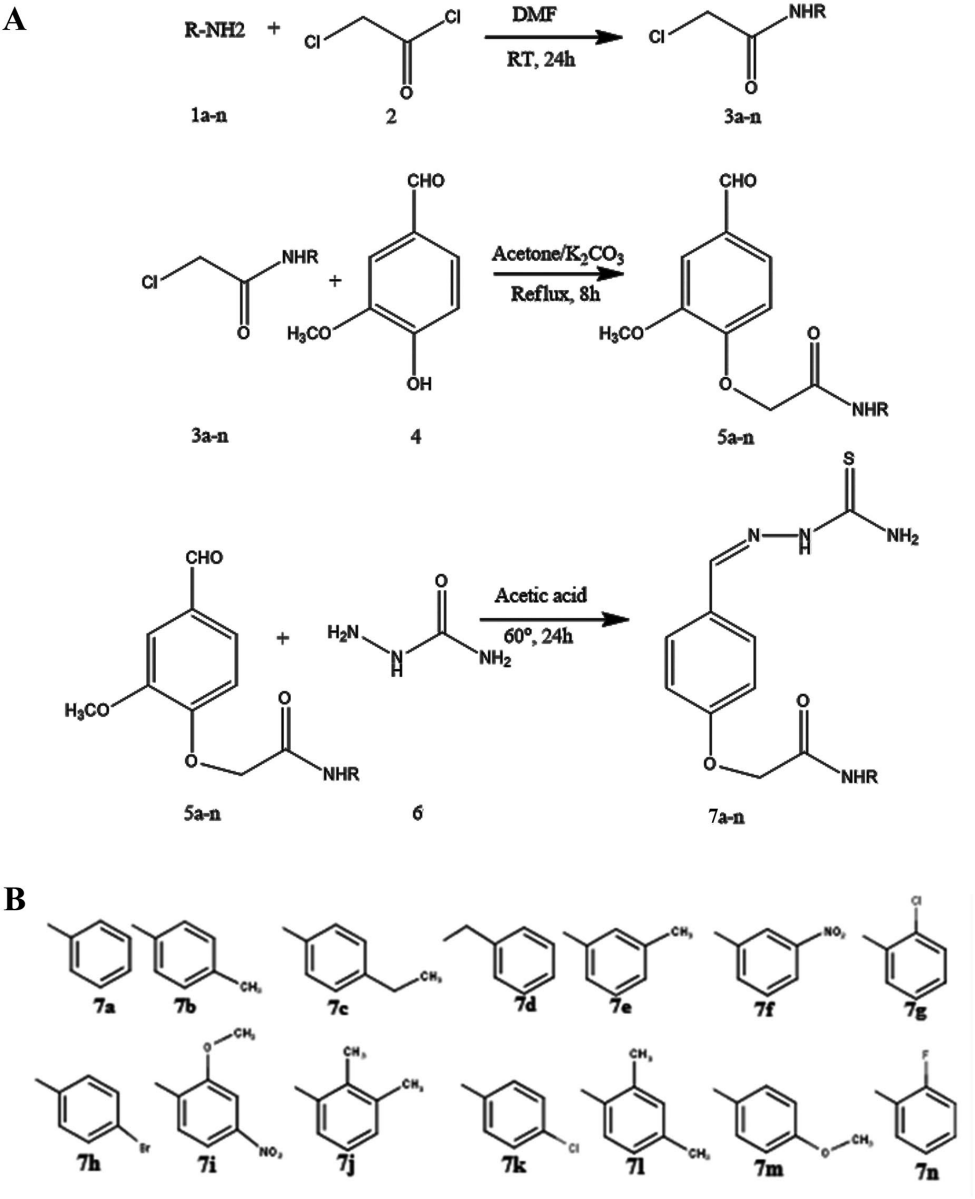
(A) General procedure for the synthesis of phenyl carbamoyl methoxy thiosemicarbazone derivatives **7a-n**. (B) R groups.

As presented in Figure 1A, choroacetyl chloride **2** (1 mmol) is carried out in dimethylformamide (DMF) at room temperature for 24 h. After completion of this reaction by checking on TLC, the precipitate was filtered off to obtain the acetamide derivatives **3a-n**. Then, a mixture of the acetamide derivatives **3a-n** and 4-hydro-3-methoxy benzaldehyde **4** were stirred in acetone in the presence of K_2_CO_3_ under reflux condition for 8 h. The observed participates were filtered and washed by water to give compounds **5a-n**. Then the reaction of thiosemicarbazide **6** and compounds **5a-n** is carried out in acetic acid at 60 °C for 24 h. The precipitates were filtered off and recrystallized by ethyl acetate and hexane to get the final products **7a-n** (Figure 1B).

The structures of all the novel synthesised inhibitors (compounds **7a-n**) were characterised by Electron impact mass spectra (EI-MS) ESI-MS, NMR ^1^H and ^13^C. NMR were measured with Bruker NMR Spectrometer DRX-500. Melting points were determined on a Barnstead Electrothermal (BI 9300) apparatus. All spectral pictures, including ESI-MS and NMR data, as well as the chemical structures of the synthesised compounds, have been included in the Supplementary Information file.

#### 2-{4-[(Z)-[(carbamothioylamino)imino]methyl]-2-methoxyphenoxy}-N-phenylacetamide (7a)

White powder; M.P: 223–225 °C; yield: 76%; ^1^H NMR (500 MHz, DMSO) δ 11.33 (s, 1H), 10.07 (s, 1H), 8.16 (s, 1H), 8.02 (s, 1H), 7.95 (s, 1H), 7.67 − 7.53 (m, 3H), 7.40 − 7.32 (m, 1H), 7.32 − 7.28 (m, 1H), 7.13 (dt, *J* = 8.3, 2.2 Hz, 1H), 7.07 (dd, *J* = 8.1, 6.7 Hz, 1H), 6.93 (dd, *J* = 8.3, 5.0 Hz, 1H), 4.72 (s, 2H), 3.86 (d, *J* = 3.3 Hz, 3H).^13^C NMR (126 MHz, DMSO) δ 178.22, 166.81, 149.98, 142.73, 137.81, 129.27, 129.17, 124.17, 122.29, 121.56, 119.96, 114.11, 109.81, 68.72, 56.36. ESI-MS m/z [M + H] ^+^: Calcd for C_17_H_18_N_4_O_3_S:359.

#### 2-{4-[(Z)-[(carbamothioylamino)imino]methyl]-2-methoxyphenoxy}-N-(4-methylphenyl) acetamide (7b)

White powder; M.P: 208–210 °C; yield: 81%;^1^H NMR (500 MHz, DMSO) δ 11.33 (s, 1H), 9.98 (s, 1H), 8.17 (s, 1H), 8.02 (s, 1H), 7.96 (s, 1H), 7.56 (d, *J* = 1.9 Hz, 1H), 7.45 (s, 1H), 7.38 (d, *J* = 8.1 Hz, 1H), 7.19 (t, *J* = 7.8 Hz, 1H), 7.14 (dd, *J* = 8.3, 1.9 Hz, 1H), 6.94 (d, *J* = 8.3 Hz, 1H), 6.89 (d, *J* = 7.5 Hz, 1H), 4.71 (s, 2H), 3.87 (s, 3H), 2.27 (s, 3H).^13^C NMR (126 MHz, DMSO) δ 178.22, 166.73, 149.66, 142.74, 138.75, 138.49, 129.10, 128.58, 124.87, 122.29, 120.47, 117.14, 109.81, 68.73, 56.38, 21.63. ESI-MS m/z [M + H] ^+^:Calcd for C_18_H_20_N_4_O_3_S:373.

#### 2-{4-[(Z)-[(carbamothioylamino)imino]methyl]-2-methoxyphenoxy}-N-(4-ethylphenyl) acetamide (7c)

White Brown powder; M.P: 243–246 °C; yield: 75%;^1^H NMR (500 MHz, DMSO) δ 11.33 (s, 1H), 9.98 (s, 1H), 8.16 (s, 1H), 8.04 − 8.00 (m, 1H), 7.96 (s, 1H), 7.56 (d, *J* = 2.0 Hz, 1H), 7.53 − 7.47 (m, 2H), 7.14 (dd, *J* = 8.7, 2.5 Hz, 3H), 6.94 (d, *J* = 8.3 Hz, 1H), 4.70 (s, 2H), 3.86 (s, 3H), 2.54 (q, *J* = 7.6 Hz, 2H), 1.14 (t, *J* = 7.6 Hz, 3H).^13^C NMR (126 MHz, DMSO) δ 178.22, 166.57, 149.99, 149.67, 142.74, 139.64, 136.50, 128.60, 128.44, 122.28, 120.07, 114.15, 109.81, 68.79, 56.37, 28.07, 16.12. ESI-MS m/z [M + H] ^+^: Calcd for C_19_H_22_N_4_O_3_S:387.

#### N-benzyl-2-{4-[(Z)-[(carbamothioylamino)imino]methyl]-2-methylphenoxy}acetamide (7d)

White powder; M.P: 219–221 °C; yield: 77%;^1^H NMR (500 MHz, DMSO) δ 11.34 (s, 1H), 8.47 (t, *J* = 6.1 Hz, 1H), 8.17 (s, 1H), 8.04 (s, 1H), 7.97 (s, 1H), 7.54 (d, *J* = 1.9 Hz, 1H), 7.30 (t, *J* = 7.4 Hz, 2H), 7.26 − 7.19 (m, 3H), 7.13 (dd, *J* = 8.3, 1.9 Hz, 1H), 6.92 (d, *J* = 8.3 Hz, 1H), 4.58 (s, 2H), 4.33 (d, *J* = 6.0 Hz, 2H), 3.82 (s, 3H).^13^C NMR (126 MHz, DMSO) δ 178.22, 168.12, 150.04, 149.57, 142.74, 139.61, 128.74, 128.69, 127.74, 127.31, 122.22, 114.42, 109.77, 68.75, 56.30, 42.41. ESI-MS m/z [M + H] ^+^: Calcd for C_18_H_20_N_4_O_3_S:373.

#### 2-{4-[(Z)-[(carbamothioylamino)imino]methyl]-2-methoxyphenoxy}-N-(3-methylphenyl) acetamide (7e)

White Brown powder; M.P: 198–200 °C; yield: 84%;^1^H NMR (500 MHz, DMSO) δ 11.33 (s, 1H), 9.99 (s, 1H), 8.16 (s, 1H), 8.02 (s, 1H), 7.96 (s, 1H), 7.55 (d, *J* = 1.9 Hz, 1H), 7.44 (d, *J* = 2.0 Hz, 1H), 7.38 (dd, *J* = 7.9, 2.2 Hz, 1H), 7.19 (t, *J* = 7.8 Hz, 1H), 7.14 (dd, *J* = 8.3, 2.0 Hz, 1H), 6.93 (d, *J* = 8.3 Hz, 1H), 6.89 (d, *J* = 7.5 Hz, 1H), 4.71 (s, 2H), 3.86 (s, 3H), 2.26 (s, 3H).^13^C NMR (126 MHz, DMSO) δ 178.21, 166.73, 149.97, 149.65, 142.75, 138.74, 138.50, 129.11, 128.57, 124.87, 122.30, 120.47, 117.14, 114.09, 109.80, 68.71, 56.37, 21.63. ESI-MS m/z [M + H] ^+^: Calcd for C_18_H_20_N_4_O_3_S:373.

#### 2-{4-[(Z)-[(carbamothioylamino)imino]methyl]-2-methoxyphenoxy}-N-(3-nitrophenyl) acetamide (7f)

White powder; M.P: 238–240 °C; yield: 79%; ^1^H NMR (500 MHz, DMSO) δ 11.33 (s, 1H), 10.62 (s, 1H), 8.64 (t, *J* = 2.2 Hz, 1H), 8.17 (s, 1H), 8.02 (s, 1H), 7.96 (s, 1H), 7.99 − 7.90 (m, 2H), 7.62 (t, *J* = 8.2 Hz, 1H), 7.56 (d, *J* = 1.9 Hz, 1H), 7.14 (dd, *J* = 8.3, 2.0 Hz, 1H), 6.95 (d, *J* = 8.3 Hz, 1H), 4.78 (s, 2H), 3.87 (s, 3H).^13^C NMR (126 MHz, DMSO) δ 178.23, 167.76, 150.02, 149.53, 148.46, 142.71, 140.00, 130.72, 128.74, 125.99, 122.27, 118.68, 114.26, 114.16, 109.85, 68.65, 56.36. ESI-MS m/z [M + H] ^+^: Calcd for C_17_H_17_N_5_O_5_S:404.

#### 2-{4-[(Z)-[(carbamothioylamino)imino]methyl]-2-methoxyphenoxy}-N-(2-chlorophenyl) acetamid (7 g)

White Brown powder; M.P: 240–242 °C; yield: 83%;^1^H NMR (500 MHz, DMSO) δ 11.35 (s, 1H), 9.59 (s, 1H), 8.18 (s, 1H), 8.07 − 7.98 (m, 1H), 7.97 (s, 1H), 7.78 (d, *J* = 23.1 Hz, 1H), 7.70 (d, *J* = 2.5 Hz, 1H), 7.59 − 7.54 (m, 2H), 7.44 (dd, *J* = 8.8, 2.5 Hz, 1H), 7.22 − 7.14 (m, 1H), 7.01 (d, *J* = 8.3 Hz, 1H), 4.81 (s, 2H), 3.87 (s, 3H).^13^C NMR (126 MHz, DMSO) δ 178.25, 167.25, 149.91, 148.94, 142.67, 133.74, 130.11, 129.40, 128.94, 128.36, 126.06, 125.16, 122.23, 114.25, 109.74, 68.35, 56.43. ESI-MS m/z [M + H] ^+^: Calcd for C_17_H_17_ClN_4_O_3_S:393.

#### N-(4-bromophenyl)-2-{4-[(Z)-[(carbamothioylamino)imino]methyl]-2-methoxyphenoxy} acetamide (7h)

White Brown powder; M.P: 245–247 °C; yield: 74%;^1^H NMR (500 MHz, DMSO) δ 11.33 (s, 1H), 10.23 (s, 1H), 8.17 (s, 1H), 8.02 (s, 1H), 7.96 (s, 1H), 7.62 − 7.53 (m, 3H), 7.49 (d, *J* = 8.8 Hz, 2H), 7.13 (dd, *J* = 8.3, 1.9 Hz, 1H), 6.93 (d, *J* = 8.3 Hz, 1H), 4.72 (s, 2H), 3.86 (s, 3H).^13^C NMR (126 MHz, DMSO) δ 178.22, 167.05, 149.99, 149.59, 142.73, 138.23, 132.08, 128.65, 122.27, 121.94, 115.81, 114.17, 109.82, 68.73, 56.36. ESI-MS m/z [M + H] ^+^: Calcd for C_17_H_17_BrN_4_O_3_S:437.

#### 2-{4-[(Z)-[(carbamothioylamino)imino]methyl]-2-methoxyphenoxy}-N-(4-methylphenyl) acetamide (7i)

Yellow powder; M.P: 258–260 °C; yield: 78%; ^1^H NMR (500 MHz, DMSO) δ 11.33 (s, 1H), 10.54 (s, 1H), 8.16 (s, 1H), 8.05 − 8.00 (m, 2H), 7.98 (s, 1H), 7.57 (d, *J* = 2.6 Hz, 2H), 7.36 (dd, *J* = 9.1, 3.0 Hz, 1H), 7.16 (dd, *J* = 8.3, 1.9 Hz, 1H), 7.00 (d, *J* = 8.3 Hz, 1H), 4.74 (s, 2H), 3.88 (s, 3H), 3.83(s, 3H).^13^C NMR (126 MHz, DMSO) δ 178.25, 167.45, 156.24, 150.04, 148.98, 142.69, 141.36, 129.00, 126.22, 125.23, 122.15, 121.84, 114.38, 109.81, 109.66, 68.47, 56.50, 56.39. ESI-MS m/z [M + H] ^+^:Calcd for C_18_H_19_N_5_O_6_S:434.

#### 2-{4-[(Z)-[(carbamothioylamino)imino]methyl]-2-methoxyphenoxy}-N-(2,3- dimethylphenyl) acetamide(7j)

White Brown powder; M.P: 238–240 °C; yield: 80%;^1^H NMR (500 MHz, DMSO) δ 11.34 (s, 1H), 9.41 (s, 1H), 8.17 (s, 1H), 8.04 (s, 1H), 7.97 (s, 1H), 7.59 − 7.55 (m, 1H), 7.29 (d, *J* = 7.9 Hz, 1H), 7.17 (d, *J* = 8.2 Hz, 1H), 7.06 (t, *J* = 7.7 Hz, 1H), 7.00 (t, *J* = 6.9 Hz, 2H), 4.75 (s, 2H), 3.87 (s, 3H), 2.24 (s, 3H), 2.07 (s, 3H).^13^C NMR (126 MHz, DMSO) δ 178.23, 166.86, 149.97, 149.44, 142.74, 137.47, 135.79, 130.68, 128.66, 127.45, 125.79, 123.00, 122.24, 114.10, 109.80, 68.57, 56.39, 20.60, 14.05. ESI-MS m/z [M + H]^+^: Calcd for C_19_H_22_N_4_O_3_S:387.

#### 2-{4-[(Z)-[(carbamothioylamino)imino]methyl]-2-methoxyphenoxy}-N-(4-chlorophenyl) acetamide (7k)

Yellow powder; M.P: 246–248 °C; yield: 83%; ^1^H NMR (500 MHz, DMSO) δ 11.33 (s, 1H), 10.23 (s, 1H), 8.17 (s, 1H), 8.02 (s, 1H), 7.96 (s, 1H), 7.68 − 7.60 (m, 2H), 7.55 (d, *J* = 2.0 Hz, 1H), 7.41 − 7.34 (m, 2H), 7.13 (dd, *J* = 8.3, 1.9 Hz, 1H), 6.93 (d, *J* = 8.3 Hz, 1H), 4.72 (s, 2H), 3.86 (s, 3H).

^13^C NMR (126 MHz, DMSO) δ 178.22, 167.03, 149.99, 149.60, 142.72, 137.81, 129.17, 128.65, 127.78, 122.27, 121.56, 114.17, 109.82, 68.72, 56.36. ESI-MS m/z [M + H]^+^: Calcd for C_17_H_17_ClN_4_O_3_S:393.

#### 2-{4-[(Z)-[(carbamothioylamino)imino]methyl]-2-methoxyphenoxy}-N-(2,4-dimethylphenyl) acetamide (7 l)

White Brown powder; M.P: 227–229 °C; yield: 82%;^1^H NMR (500 MHz, DMSO) δ 11.34 (s, 1H), 9.26 (s, 1H), 8.17 (s, 1H), 8.04 (s, 1H), 7.98 (s, 1H), 7.57 (s, 1H), 7.41 (d, *J* = 8.1 Hz, 1H), 7.17 (d, *J* = 8.2 Hz, 1H), 6.99 (m, 3H), 4.74 (s, 2H), 3.87 (s, 3H), 2.23 (s, 3H), 2.15 (s, 3H).^13^C NMR (126 MHz, DMSO) δ 178.23, 166.68, 149.94, 149.39, 142.75, 134.78, 133.43, 131.36, 131.16, 128.66, 127.08, 124.39, 122.25, 114.04, 109.77, 68.50, 56.39, 20.92, 17.83.ESI-MS m/z [M + H]^+^: Calcd for C_19_H_22_N_4_O_3_S:387.

#### 2-{4-[(Z)-[(carbamothioylamino)imino]methyl]-2-methoxyphenoxy}-N-(4-methoxyphenyl) acetamide (7 m)

White powder; M.P: 243–245 °C; yield: 81%;^1^H NMR (500 MHz, DMSO) δ 11.33 (s, 1H), 9.93 (s, 1H), 8.16 (s, 1H), 8.02 (s, 1H), 7.96 (s, 1H), 7.55 (d, *J* = 1.9 Hz, 1H), 7.51 (d, *J* = 8.6 Hz, 2H), 7.14 (dd, *J* = 8.3, 1.9 Hz, 1H), 6.95 (d, *J* = 8.3 Hz, 1H), 6.88 (d, *J* = 8.7 Hz, 2H), 4.68 (s, 2H), 3.86 (s, 3H), 3.71 (s, 3H).^13^C NMR (126 MHz, DMSO) δ 178.22, 166.33, 156.05, 150.00, 149.68, 142.76, 131.92, 128.60, 122.29, 121.59, 114.41, 114.17, 109.80, 68.82, 56.36, 55.68. ESI-MS m/z [M + H]^+^: Calcd for C_18_H_20_N_4_O_4_S:389.

#### 2-{4-[(Z)-[(carbamothioylamino)imino]methyl]-2-methoxyphenoxy}-N-(2-fluorophenyl) acetamide (7n)

White Brown powder; M.P: 239–241 °C; yield: 80%;^1^H NMR (500 MHz, DMSO) δ 11.34 (s, 1H), 9.76 (s, 1H), 8.17 (s, 1H), 8.04 (s, 1H), 7.98 (s, 1H), 7.94 (d, *J* = 8.8 Hz, 1H), 7.56 (s, 1H), 7.27 (m, 1H), 7.16 (m, 3H), 6.98 (d, *J* = 8.4 Hz, 1H), 4.81 (s, 2H), 3.87 (s, 3H).^13^C NMR (126 MHz, DMSO) δ 178.24, 167.23, 153.95 (^1^*J*_C-F_ = 245.7 Hz), 149.97, 149.42, 142.73, 128.76, 126.07 (^3^*J*_C_-_F_ = 7.56 Hz), 125.93 (^3^*J*_C_-_F_ = 11.34 Hz), 124.98 (^4^*J*_C_-_F_ = 3.78 Hz), 124.10, 122.25, 116.06 (^2^*J*_C_-_F_ =18.9 Hz), 114.30, 109.84, 68.52, 56.41. ESI-MS m/z [M + H] ^+^: Calcd for C_17_H_17_FN_4_O_3_S:377.

### Molecular docking study

To construct 3D structure of α-1–4-glucosidase a using homology modelling with the Swiss Model server, we used isomaltase from *saccharomyces cerevisiae* with a pdb code of 3A4A as the template due to its high sequence identity of 72.37%. This process involved aligning the target sequence of alpha-1-4-glucosidase with the template sequence of isomaltase to generate a model that predicts the 3D structure of the enzyme. This enabled us to study the enzyme’s structure-function relationship and design novel inhibitors or substrates with therapeutic applications. The 3D structure of the acarbose (PubChem CID: 444254) was downloaded directly from the PubChem database. The structures of all ligands were optimised using Gaussian 09 software with the B3LYP method and the 6–31 G** basis set. Molecular docking, an automatic process for predicting the interactions of ligands with their targets, was conducted using the AutoDock 4.2 software package^32^. The protein model was initially improved by adding hydrogens using the AutoDock builder module, and the Gasteiger charges were later added to the system. The macromolecule was kept rigid, and all the torsion angles in the small molecules could rotate freely. The grid box size and centre were set at 80 × 80 × 80 Å^3^ and 16.46, −9.09, and 19.77 for x-, y-, and z-coordinates to allow the ligand to rotate freely. Lamarckian genetic algorithm (LGA) method was employed with 200 separated docking calculations consisting of the maximum 25 000 000 energy evaluations, the maximum number of 27 000 generations, a mutation rate of 0.02, a crossover rate of 0.80, with cluster tolerance 2 Å, and population size of 150. The structure with the lowest free energy of binding in the highest-populated cluster was chosen as the optimal docking pose.

### Molecular dynamics simulation methods

This study investigated three systems, analysing α-glucosidase in its free state and its complex form with acarbose and **7e** compounds. Molecular dynamics^24^ simulations were performed using GROMACS 2022.6 software^33^ with the Amber99SB force field. The initial compound structures for MD simulations were obtained from the most favourable docking poses, identified by their minimum binding energies within the predominant clusters. Force field parameters for the acarbose and **7e** compounds were generated using the ACPYPE tool^31^, which depends on python scripts. Each system was solved in a triclinic water box with 7.94400 nm * 9.54700 nm * 8.92100 nm dimensions, containing 19 083 water molecules modelled with the TIP3P water model. The ionic strength was set to 0.15 M by adding 78 Na^+^ and 61 Cl^-^ ions to simulate physiological conditions to maintain charge neutrality. The simulation process had four distinct phases. The initial phase was focused on energy minimisation to relax the system and remove unfavourable forces acting on the atoms. The second and third phases, which included 1-nanosecond (ns) simulations in both canonical (NVT) and isothermal-isobaric (NPT) ensembles, stabilised the temperature at 310 K and the pressure at 1 bar while restraining the heavy atoms. In the final phase, these restraints were released, leading to a 150 ns simulation with a time step of 2 femtoseconds^11^. Notable physical properties were extracted from the recorded simulation trajectories. Energy minimisation was the first steepest descent method^34^. The subsequent phases were based on the Nose-Hoover thermostat and the Parrinello-Rahman barostat to maintain stable temperature and pressure^35^^,^^36^. Van der Waals interactions were calculated using the Lennard-Jones potential, while the particle mesh Ewald method was the basis for long-range electrostatics^37^. Covalent bond constraints were implemented through the LINCS algorithm^38^. For examining both the free enzyme and the enzyme-ligand complexes, several properties were investigated, including root mean square deviation (RMSD), root mean square fluctuation (RMSF), and radius of gyration(Rg)^39^. Additional analyses were conducted, including free energy landscape^40^, principal component analysis (PCA), and evaluations of hydrogen bonds. The secondary structure of both free and bound enzymes was evaluated using the DSSP method, and the binding energy between the ligands and the enzyme was calculated through the MMPBSA method^41^.

### In vitro α-glucosidase inhibitory activity

The method described by Azizian et al. with some modifications was used to assess αglucosidase inhibitory activity of novel derivatives of phenyl carbamoyl methoxy thiosemicarbazone^42^. The enzyme solution was prepared of αglucosidase (1 U/mL) in a 0.1 mM phosphate buffer (pH: 6.8). The final volume of assay solution in the 96-well plate was 200 µl, which was prepared in the following order: 20 µl of the enzyme solution was used to obtain a final enzyme activity of 0.1 U per well. 20 µl of various concentrations of novel inhibitors (1000, 500, 250, 125, 62.5, 31.25, and 15.62 µM) and potassium phosphate buffer (135 µl, pH = 6.8). The 96-well plate was incubated for 10 min at 37 °C and pNPG as substrate (25 µl, 4 mM) was added to each well of the plate and was incubated again for 30 min at 37 °C. Finally, absorbance was measured at 405 nm using a spectrophotometer (BioTek ELx808, USA). The assay solution without novel inhibitors and acarbose were used as the negative control and standard inhibitor, respectively. The IC_50_ values for the tested inhibitors were calculated using Microsoft Excel 2016 software and non-linear regression analysis.

### Kinetic study

Kinetic studies were conducted to determine the inhibition mechanism of the strongest novel inhibitor with the lowest IC_50_. To investigate the values of the Michaelis–Menten constant (Km), a Lineweaver-Burk plot was plotted for the enzyme in the presence of the inhibitor (63, 125, 250 µM) and in its absence (0 µM), using four different substrate concentrations (4, 6, 8, and 10 mM). The experimental inhibitor constant^42^ was made using secondary plots^42^.

### Cytotoxicity assay

To evaluate the cytotoxic effects of novel derivatives of phenyl carbamoyl methoxy thiosemicarbazone, an MTT assay was conducted using the non-small cell lung carcinoma cell line A549 and the MDA-MB-453 human breast cancer cell line. Both cell lines were obtained from the Pasteur Institute in Iran, and the assay was performed following the method of Jelena et al.^15^, with some minor modifications.

In short, 100 µl of A549 cells (1 × 10^5^) cultured in DMEM (Dulbecco’s modified Eagle’s medium) enriched with 10% foetal bovine serum (FBS) and 100 U/mL penicillin. 100 mg/mL streptomycin were added to each well in a 96-well plate, and they were incubated at 37 °C and 5% CO_2_ for 24 h. Also, 100 µl of MDA-MB-453 cells (1 × 10^5^) cultured in RPMI enriched with 10% FBS and 100 U/mL penicillin and 100 mg/mL streptomycin were added to each well in a 96-well plate and were incubated at 37 °C and 5% CO_2_ for 24 h. Subsequently, the medium in both cell lines was replaced with different concentrations (12.5, 25, 50, and 100 µM) of two derivatives of **7e** and **7 g**, and plates were incubated at 37 °C and 5% CO_2_ for 24 h. After that, 10 µl of MTT (3–4, 5 dimethyl thiazol- 2 yl)-2, 5- diphenyl tetrazolium bromide) (Sigma USA) dye (5 µg/mL in PBS) was added to each well and incubated for 4 h at 37 °C. After 4 h, the solution was removed from the cells, and 100 µl of DMSO (dimethyl sulfoxide) was added to the wells. The optical density of each well was measured at 570 nm using an ELISA reader (BioTek ELx808, USA). The wells containing more cells have a higher optical density (OD) than those with fewer cells. Therefore, the wells with more cells can be identified using the following equation and compared with the control sample.

$$\text{Toxicity}\%=\left(1-\frac{\text{mean}\;\text{OD}\;\text{of sample}}{\text{mean}\;\text{OD}\;\text{of}\;\text{control}}\right)*100$$

viability%=100−Toxicity %

PBS buffer and 1% Triton X-100 were negative and positive controls, respectively. All tests were conducted in triplicate.

### Statistical analysis

The experiments were done in triplicates (*n* = 3) and verified as mean ± SD. Raw data were logarithmically transformed before analysis to normalise the distribution and increase statistical reliability. All data were calculated and graphically plotted using Microsoft Excel 2016 software.

## Results and discussion

### In vitro α-glucosidase inhibitory activity

All the novel derivatives of phenyl carbamoyl methoxy thiosemicarbazone (**7a-n**) were evaluated for their inhibitory activity against α-glucosidase. The results of novel derivatives are shown in Table 1.

**Table 1.** α-Glucosidase inhibitory activity and binding energies of the synthesised novel derivatives of phenyl carbamoyl methoxy thiosemicarbazone.

No	R	IC_50_ (µM) ± SD	Binding Energy (KCal/mol)
**7a**	Phe	257 ± 0.4910	−8.38
**7b**	4-Methyl-Phe	151.18 ± 0.020	−8.09
**7c**	4-Ethyl-Phe	95.99 ± 0.273	−8.10
**7d**	Benzyl	216.42 ± 0.025	−9.14
**7e**	**3-Methyl-Phe**	**23.95 ± 0.038**	**−9.70**
**7f**	3-NO_2_-Phe	328.17 ± 0.083	−9.21
**7 g**	2-chloro-Phe	62.2 ± 0.411	−8.67
**7h**	4-Bromo-Phe	573.67 ± 0.043	−8.61
**7i**	2-OMethoxy,4-No_2_-Phe	125.9 ± 0.016	−8.09
**7j**	2,3-dimethyl-Phe	103.91 ± 0.028	−8.57
**7k**	4-chloro-Phe	442.41 ± 0.009	−8.38
**7l**	2,4-dimethyl-Phe	122.72 ± 0.098	−8.62
**7m**	4-OMethoxy-Phe	436.5 ± 0.062	−8.68
**7n**	2-Fluoro-Phe	95.65 ± 0.056	−8.56
**Acarbose**	–	634.21 ± 0.027	−7.70

Bold text indicates that, among the tested compounds, derivative 7e (3-Methyl-Phe) showed the highest inhibitory potency and strongest binding affinity toward α-glucosidase.

The tested derivatives exhibited a range of inhibitory potential, from moderate to strong, with IC_50_ values ranging from 23.95 ± 0.038 to 573.67 ± 0.043 µM, as compared to the standard drug acarbose (IC_50_ = 634.21 ± 0.027 µM). Among investigated derivatives, the most potent compounds were **7e** (IC50: 23.95 ± 0.038 µM), **7 g** (IC_50_ = 62.2 ± 0.411 µM), and **7n** (IC_50_ = 95.65 ± 0.056 µM), respectively. The lowest α-glucosidase inhibitory activity was observed for **7h**, with an IC_50_ value of 573.67 ± 0.043 µM.

The **7a** derivative (IC_50_ = 257 ± 0.491 µM) has a phenyl ring group without any substitution as the R group, which increases its inhibitory activity compared to acarbose. Replacing the phenyl ring with a benzyl ring in **7d** (IC_50_ = 216.42 ± 0.025 µM) led to a non-significant increase in α-glucosidase inhibitory activity. The benzyl group may increase inhibitory activity by increasing hydrophobic interactions, creating π-π interactions with aromatic amino acids in the enzyme’s active site.

In the derivative **7e** (IC_50_ = 23.95 ± 0.038 µM), the addition of a methyl (3-Methyl) group at position 3 in phenyl ring significantly enhanced its inhibitory activity against α-glucosidase compared to **7a**, which may be due to its better placement in of the enzyme’s active site. In the derivative of **7f**, NO_2_ substituted with the methyl group in **7e** (the most powerful derivative) demonstrated (IC_50_ = 328.17 ± 0.083 µM) less inhibitory activity compared to **7e**. This indicates that position 3 is not influential in the inhibitory activity, and inhibitory activity depends on the nature of the linking groups.

The inhibitory activity of compound **7b** (IC_50_ = 151.17 ± 0.02 µM) was found to be reduced when a methyl group was substituted at position 4, as compared to compound **7e** (which features a methyl group at position 3). This observation suggests a critical role for the methyl group’s binding position in determining the inhibitor’s overall efficacy.

Derivatives **7c** and **7b**, featuring methyl and ethyl groups, respectively, at position 4, demonstrated notable inhibitory potential when compared to acarbose. The enhanced hydrophobicity conferred by these alkyl substitutions (methyl and ethyl) likely contributes to improved hydrophobic interactions within the enzyme’s active site. The enhanced binding affinity of the derivatives to the enzyme culminates in a corresponding increase in enzyme inhibition. Conversely, the bulkier ethyl group may diminish inhibitory activity compared to the methyl group, likely due to steric hindrance within the enzyme’s active site.

Derivatives **7h** (IC_50_ = 573.67 ± 0.043 µM) and **7k** (IC_50_ = 442.41 ± 0.009 µM), characterised by Br and Cl halogen groups, respectively, at position 4, exhibit diminished inhibitory activity when compared to compounds **7b** and **7c**. This reduction in potency is likely attributable to unfavourable steric interactions induced by the halogen substituents. These interactions are hypothesised to impede the optimal orientation and accommodation of the inhibitor within the enzyme’s active site.

In the derivative **7 m** (IC_50_ = 436.5 ± 0.062 µM), the presence of an O-methoxy group leads to a substantial decrease in inhibitory activity when compared to **7b** and **7c**. Both **7b** and **7c** feature a methyl and an ethyl group, respectively, at 4 position of the phenyl ring. This observed reduction in inhibitory activity is likely attributable to steric hindrance caused by the oxygen atom within the O-methoxy group attached to the phenyl ring.

In the **7 g** derivative (IC_50_ = 62.2 ± 0.411 µM), adding a 2-Cl group at position 2 in the phenyl ring significantly enhanced its inhibitory α-glucosidase activity compared to acarbose. Similarly, in the derivative **7n** (IC_50_ = 95.65 ± 0.056 µM), a 2-F group at position 2 in the phenyl ring was added, which enhanced the inhibitory activity. It may be postulated that 2-Cl and 2-F groups, as small electron-donating groups, can improve inhibitory activity against α-glucosidase by forming strong hydrophobic and electrostatic interactions with the enzyme’s active site.

In derivatives of **7j** and **7 l** were added at positions 2, 3, and 2, 4 methyl groups, respectively. It was observed that the inhibitory activity α-glucosidase of **7j** was superior to that of **7 l**. It can be concluded that the exact effect of methyl depends on its position in the molecular structure and how it interacts with the enzyme’s active site.

Overall, all derivatives of phenyl carbamoyl methoxy thiosemicarbazone (**7a-n**) exhibited better α-glucosidase inhibitory potential compared to acarbose. However, adding different groups at various positions in the phenyl ring significantly influences the variation in α-glucosidase inhibitory activity. A molecular docking study was conducted to assess further the role of various structural features in interactions with the enzyme’s active site, as discussed below.

### Molecular docking study

MarvineSketch 5.8.3 ChemAxon software was used to plot and render the structure of novel compounds. Homology modelling with the Swiss Model server was utilised for the isomaltase from saccharomyces cerevisiae with pdb code of 3A4A. To clarify the interactions between novel derivatives of phenyl carbamoyl methoxy thiosemicarbazone and amino acids in the substrate-binding pocket of α-glucosidase at the molecular level, a molecular docking study was conducted using AutoDock 4.2.

**Figure 2.**
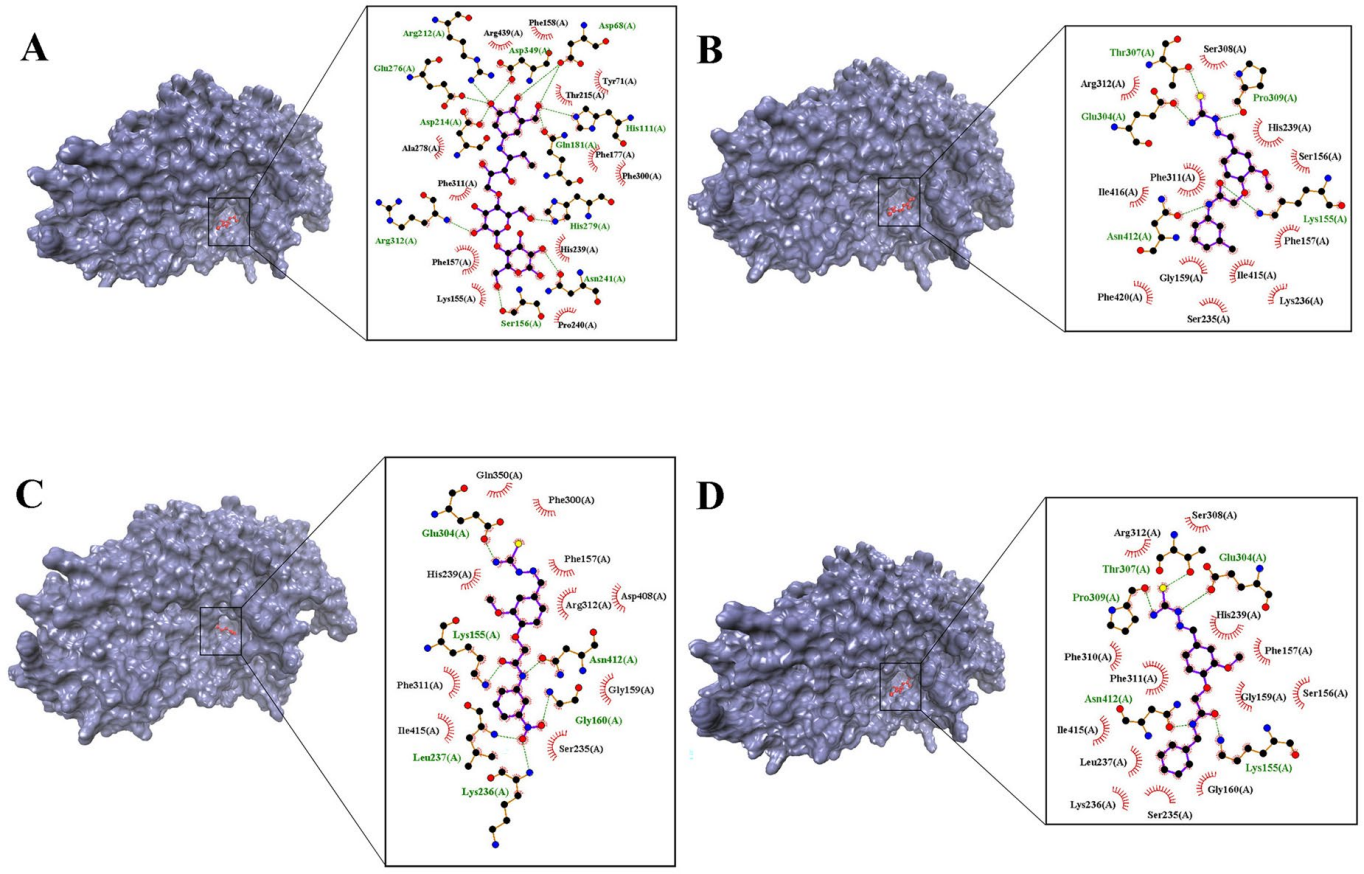
Binding interactions between (A) acarbose, (B) **7e**, (C) **7f**, and (D) **7d** with active site pocket of α-glucosidase enzyme.

As presented in Table 1, the binding energies of α-glucosidase with acarbose and 14 derivatives inhibitor compounds determined using AutoDock software. The binding energies of all 14 synthetic compounds derived from thiosmecarbazone had a stronger binding affinity for α-glucosidase than acarbose (due to lower binding energy than acarbose). However, **7e**, **7f**, and **7d** had the lowest binding energy among the synthesised derivatives. Figure 2 shows the interactions between acarbose, **7e**, **7f**, and **7d** with active site pocket of α-glucosidase enzyme. Figure 2A demonstrates that acarbose binds to the α-glucosidase enzyme through five hydrogen bonds with amino acids Gln181, Asp68, Glu276, Ser156, Asn241, Arg312, and Arg212 in the active site pockets. Additionally, acarbose interacts with the enzyme’s active site via several van der Waals interactions involving amino acids such as Lys155, Phe303, Ala278, and others. The binding energy of acarbose was calculated as −7.70 kcal/mol.

The most active compound, **7e**, was well accommodated inside the active site of a-glucosidase and established five hydrogen bonds with residues, namely Glu304, Thr307, Pro309, Lys155, and Asn412, in the active site pocket. Furthermore, van der Waals interactions were observed between compound five and residues Lys236, Arg312, His239, Phe 311, and Gly159 (Figure 2B). The binding energy of this compound was −9.70 kcal/mol.

Figure 2C demonstrates **7f** interaction with a-glucosidase via six hydrogen bonds with residues Lys155, Gly160, Lys236, Leu237, Glu304, and Asn412. Additionally, compound 6 was found to form several van der Waals interactions with residues such as Asp408, Arg312, His239, Phe300, and Gln350. The binding energy of this compound was determined as −9.21 kcal/mol.

The **7d** formed five hydrogen bonds with residues Lys155, Glu304, Thr307, Pro309, and Asn412 in the active site pocket of the enzyme. In addition, several van der Waals interactions were observed between compound 4 and various residues such as Ser308, Arg312, His239, Phe311, and Gly160 (Figure 2D). The binding energy of this compound was −9.14 kcal/mol.

### Molecular dynamic simulations result

**Figure 3.**
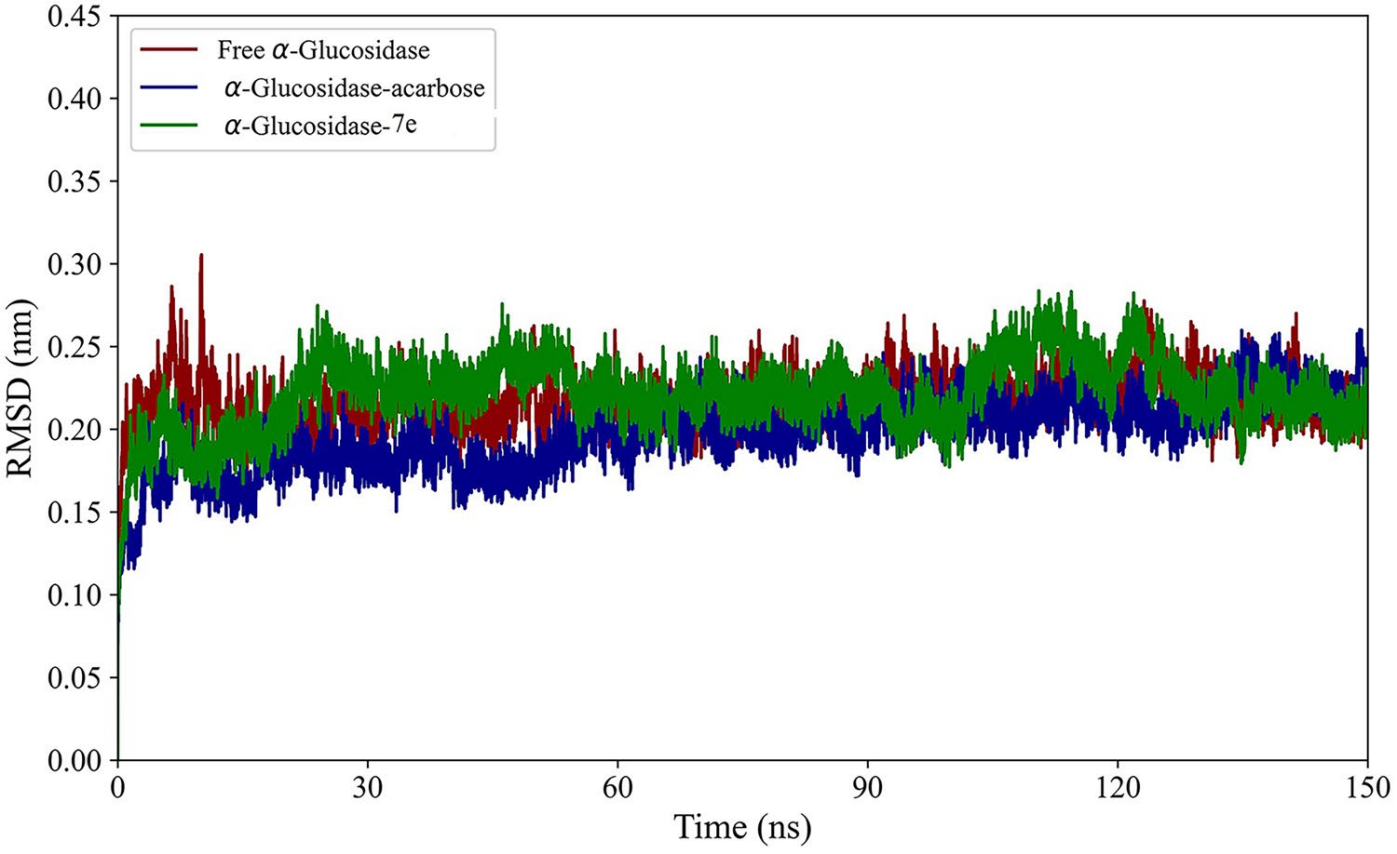
RMSD plots of the α-glucosidase in its free state and its complexes with acarbose and **7e** compounds over the 150 ns simulation period.

Following MD simulations, we first investigated the impact of acarbose and **7e** compounds binding on the stability, flexibility, dynamics, thermodynamics, and structure of α-glucosidase. To assess the stabilisation of α-glucosidase in its Free State and complex with acarbose and **7e**, we analysed the RMSD plots. Figure 3 demonstrates the RMSD plots for the backbone atoms of the enzyme in its unbound state and when it forms complexes with acarbose and **7e**. The results reveal minimal fluctuations throughout the simulation. Notably, during the final 30 ns of the simulation, all systems reached a stable state, displaying negligible fluctuations that indicate restricted conformational changes. Table 2 summarises the average RMSD, RMSF, and Rg values for the free enzyme and its complexes during the last 30 ns, reflecting their equilibrated states. Notably, the average RMSD value for the α-glucosidase **−7e** complex was slightly higher than that of the free enzyme, indicating that the binding interactions of **7e** slightly reduced the enzyme’s stability over time.

**Table 2.** Average and standard deviations of the RMSD, RMSF, and R_g_ for free and complex α-glucosidase during the last 30 ns.

System	Mean RMSD^43^	Mean RMSF^43^	Mean R_g_^43^
Free α-glucosidase	0.221 ± 0.013	0.0960 ± 0.0553	2.442 ± 0.008
α-Glucosidase–acarbose	0.221 ± 0.018	0.1094 ± 0.0636	2.456 ± 0.007
α-Glucosidase - 7e	0.246 ± 0.019	0.1072 ± 0.0653	2.452 ± 0.005

**Figure 4.**
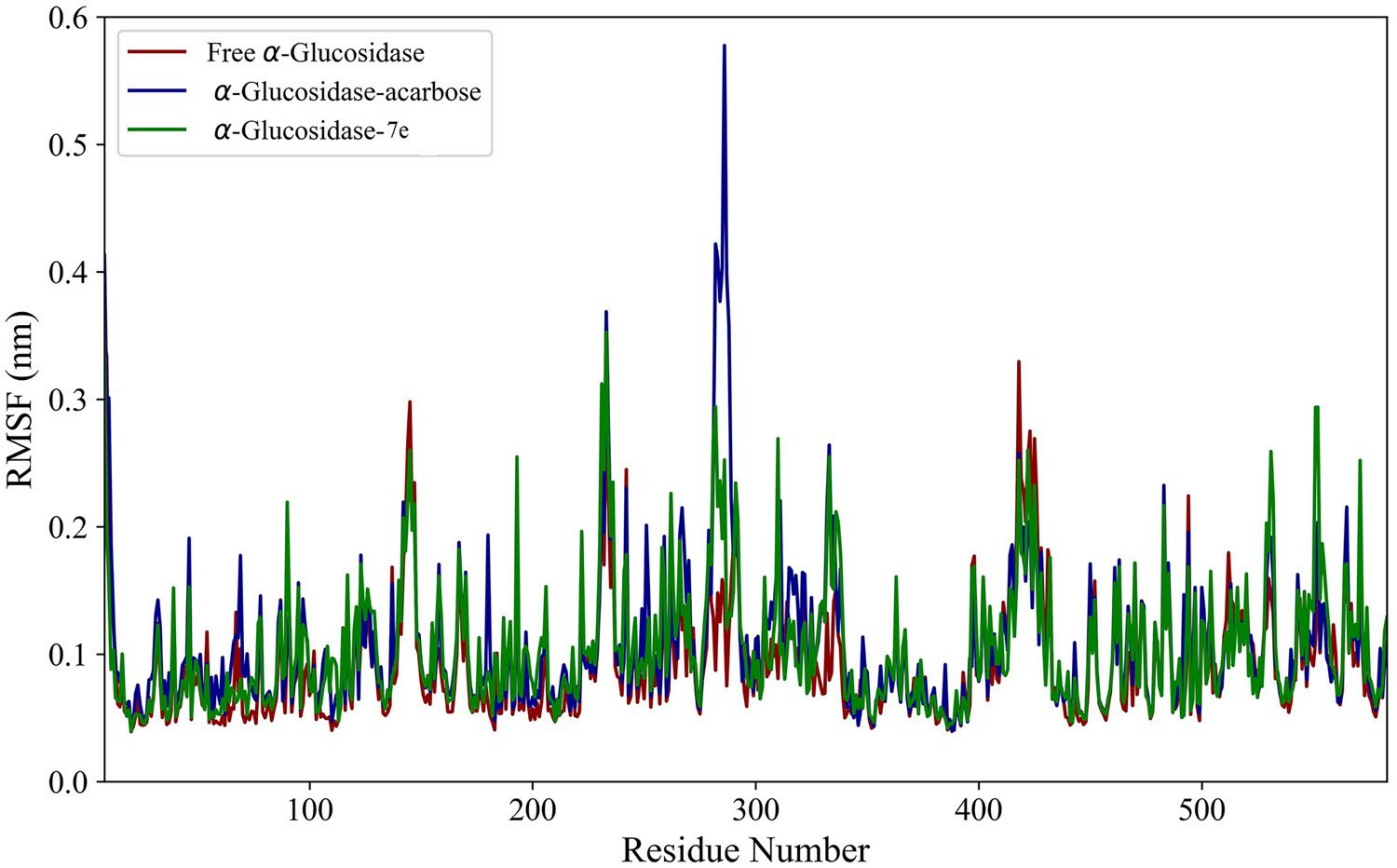
RMSF plots of α-glucosidase in its free state and its complexes with acarbose and **7e** Compounds.

The local flexibility of α-glucosidase was evaluated by comparing its RMSF in the presence and absence of compounds. As shown in Figure 4, the RMSF values remained consistently below 0.6 nm across all regions for the bound and unbound forms of α-glucosidase. This indicates restricted flexibility among the amino acids. Table 2 demonstrates a slight increase in the average RMSF for the bound states compared to the Free State. These findings indicate that binding acarbose and **7e** compounds enhanced the protein’s flexibility.

**Figure 5.**
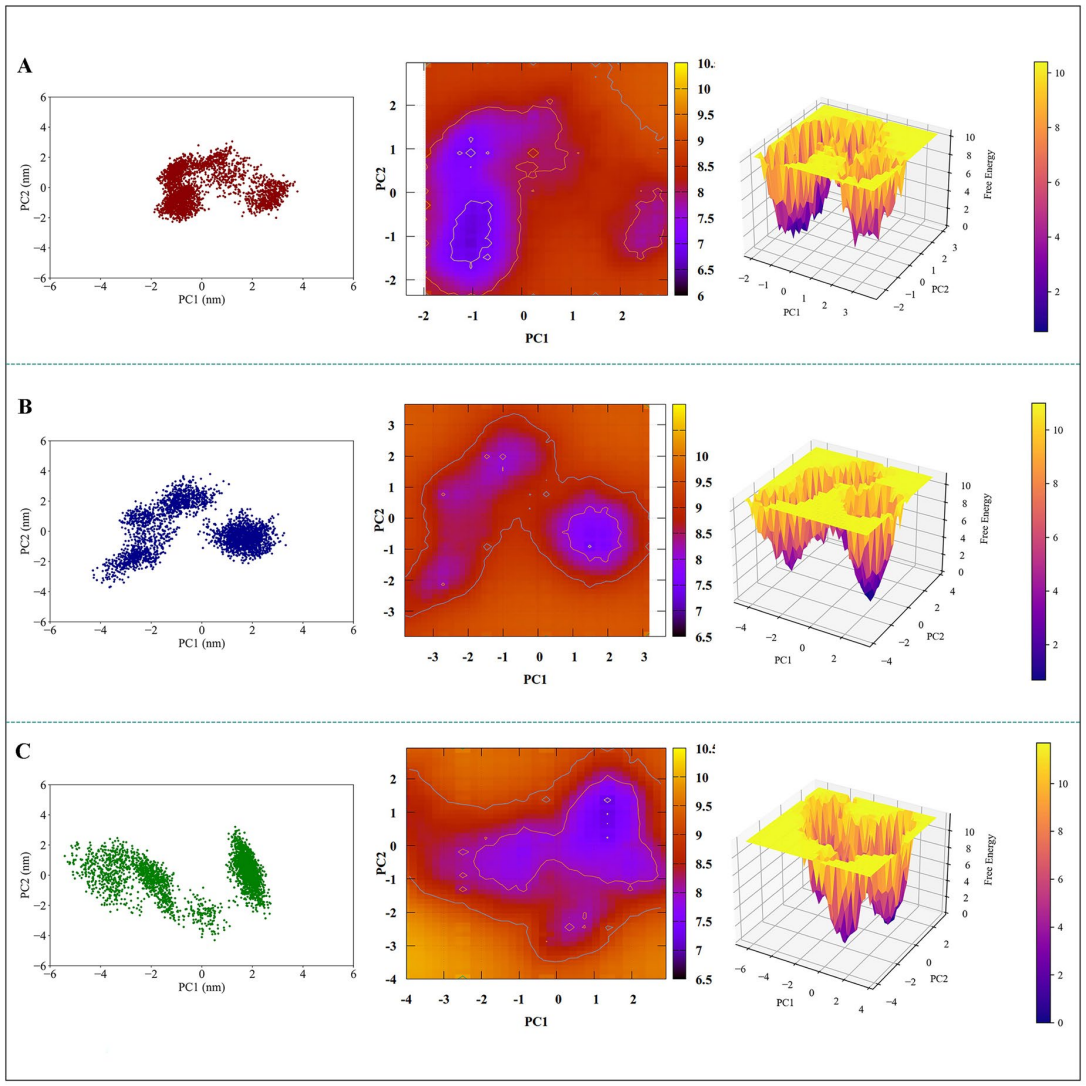
Analysis of PCA and FEL for (A) free α-glucosidase, (B) the α-glucosidase-acarbose system, and (C) the **α-**glucosidase-**7e** system. The left panel illustrates the PCA plot of enzyme atoms, the middle panel depicts the 2D FEL plot as a function of PC1 and PC2, and the right panel showcases the 3D FEL plot projected against PC1 and PC2.

PCA and FEL analyses were performed to investigate protein flexibility in depth, identify the principal components of protein motion, and predict its dynamic and thermodynamic properties. PCA offers a detailed examination of the structural properties of both free and bound proteins by analysing large-scale collective motions observed during MD simulations. This analysis focuses on the first principal components (PC1 and PC2), which are used to visualise the trajectory projections in phase space over the simulation period. The PCA plots (Figure 5) demonstrated distinct changes in patterns and occupied subspaces when comparing the complexes to the free enzyme. Precisely, the binding of acarbose and **7e** compounds resulted in an expansion of phase space occupancy and a reduction in the rigidity of the protein structure. These observations are consistent with the RMSF results, further supporting the findings. Additionally, the 2D and 3D FEL plots (Figure 5) demonstrated significant changes in the thermodynamic behaviour of the enzyme upon binding to acarbose and **7e**. The alterations in the FEL patterns highlight the influence of ligand binding on the energy landscape of the enzyme, offering insights into the thermodynamic stability and conformational dynamics of the protein-ligand systems. These analyses deepen our understanding of the structural and energetic changes induced by the binding of acarbose and **7e** compounds.

**Figure 6.**
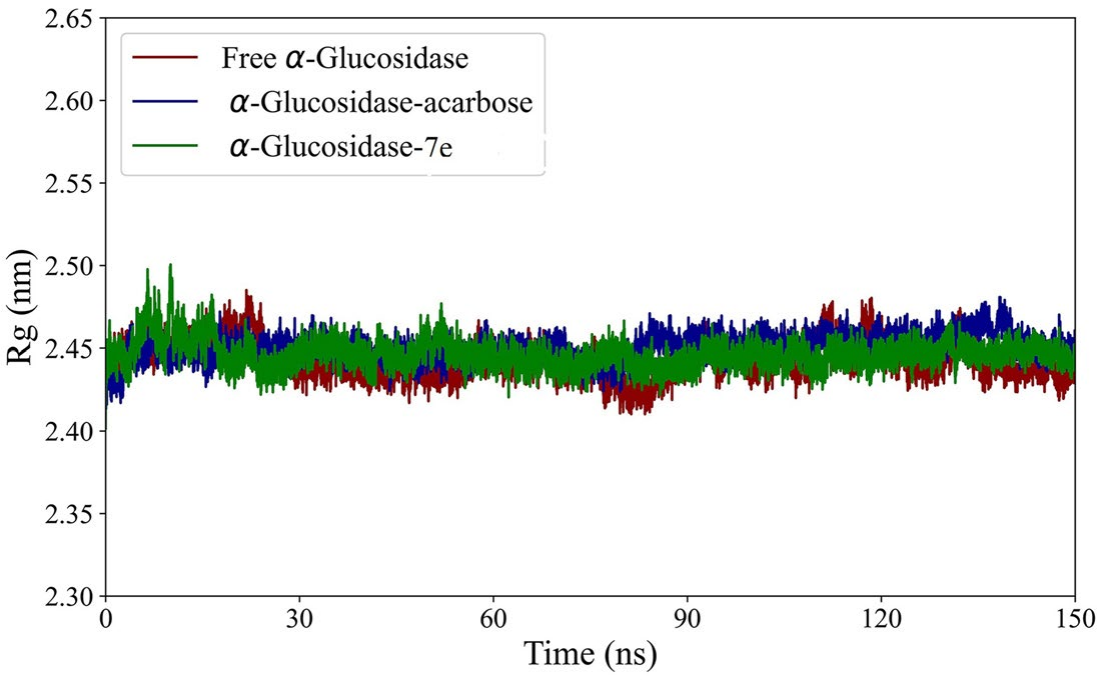
Rg plots of α-glucosidase in its free state and its complexes with acarbose and **7e** compounds over the 150 ns simulation period.

In the subsequent analysis, the secondary and tertiary structures of the enzyme were investigated. Rg plots were generated to assess the tertiary structures. As depicted in Figure 6, the Rg values, which reflect the compactness of the enzyme, remained stable throughout the simulation for all systems. Table 3 shows that the average Rg value for α-glucosidase in complexes with acarbose and **7e** was slightly higher than that of the free enzyme during the final 30 ns. This suggests that the binding of these compounds is associated with a slight reduction in the protein’s compactness and a minor degree of unfolding. Table 3 indicates that the decrease in enzyme compactness upon binding with acarbose and **7e** resulted in fewer intramolecular hydrogen bonds between enzyme atoms and increased hydrogen bonds between the enzyme and solvent atoms.

**Table 3.** Average values and standard deviations of hydrogen bonds formed between enzyme-enzyme (intramolecular interactions among α-glucosidase atoms) and enzyme-solvent during the final 30 ns of the simulation.

System	Enzyme–Enzyme	Enzyme -Solvent
Free α-glucosidase	488.056 ± 9.973	1141.692 ± 18.678
α-Glucosidase - acarbose	485.107 ± 10.606	1160.963 ± 19.147
α-Glucosidase - 7e	481.349 ± 10.749	1165.228 ± 24.421

**Figure 7.**
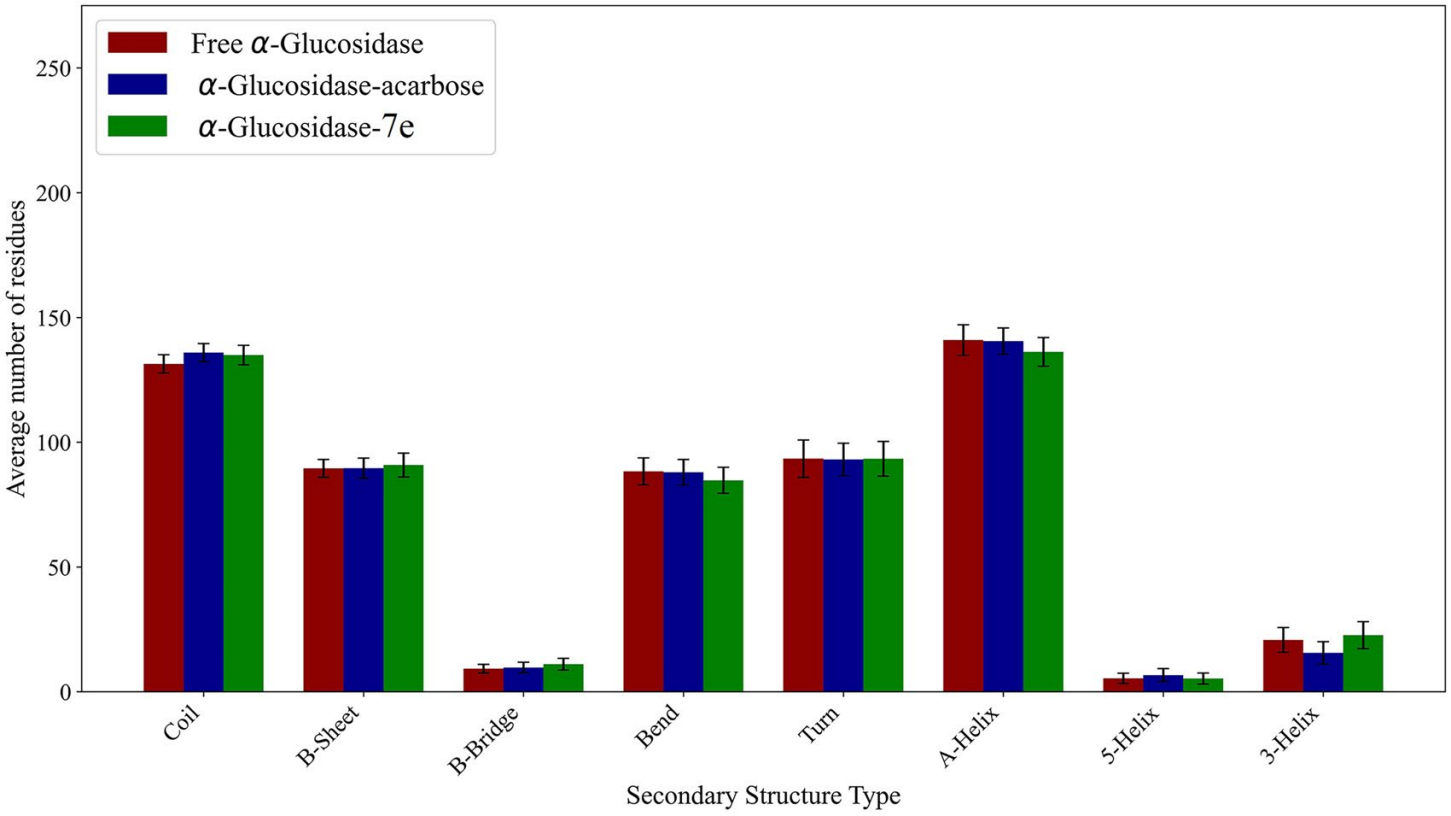
Average count of residues involved in the secondary structures of free and complex α-glucosidase over the final 30 ns.

The secondary structure analysis results are presented in Figure 7 and Table 4. As shown in the figure, the binding of the two compounds did not significantly alter the secondary structure of α-glucosidase. However, as demonstrated in Table 4, binding with acarbose and **7e** compounds led to a slight increase in β-bridge, β-sheet, and coil structures, while bend, turn, and α-helix structures decreased marginally. Specifically, the 5-helix structure increased due to binding with acarbose but decreased with **7e** binding, whereas the 3-helix structure increased with **7e** binding and decreased with acarbose binding. Notably, all these changes were highly subtle, indicating minimal impact on the overall secondary structure of the enzyme.

**Table 4.** Average number of the residues participating in the secondary structure of the α-glucosidase in the absence and presence of the acarbose and 7e compounds during the last 30 ns.

Structure	Free α-glucosidase	α-Glucosidase - acarbose	α-Glucosidase −7e
Coil	131.42 ± 3.64	135.92 ± 3.63	134.93 ± 3.93
B-Sheet	89.53 ± 3.53	89.59 ± 4.04	90.84 ± 4.78
B-Bridge	9.24 ± 1.76	9.70 ± 2.12	11.02 ± 2.34
Bend	88.30 ± 5.38	87.97 ± 5.09	84.72 ± 5.20
Turn	93.40 ± 7.48	93.08 ± 6.50	93.36 ± 6.93
A-Helix	140.98 ± 6.11	140.54 ± 5.23	136.21 ± 5.72
5-Helix	5.37 ± 2.07	6.65 ± 2.58	5.27 ± 2.21
3-Helix	20.78 ± 4.97	15.54 ± 4.50	22.65 ± 5.39

**Figure 8.**
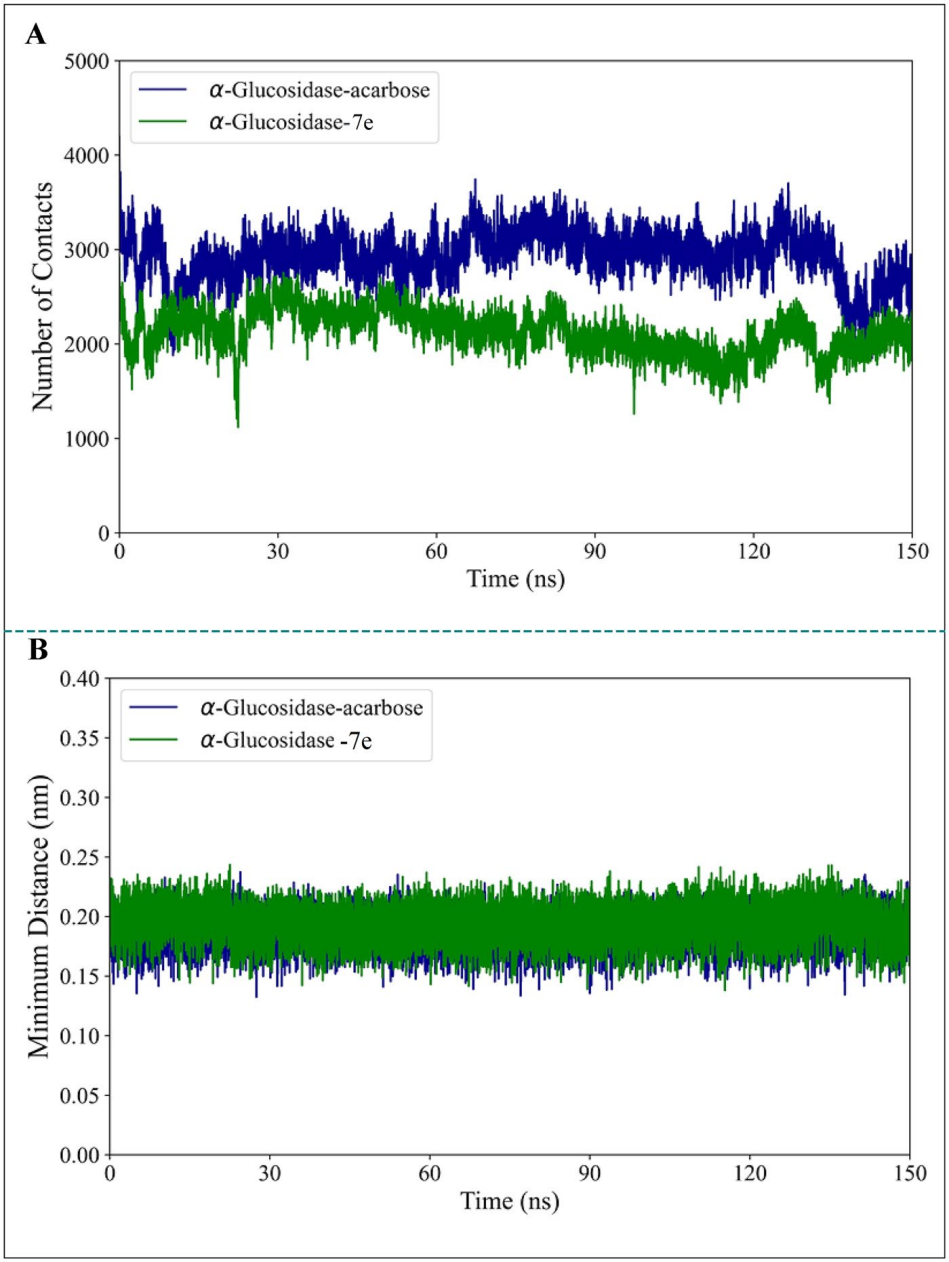
(A) Number of contacts and (B) minimum distance of acarbose and **7e** compounds with α-glucosidase over the 150 ns simulation period.

After investigating the behaviour of the α-glucosidase during the simulation time and assessment of the effects of acarbose and **7e** binding on the stability, flexibility, dynamics, thermodynamics, and structure of this enzyme, we focused on the behaviour of the compounds themselves. We evaluated the stability of acarbose and **7e** within the enzyme’s binding site. To accomplish this, we first analysed the number of contacts and the minimum distance of acarbose and **7e** with α-glucosidase. As depicted in Figure 8A, the number of contacts between acarbose and the enzyme was consistently higher than that of **7e** throughout the simulation. Moreover, for both the α-glucosidase-acarbose and α-glucosidase**-7e** systems, the number of contacts between the enzyme and the compound remained relatively stable, with no significant decline observed during the simulation. Figure 8B further illustrates that the minimum distance between both compounds and the enzyme showed only minor fluctuations with no substantial changes, suggesting stable binding interactions.

### MM-PBSA study

**Figure 9.**
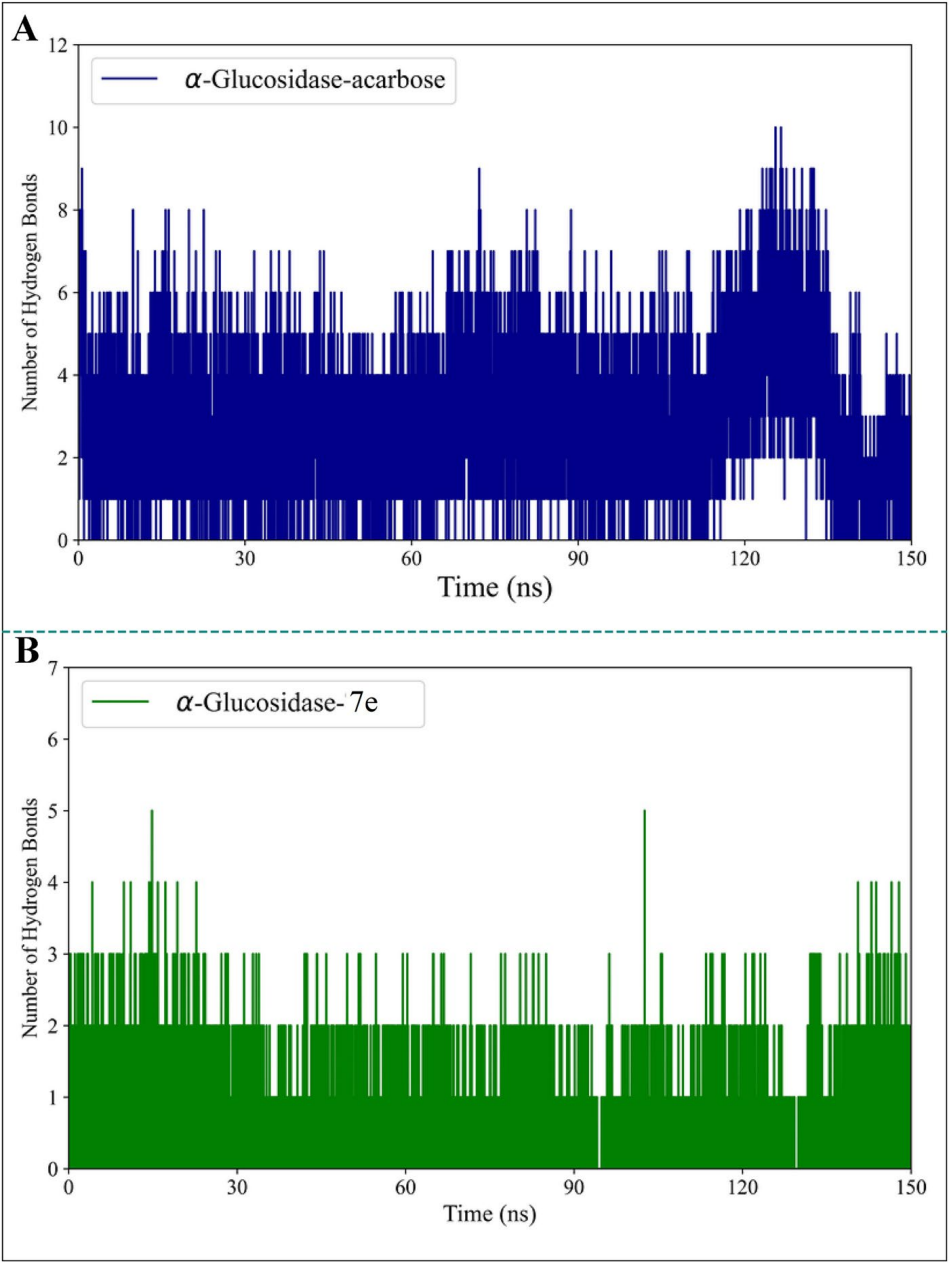
Number of hydrogen bonds of (A) acarbose and (B) **7e** compounds with α-glucosidase over the 150 ns simulation period.

To evaluate the stability of the binding, we also analysed the number of hydrogen bonds formed between α-glucosidase and the two compounds during the simulation time. As shown in Figure 9, both compounds consistently maintained stable hydrogen bonds with the enzyme throughout the simulation. The maximum number of hydrogen bonds observed was 10 for acarbose and 5 for **7e**. For a detailed assessment of binding energy and its components, the MM-PBSA method was employed, with the results presented in Table 5. The key factors influencing enzyme-ligand interactions were identified by examining 1000 frames from the final 30 ns of the trajectories. The results revealed favourable contributions from van der Waals, electrostatic, and SASA interactions, highlighting their critical role in binding. In contrast, polar solvation energies were consistently unfavourable for both complexes. Notably, van der Waals interactions were found to have a more significant impact on enzyme-ligand binding than other energy components, emphasising their importance in determining binding affinity. As indicated in Table 5, the binding energy between α-glucosidase and acarbose was more favourable than that with **7e**. In this study, we conducted MD simulations to investigate the effects of acarbose and **7e** compound binding on the behaviour of α-glucosidase during the 150 ns simulation time. The analysis of RMSD plots revealed that all systems reached a stable state during the final 30 ns of the simulation, with minimal fluctuations indicating restricted conformational changes. However, the α-glucosidase**-7e** complex exhibited higher average RMSD values than the free enzyme, suggesting that binding interactions with **7e** reduced the enzyme’s stability. RMSF analysis confirmed that the binding of acarbose and **7e** compounds had a marginal effect on enhancing the protein’s flexibility, as RMSF values remained consistently low across all regions. PCA and FEL analysis provided more profound insights into the structural and thermodynamic behaviour of the enzyme. The binding of acarbose and **7e** compounds led to an expansion of phase space occupancy and a reduction in protein rigidity, aligning with the RMSF results. Additionally, significant changes in the thermodynamic behaviour of the enzyme were observed upon ligand binding, as evidenced by alterations in the FEL patterns. Analysis of the enzyme’s secondary and tertiary structures revealed that the binding of acarbose and **7e** compounds resulted in a modest decrease in protein compactness, as indicated by a slightly higher radius of Rg values. This reduction in compactness was associated with fewer intramolecular hydrogen bonds and an increase in enzyme-solvent hydrogen bonds. Secondary structure analysis showed minimal changes, indicating that the overall secondary structure of the enzyme remained largely unaffected.

**Table 5.** Average and standard deviations of energy components for complexes analysed by MM-PBSA during the last 30 ns.

System	van der Waals energy (kJ/mol)	Electrostatic energy (kJ/mol)	SASA energy (kJ/mol)	Binding energy (kJ/mol)
α-Glucosidase acarbose	−222.385 ± 19.167	−44.324 ± 20.945	−25.102 ± 1.435	−141.065 ± 24.578
α-Glucosidase - 7e	−187.281 ± 15.347	−33.786 ± 14.913	−20.124 ± 1.245	−113.818 ± 15.213

The stability of acarbose and **7e** within the enzyme’s binding site was further evaluated by analysing the number of contacts, minimum distance, and hydrogen bonds. Acarbose exhibited a consistently higher number of contacts with the enzyme than **7e**, and both compounds maintained stable binding interactions throughout the simulation. Hydrogen bond analysis revealed that acarbose formed a maximum of 10 hydrogen bonds, while **7e** formed up to 5, underscoring the stronger binding affinity of acarbose. Binding energy calculations using the MM-PBSA method demonstrated that van der Waals interactions are crucial in enzyme-ligand binding for both complexes. Overall, the binding energy between α-glucosidase and acarbose was more favourable than that with **7e**, further supporting the stronger binding affinity of acarbose.

### Kinetic study

**Figure 10.**
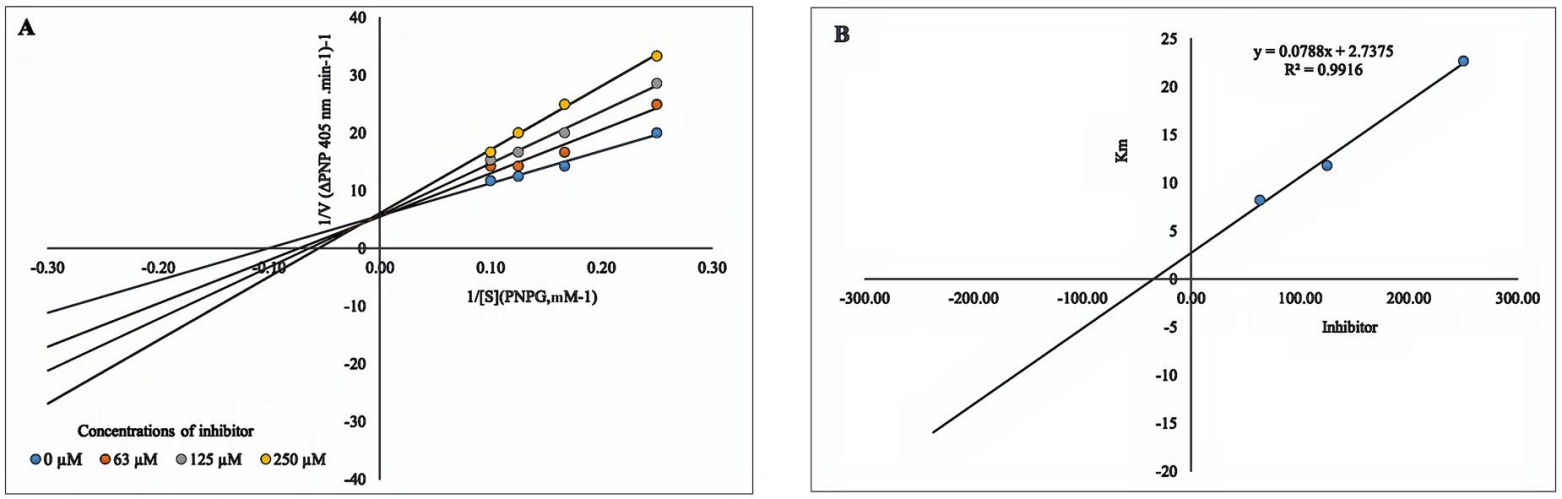
Kinetics of α-glucosidase inhibition of derivative **7e** (A) The Lineweaver–Burk plot in the absence and presence of different concentrations of **7e** (B) Secondary plot of Lineweaver-Burk plot.

To determine the inhibition mechanism of the novel derivatives of phenyl carbamoyl methoxy thiosemicarbazone on α-glucosidase, kinetic studies were conducted using the most potent compound, **7e**, which exhibited an IC_50_ value of 23.95 ± 0.038 µM. This was achieved using Lineweaver–Burk plots to determine the type of inhibition mechanism and the Km value. As shown in Figure 10A, the Km values for **7e** were determined at concentrations of 0, 63, 125, and 250 µM, resulting in values of 7.762, 8.224, 11.80, and 22.69, respectively. In the kinetic assay of α-glucosidase with synthetic inhibitor **7e**, the Km value gradually increased with increasing inhibitor concentration, while the Vmax value remained unchanged. The kinetic assay demonstrated that compound **7e**, featuring a 3-methyl group, functions as a competitive inhibitor of the α-glucosidase enzyme. This suggests a selective interaction at the enzyme’s active site. Recent research (Rafi et al.) supports this finding, demonstrating that analogous 3-methyl semicarbazone derivatives effectively inhibit α-glucosidase in a competitive and reversible manner^44^. This suggests their potential for managing postprandial hyperglycaemia while simultaneously reducing the risk of hypoglycaemia. This mode of inhibition is particularly advantageous for antidiabetic therapy, as it facilitates glucose-dependent modulation of enzyme activity. This inhibitory mechanism offers a significant advantage for antidiabetic treatments because it enables glucose-dependent regulation of enzyme activity. Furthermore, the reversible binding characteristic frequently observed in competitive inhibitors contributes to improved drug safety and offers greater precision in dosage management. These attributes highlight the promise of **7e** as a therapeutic agent for managing postprandial glucose levels in individuals with type 2 DM. As shown in Figure 10B, the Ki value for synthetic inhibitor **7e** was 34.76 µM.

### Cytotoxicity assay

**Figure 11.**
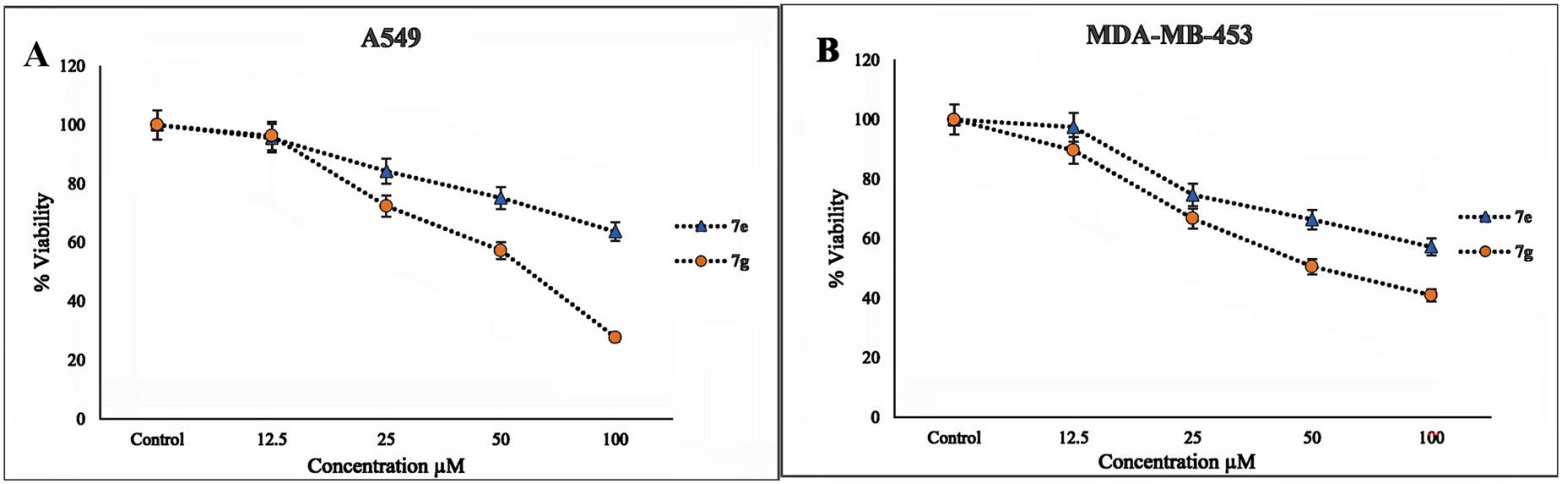
Cell survival rate diagram for **7e** and **7 g** (A) on A549 (B) on MDA-MB-453 cells lines.

The cytotoxicity effect of novel derivatives **7e** and **7 g** was tested towards selected non-small cell lung carcinoma (A549) and human breast carcinoma (MDAMB-453). The cell viability rate was evaluated by MTT test following 24 h of exposure to **7e** and **7 g**. Figure 11 shows the cell survival rate diagram for the novel derivatives **7e** and **7 g** on A549 (Figure 11A) and MDA-MB-453 cells (Figure 11B). The cell viability rates for **7e** against the A549 cell line at concentrations of 12.5, 25, 50, and 100 µM were 95. 50%, 84.26%, 75.16%, and 63.7%, respectively. Also, the cell survival rates of the MDAMB-453 cell line in the presence of different concentrations of **7e** (12.5, 25, and 50 µM) were 97.39%, 74.65%, 66.34%, and 57.18%, respectively.

Similarly, the percentage of cell viability of the A549 cell line in the presence of different concentrations of **7 g**, 12.5, 25, 50, and 100 µM were 96.250%, 72.38%, 57.29%, and 27.7%, respectively. Finally, the percentage of cell viability of MDA-MB-453 cells decreased (89.62%, 66.70%, 50.56%, and 40.97%) with increasing concentrations of **7 g** (12.5, 25, 50, and 100 µM).

## Conclusion

In conclusion, we presented a novel series of phenyl carbamoyl methoxy thiosemicarbazone derivatives, all exhibiting good to excellent α-glucosidase inhibitory activity. Among them, the derivative **7e** (the addition of a methyl group at position number 3 in the phenyl ring) showed the most significant α-glucosidase inhibitory potency with an IC_50_ value of 23.95 ± 0.038 µM compared to acarbose as a standard drug (IC_50_ = 634.21 ± 0.027 µM). The kinetic study for this compound showed that the inhibition mechanism was competitive. Docking studies presented that derivative 7e could interact with several amino acids in the active site of the α-glucosidase enzyme through electrostatic interactions and hydrogen bonds. Also, molecular dynamic simulations revealed that **7e** could affect the stability, flexibility, dynamics, thermodynamics, and structure of α-glucosidase enzymes such as acarbose. The derivative **7e** did not possess important cytotoxic activity against A549 and MDA-MB-453 cell lines.

The promising our results of derivative 7e in α-glucosidase inhibitory activity and in silico analysis could introduce a lead candidate for antidiabetic drug development.

## Supplementary Material

supporting information file.pdf

## Data Availability

The data used to support the findings in this study are available from the corresponding author upon reasonable request.
