# Supplementary material for: In vitro α-glucosidase inhibition, molecular dynamics and docking study of phenyl carbamoyl methoxy thiosemicarbazone derivatives as potential anti-diabetic agents
Source: J Enzyme Inhib Med Chem. 2025 Nov 4;40(1):2580515. doi: 10.1080/14756366.2025.2580515 (PMC12587786; doi:10.1080/14756366.2025.2580515)

^{1}H NMR

2-{4-[(Z)-[(carbamothioylamino)imino]methyl]-2-methoxyphenoxy}-N-phenylacetamide (7a)

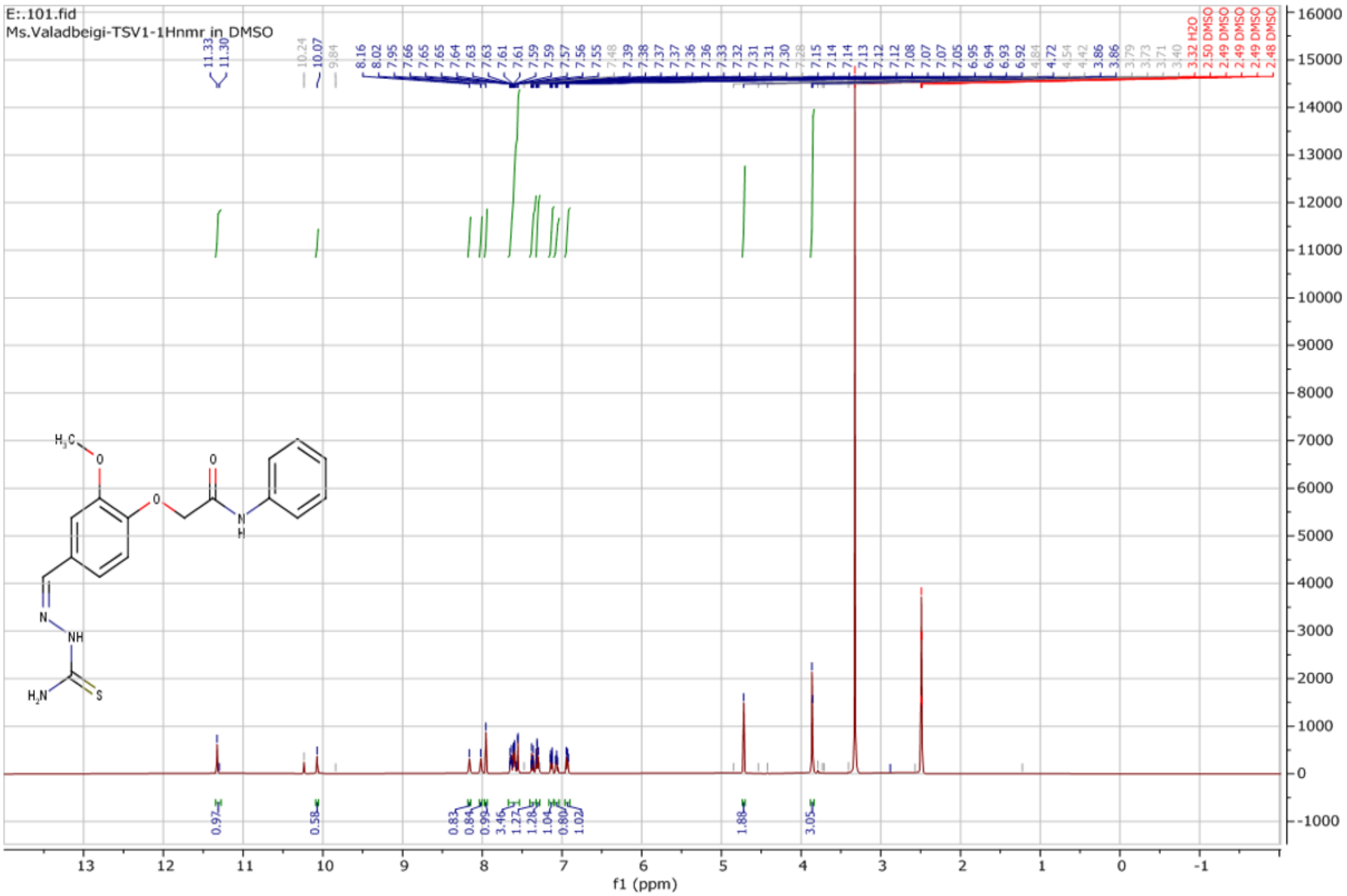

^{1}H NMR of 7a

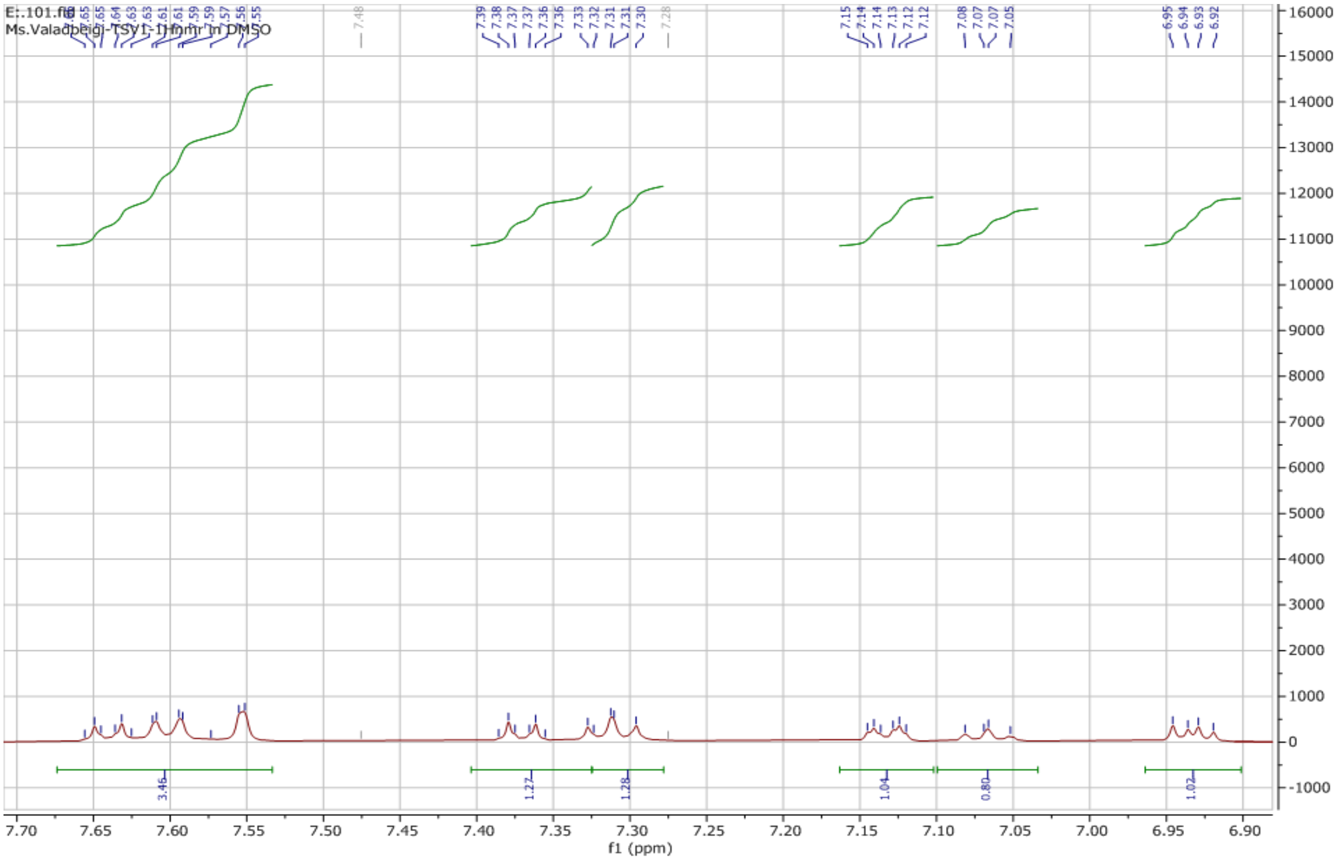

^{13} C NMR of 7a

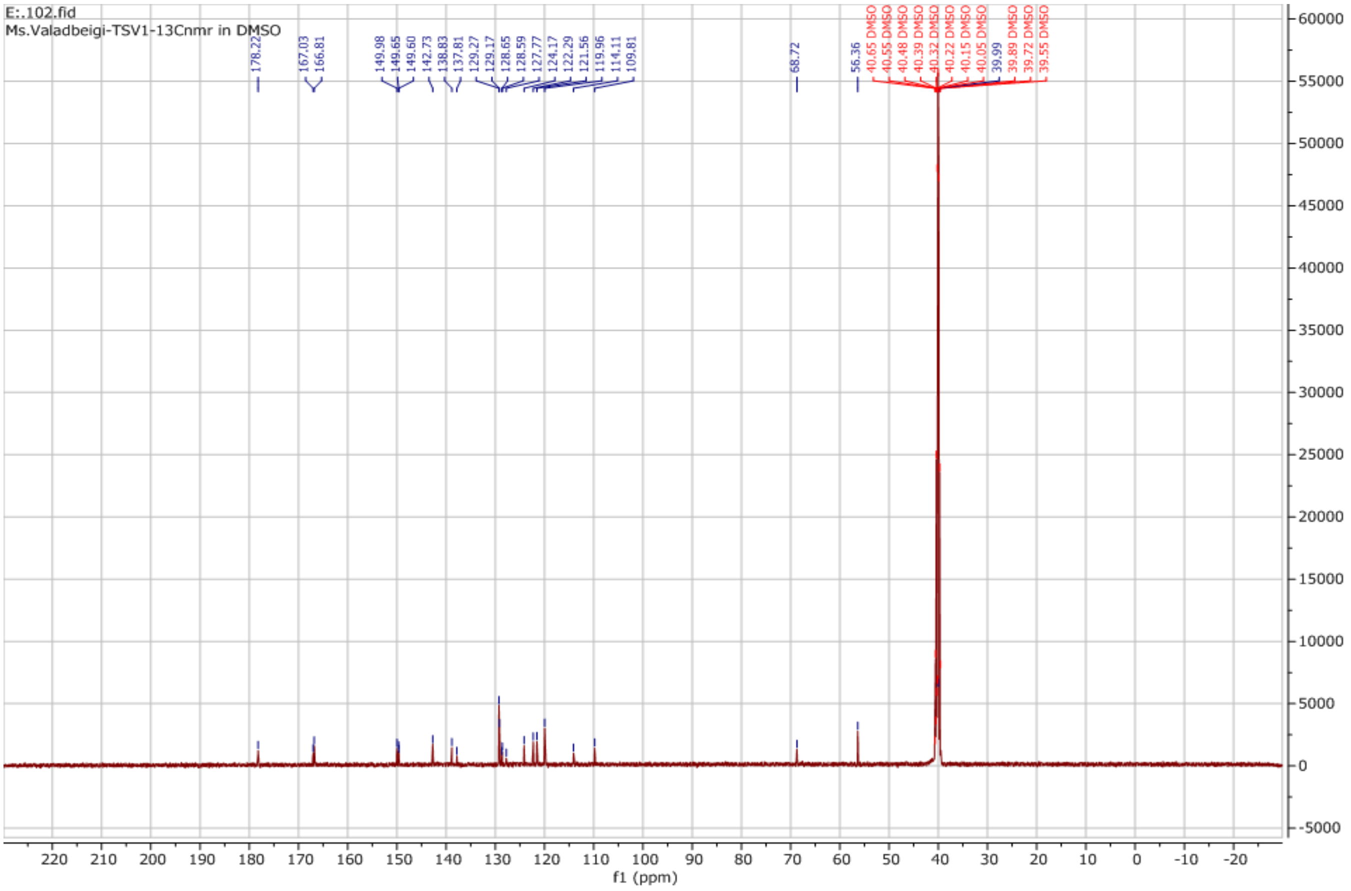

Mass spectra of 7a

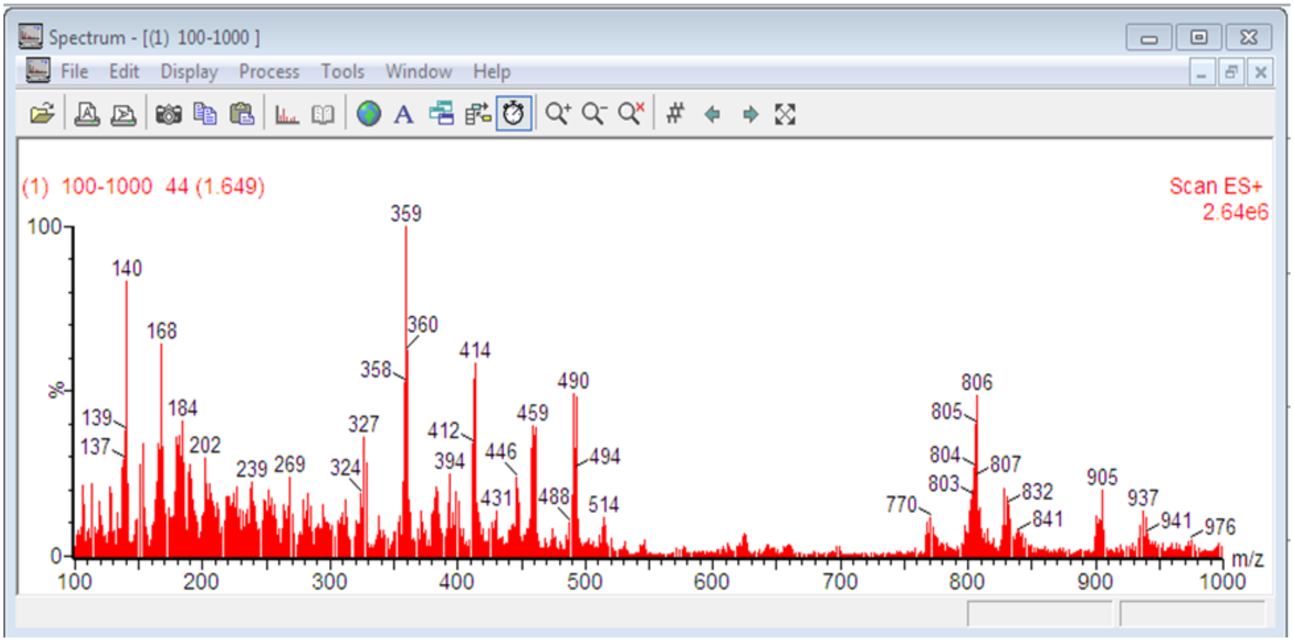

^{1}H NMR

2-{4-[(Z)-[(carbamothioylamino)imino]methyl]-2-methoxyphenoxy}-N-(4-methylphenyl)acetamide (7b)

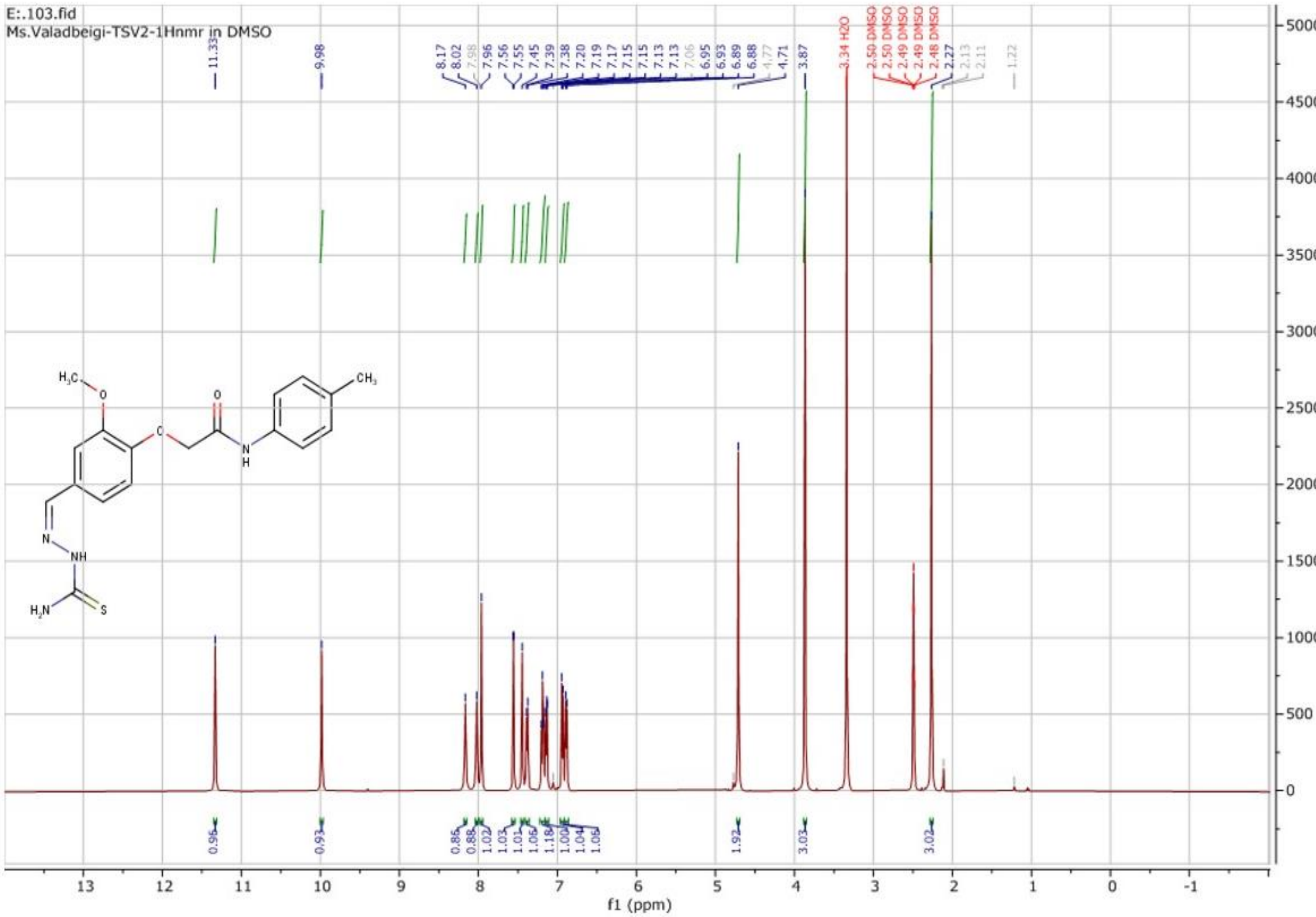

H[1] NMR of 7b

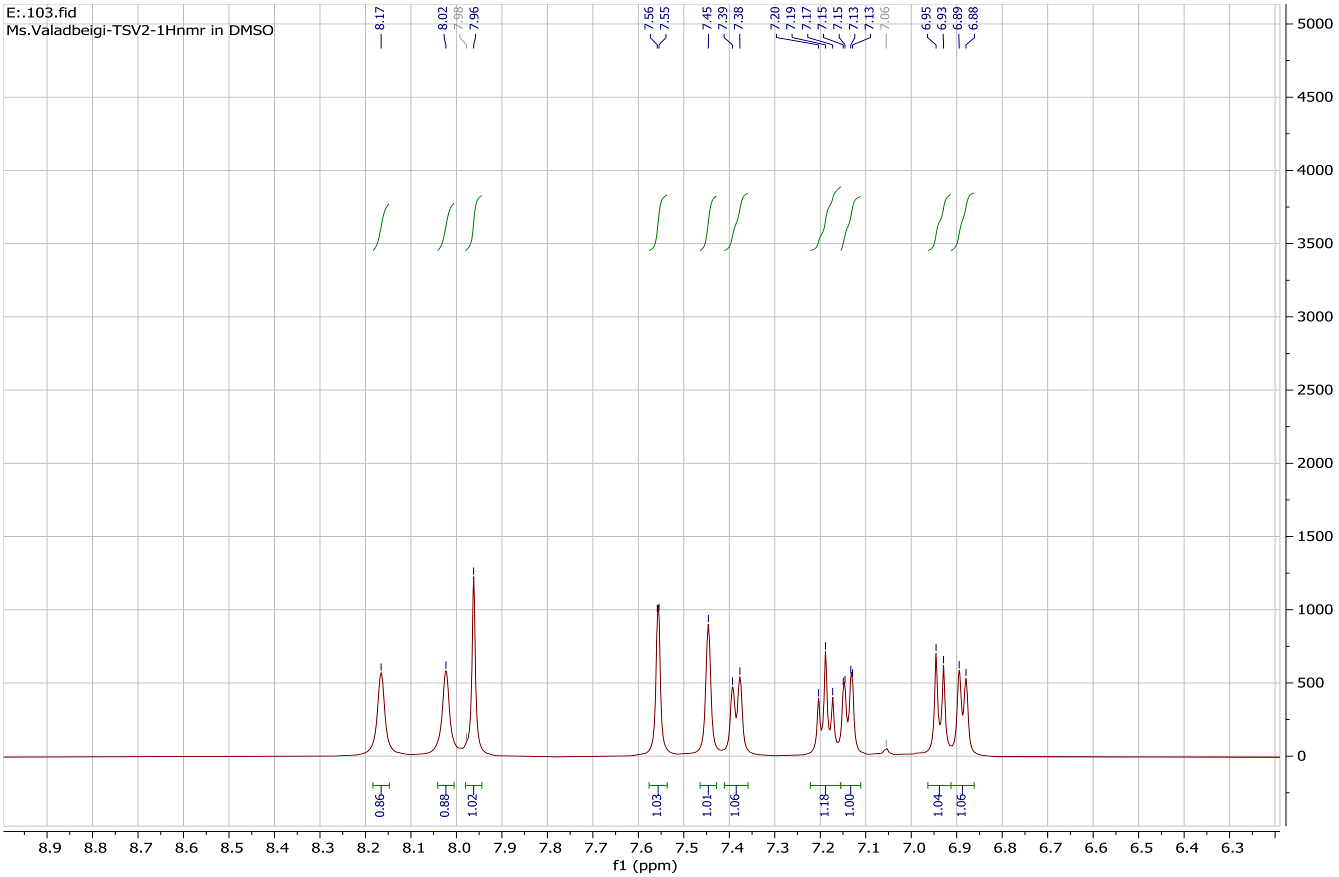

E:.103.fid
Ms.Valadbeigi-TSV2-1Hnmr in DMSO
8.17
8.02
7.98
7.96
7.56
7.55
7.45
7.39
7.38
7.20
7.19
7.17
7.15
7.15
7.13
7.13
7.06
6.95
6.93
6.89
6.88
0.86
0.88
1.02
1.03
1.01
1.06
1.18
1.00
1.04
1.06
f1 (ppm)

^{13}C NMR of 7b

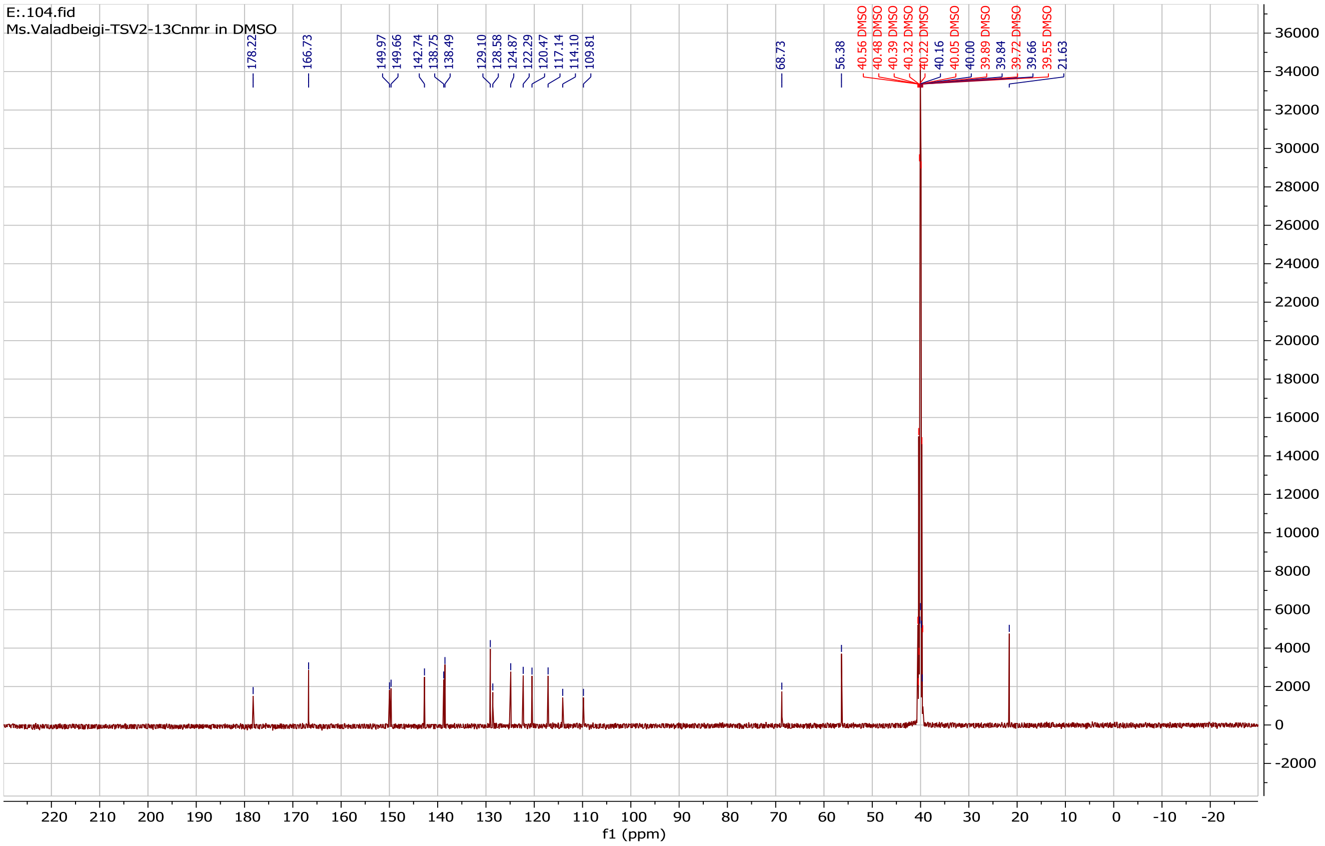

Mass spectra of 7b

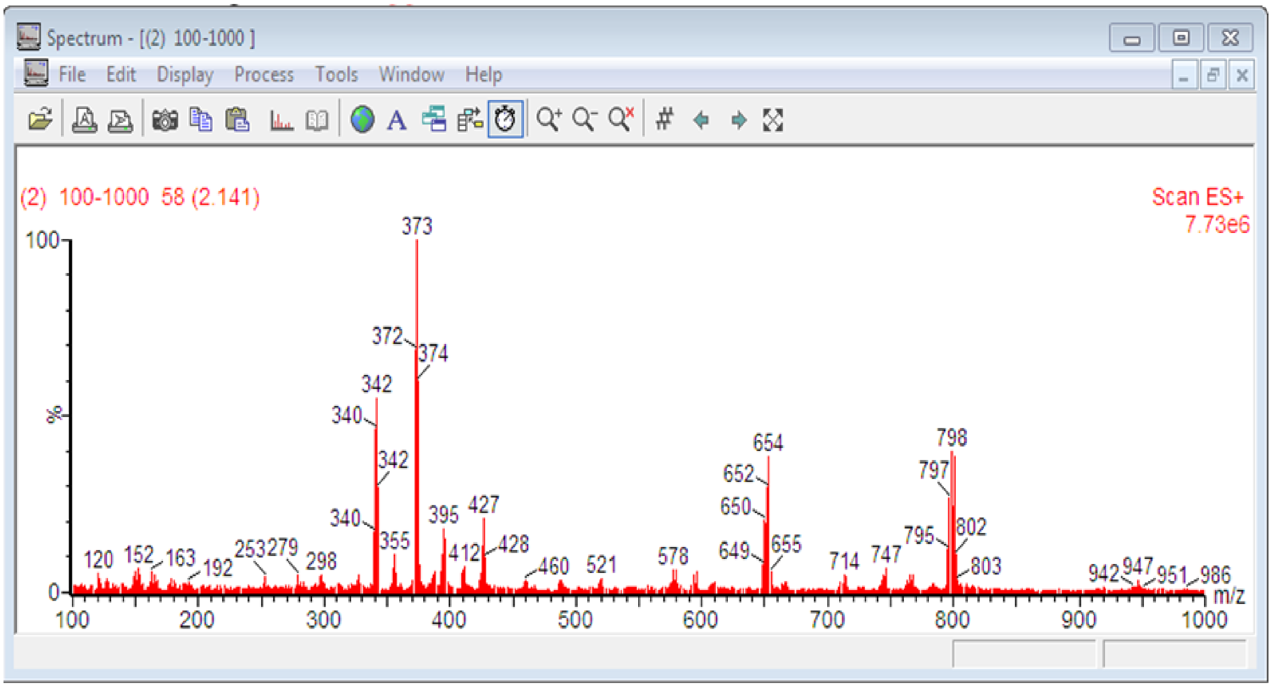

[1] HNMR

2-{4-[(Z)-[(carbamothioylamino)imino]methyl]-2-methoxyphenoxy}-N-(4-ethylphenyl) acetamide (7c)

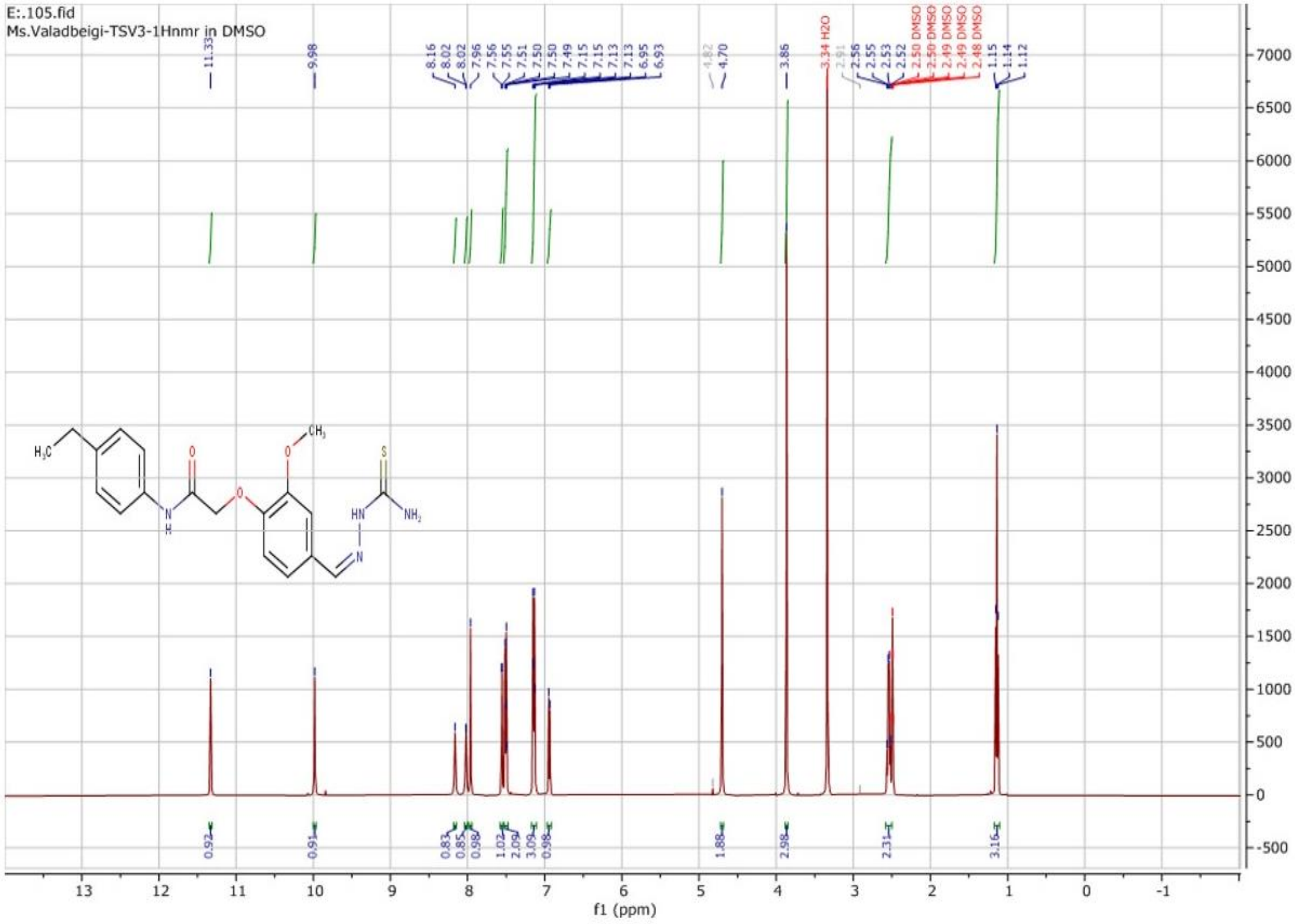

H[1] NMR of 7c

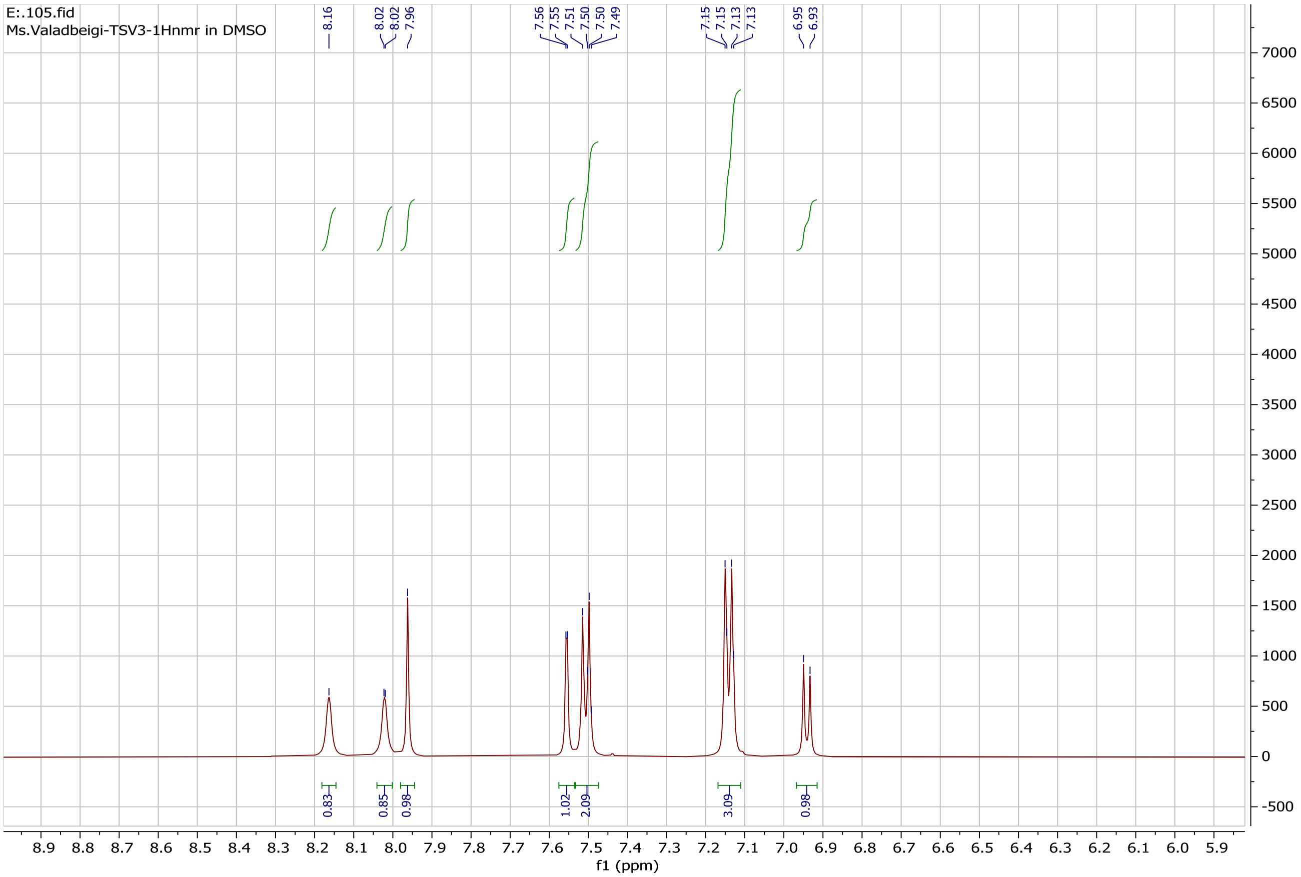

E:.105.fid
Ms.Valadbeigi-TSV3-1Hnmr in DMSO
8.16
8.02
8.02
7.96
7.56
7.55
7.51
7.50
7.50
7.49
7.15
7.15
7.13
7.13
6.95
6.93
0.83
0.85
0.98
1.02
2.09
3.09
0.98
f1 (ppm)

^{13}C NMR of 7c

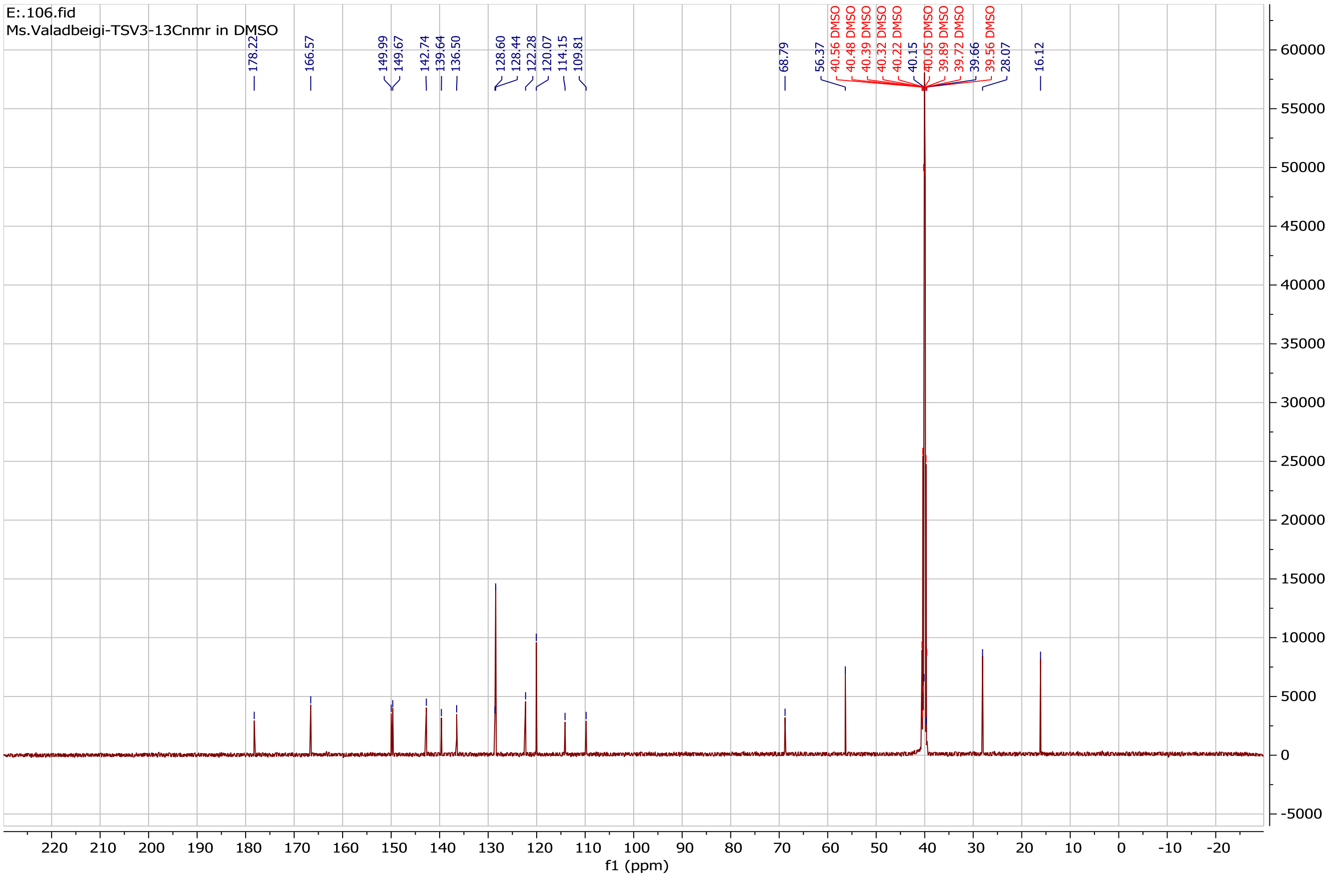

Mass spectra of 7c

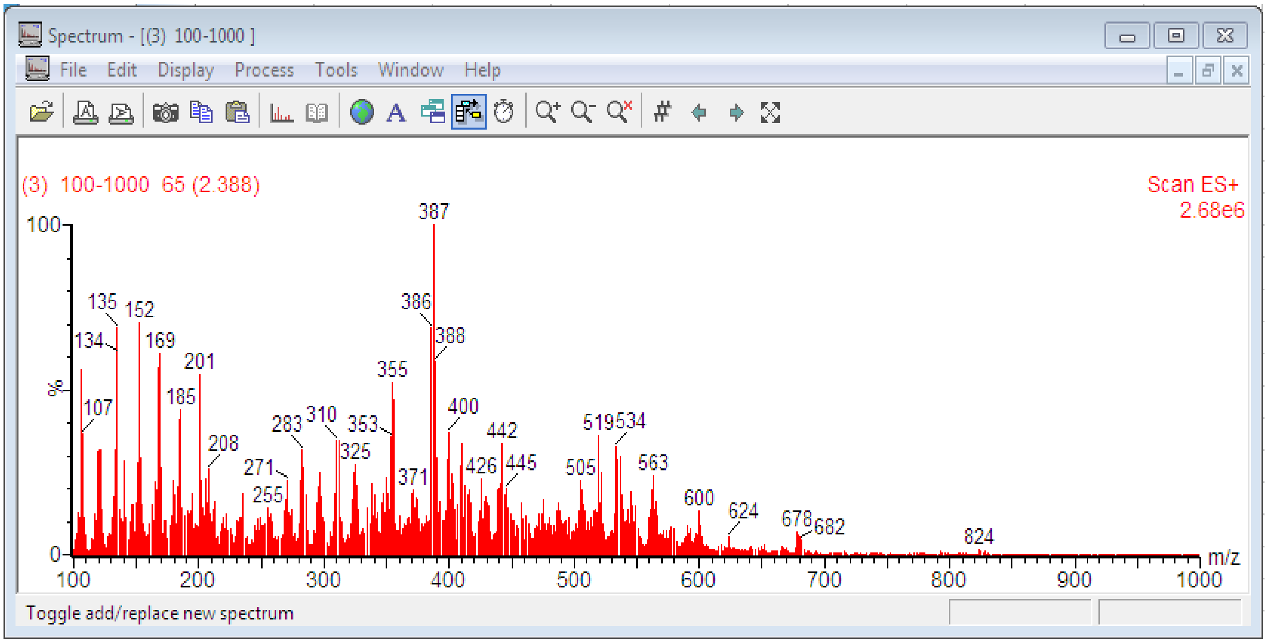

H[1] NMR

N-benzyl-2-{4-[(Z)-[(carbamothioylamino)imino]methyl]-2-methylphenoxy}acetamide(7d)

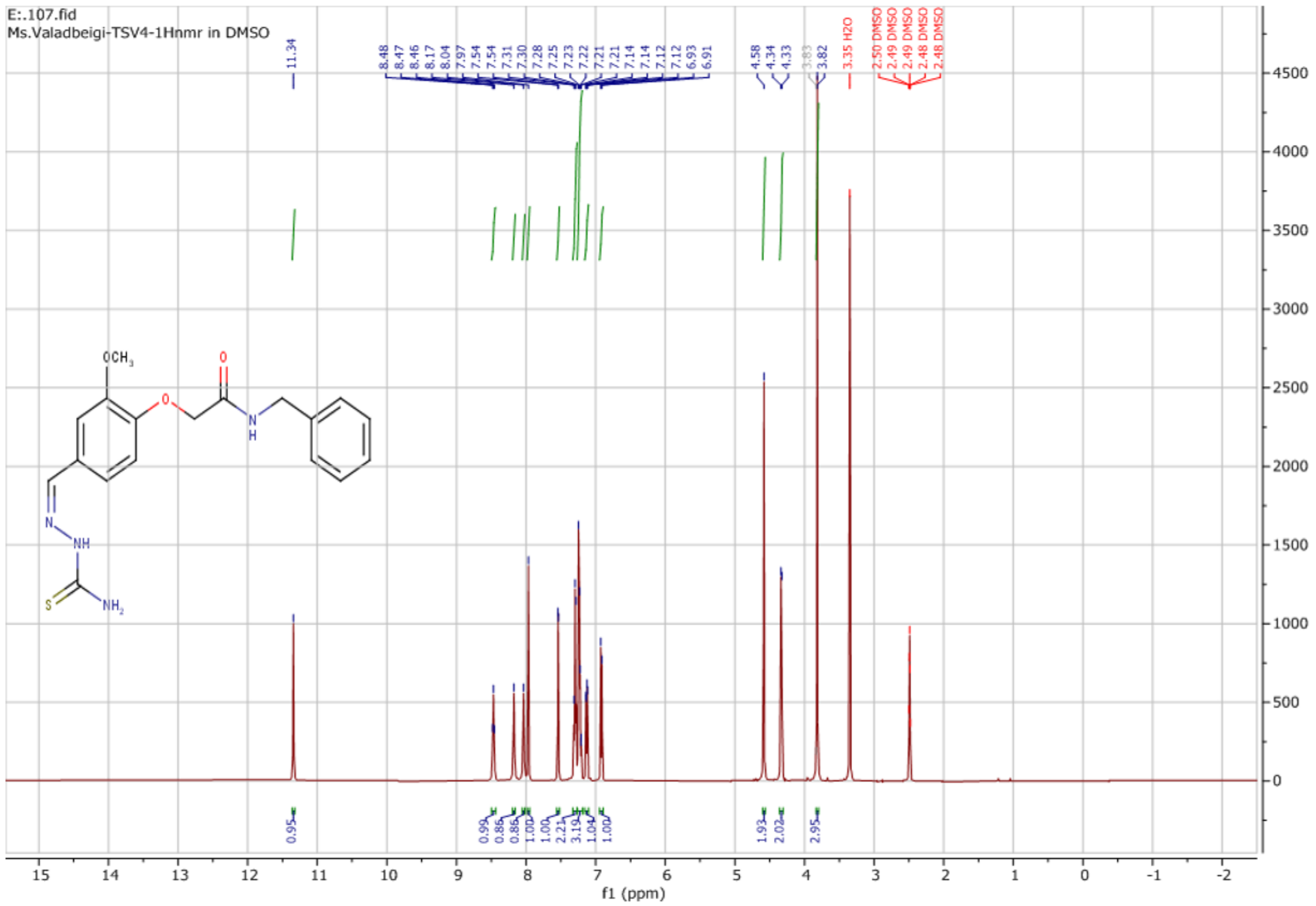

H[1] NMR of 7d

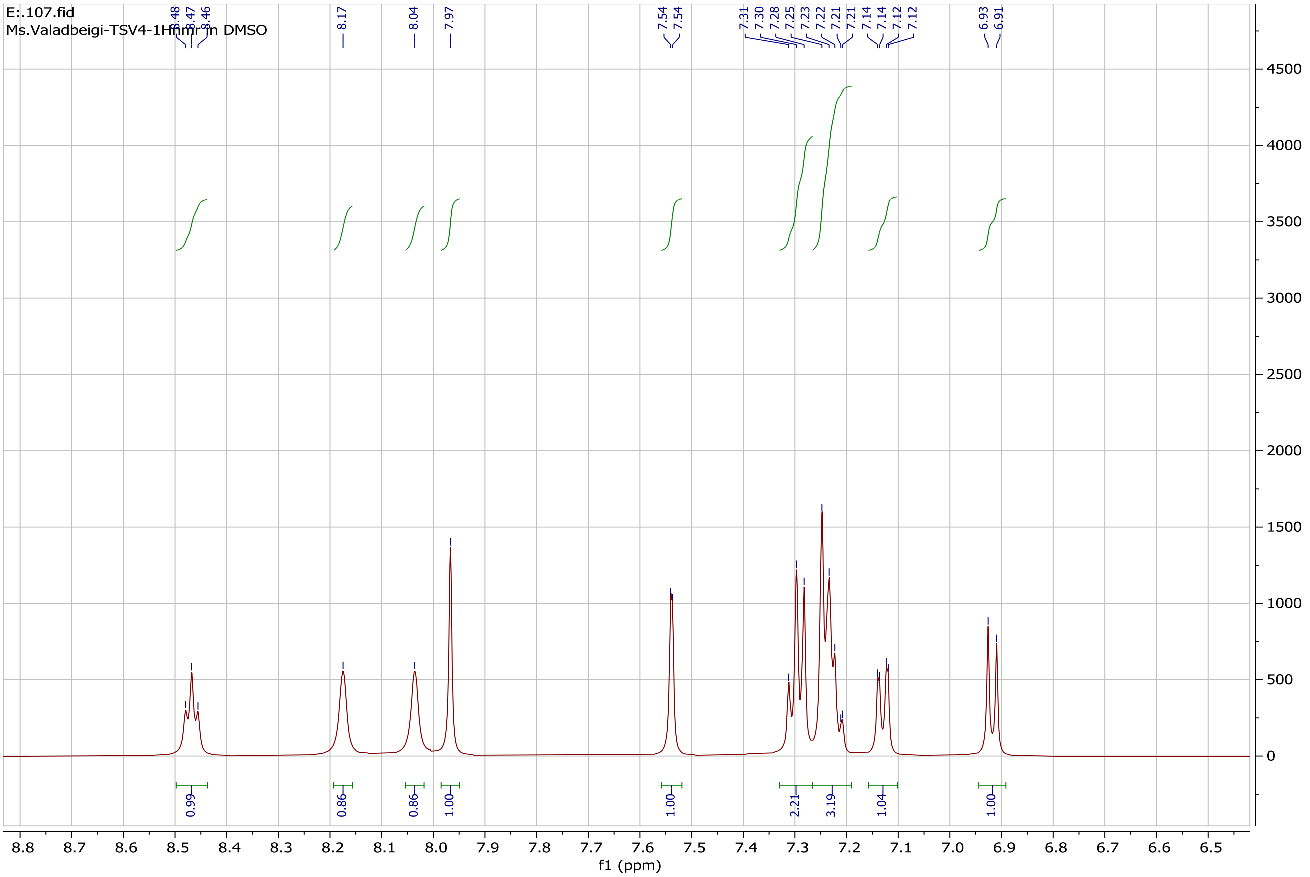

^{13}C NMR of 7d

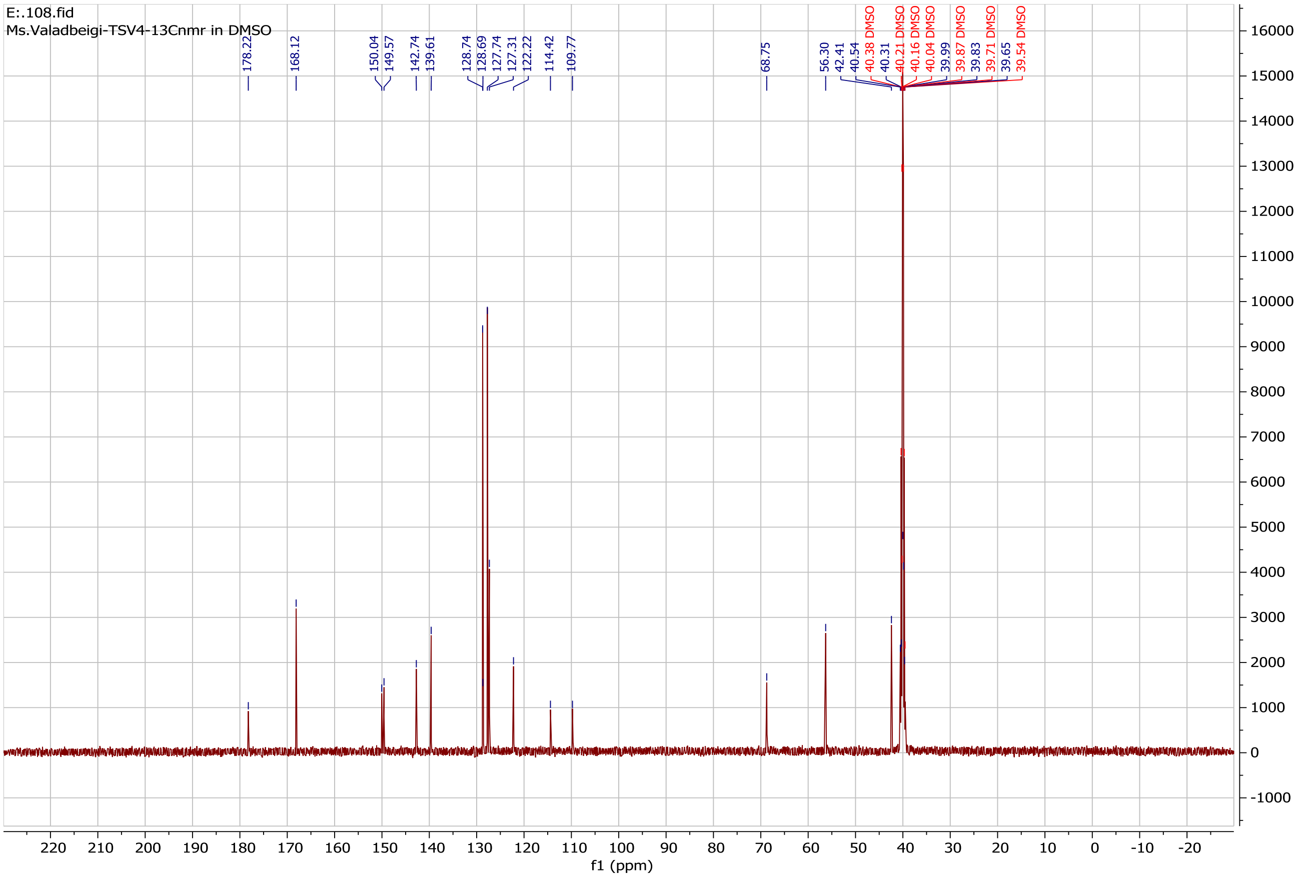

Mass spectra of 7d

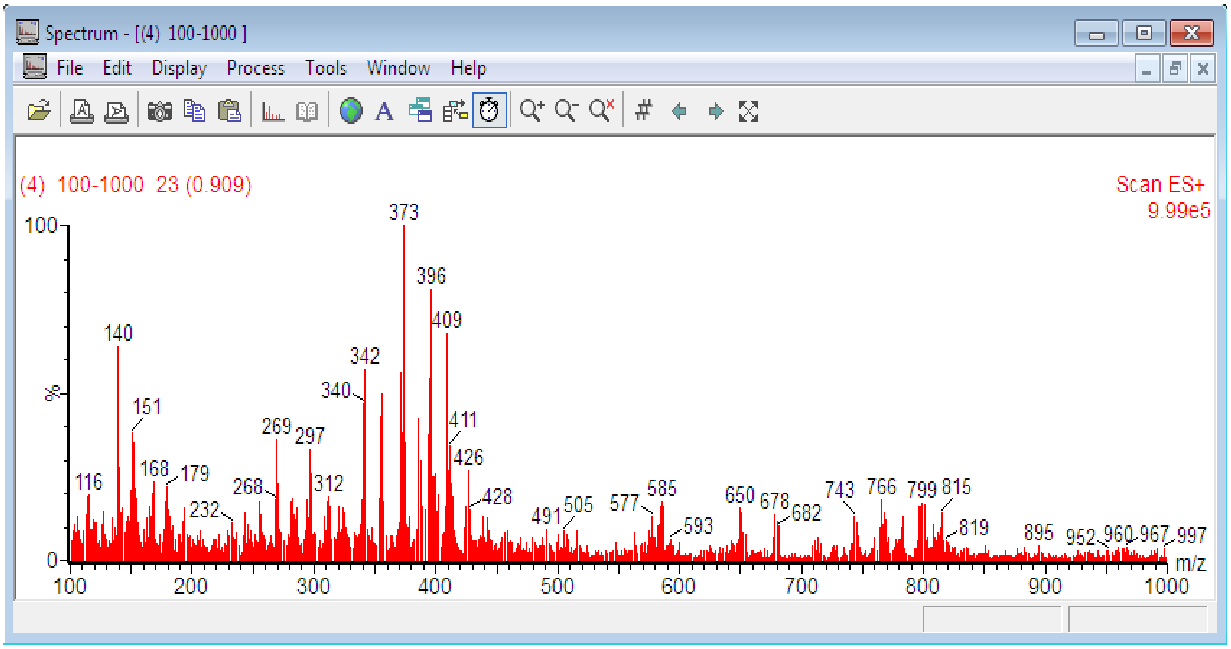

[1] HNMR

2-{4-[(Z)-[(carbamothioylamino)imino]methyl]-2-methoxyphenoxy}-N-(3- methylphenyl) acetamide (7e)

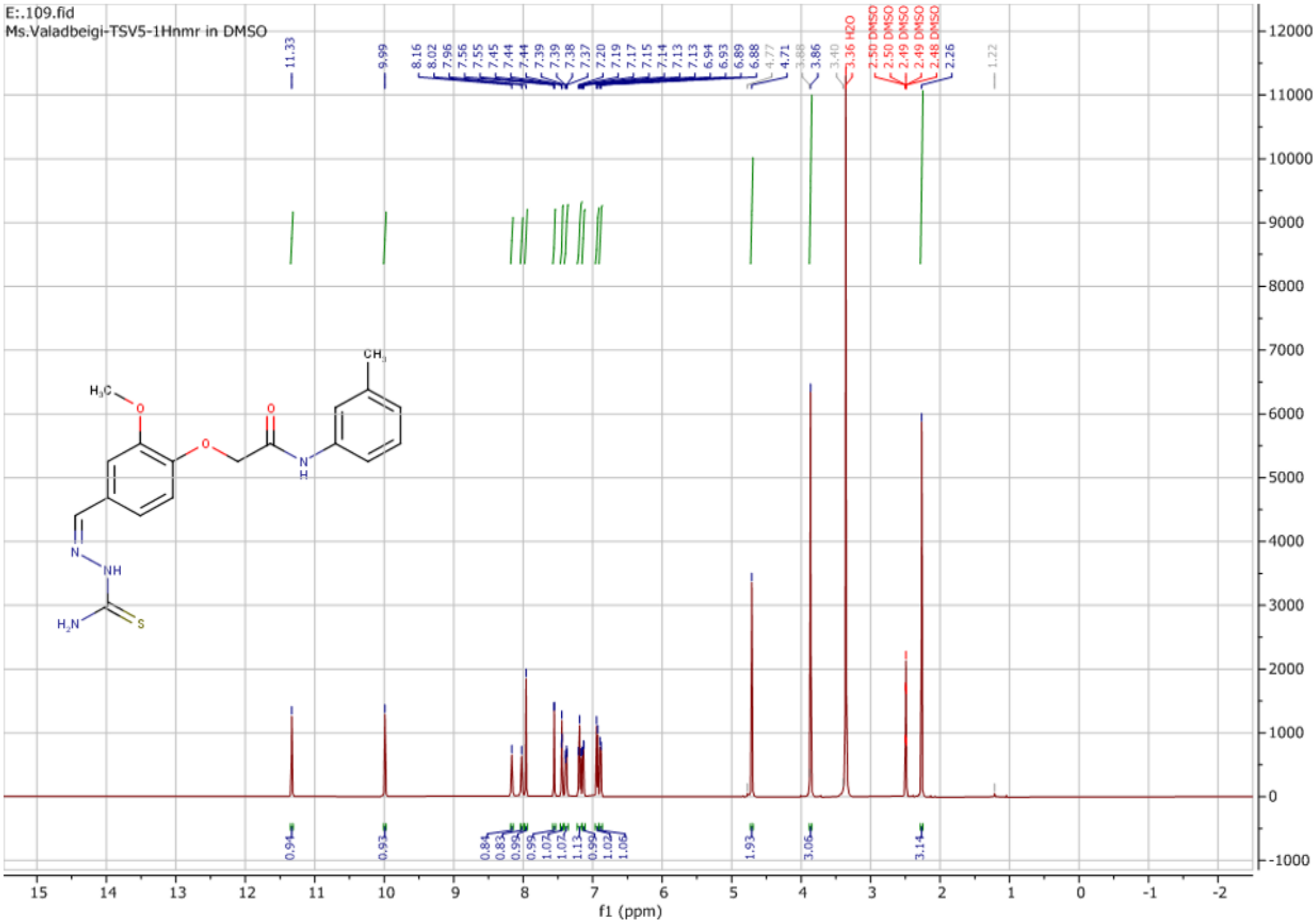

^{1}H NMR of 7e

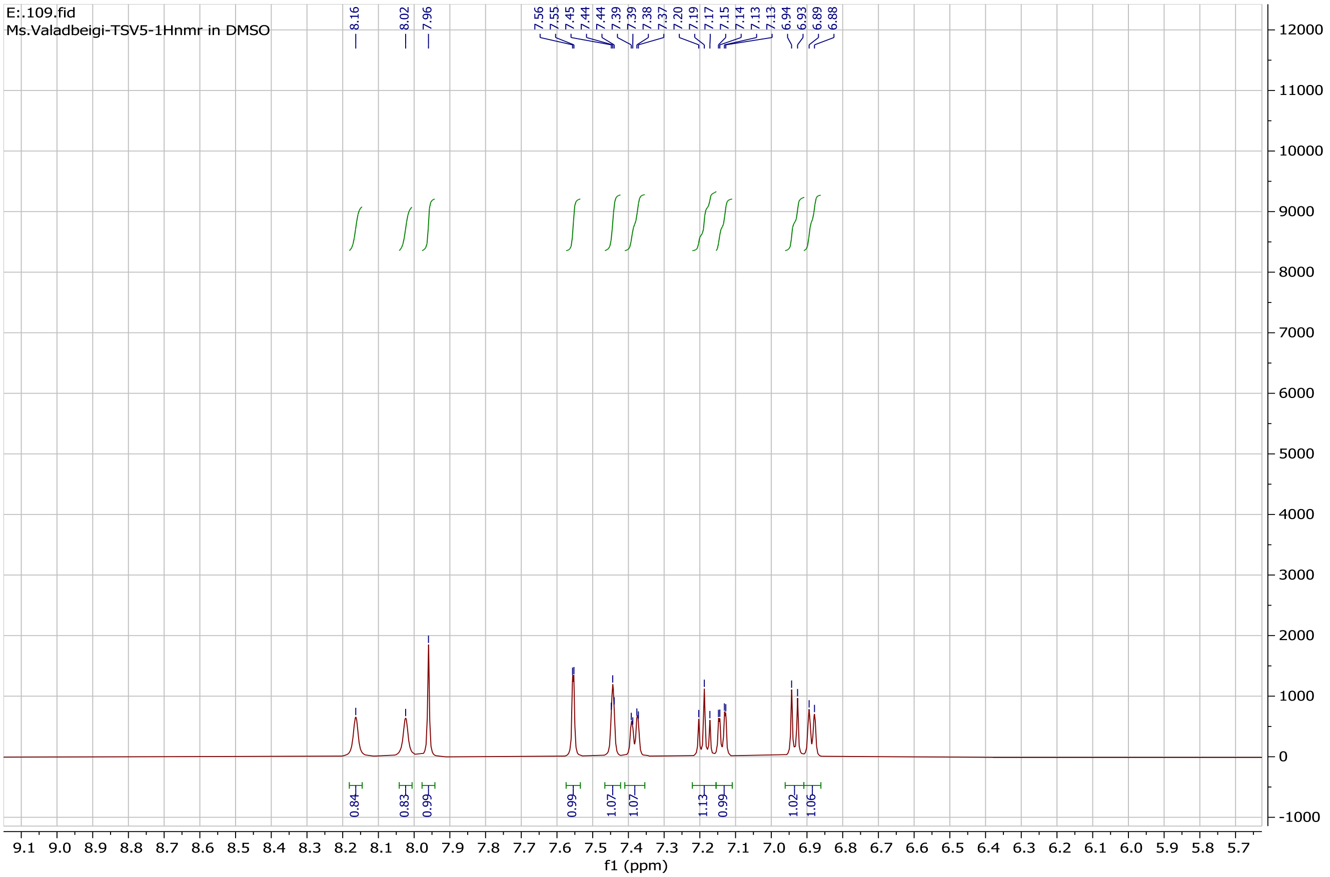

E:.109.fid
Ms.Valadbeigi-TSV5-1Hnmr in DMSO
8.16
8.02
7.96
7.56
7.55
7.45
7.44
7.44
7.39
7.39
7.38
7.37
7.20
7.19
7.17
7.15
7.14
7.13
7.13
6.94
6.93
6.89
6.88
0.84
0.83
0.99
0.99
1.07
1.07
1.13
0.99
1.02
1.06
f1 (ppm)

[13] C NMR of 7e

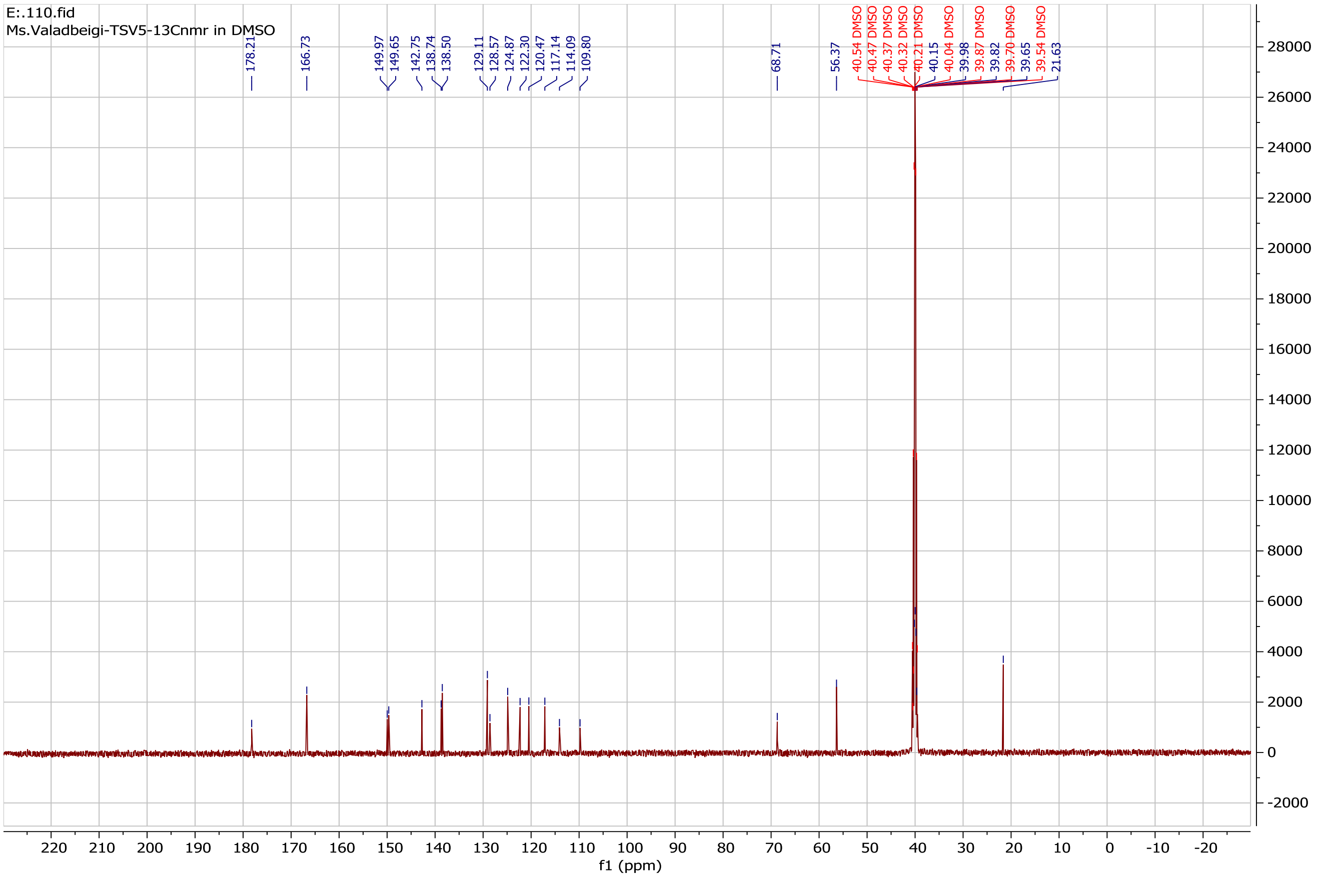

Mass spectra of 7e

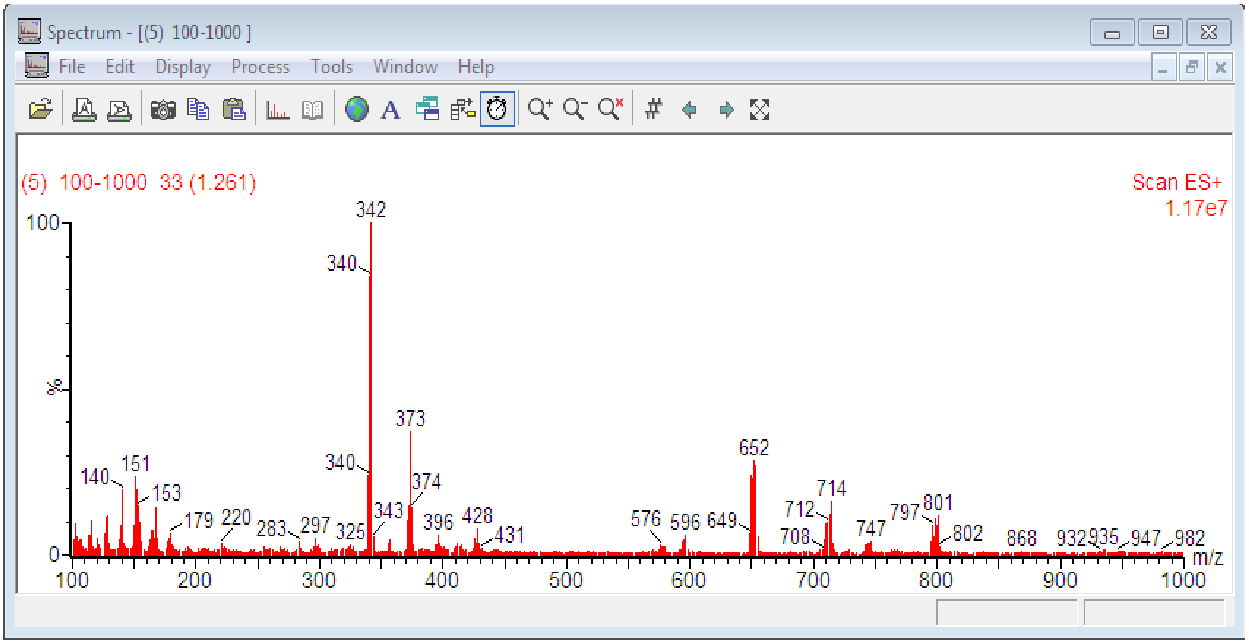

^{1}H NMR

2-{4-[(Z)-[(carbamothioylamino)imino]methyl]-2-methoxyphenoxy}-N-(3- nitrophenyl) acetamide (7f)

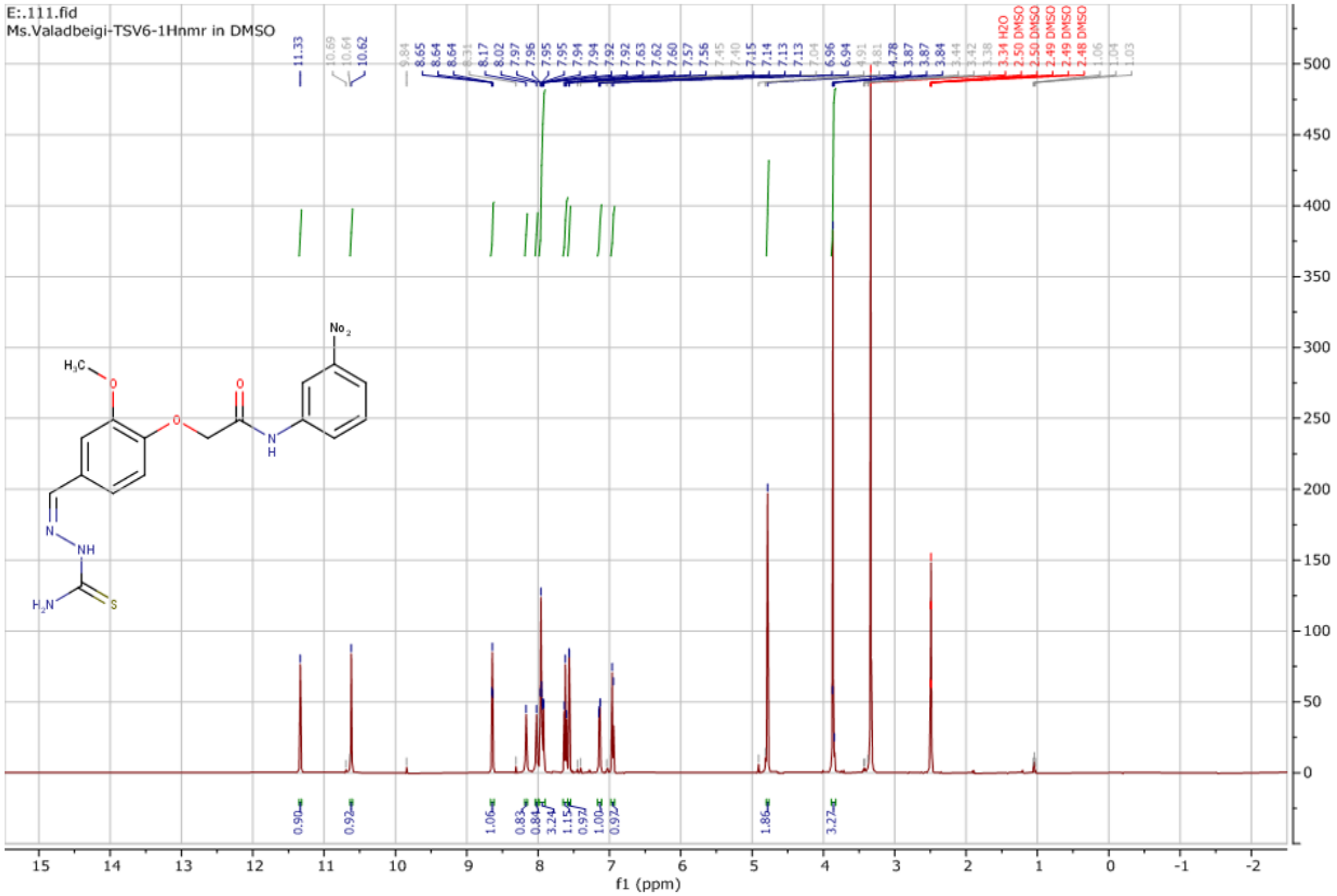

^{1}H NMR of 7f

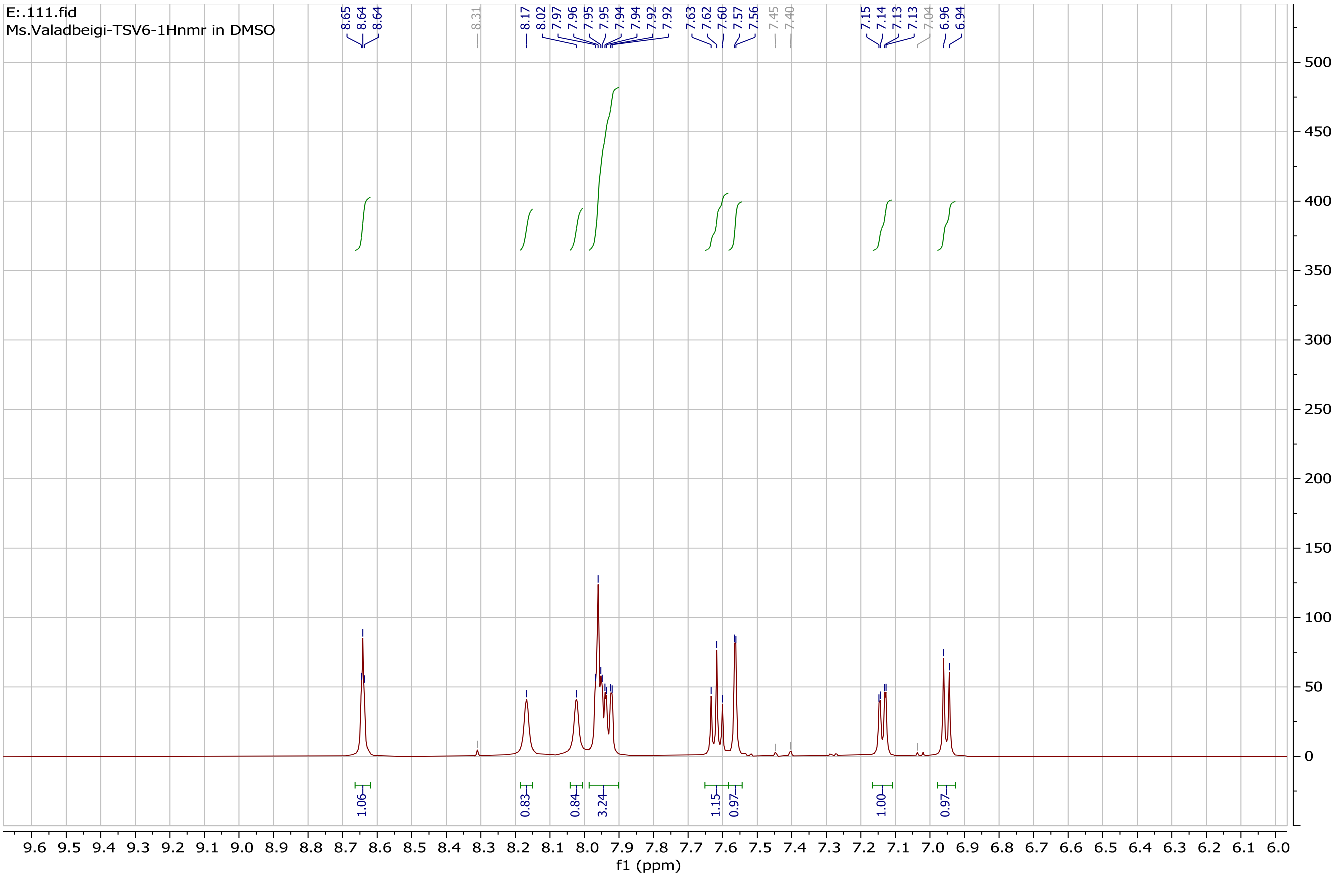

E:.111.fid
Ms.Valadbeigi-TSV6-1Hnmr in DMSO
8.65 8.64 8.64
8.31
8.17 8.02 7.97 7.96 7.95 7.95 7.94 7.94 7.92 7.92
7.63 7.62 7.60 7.57 7.56
7.45 7.40
7.15 7.14 7.13 7.13 7.04 6.96 6.94
1.06 0.83 0.84 3.24 1.15 0.97 1.00 0.97
f1 (ppm)

^{13}C NMR of 7f

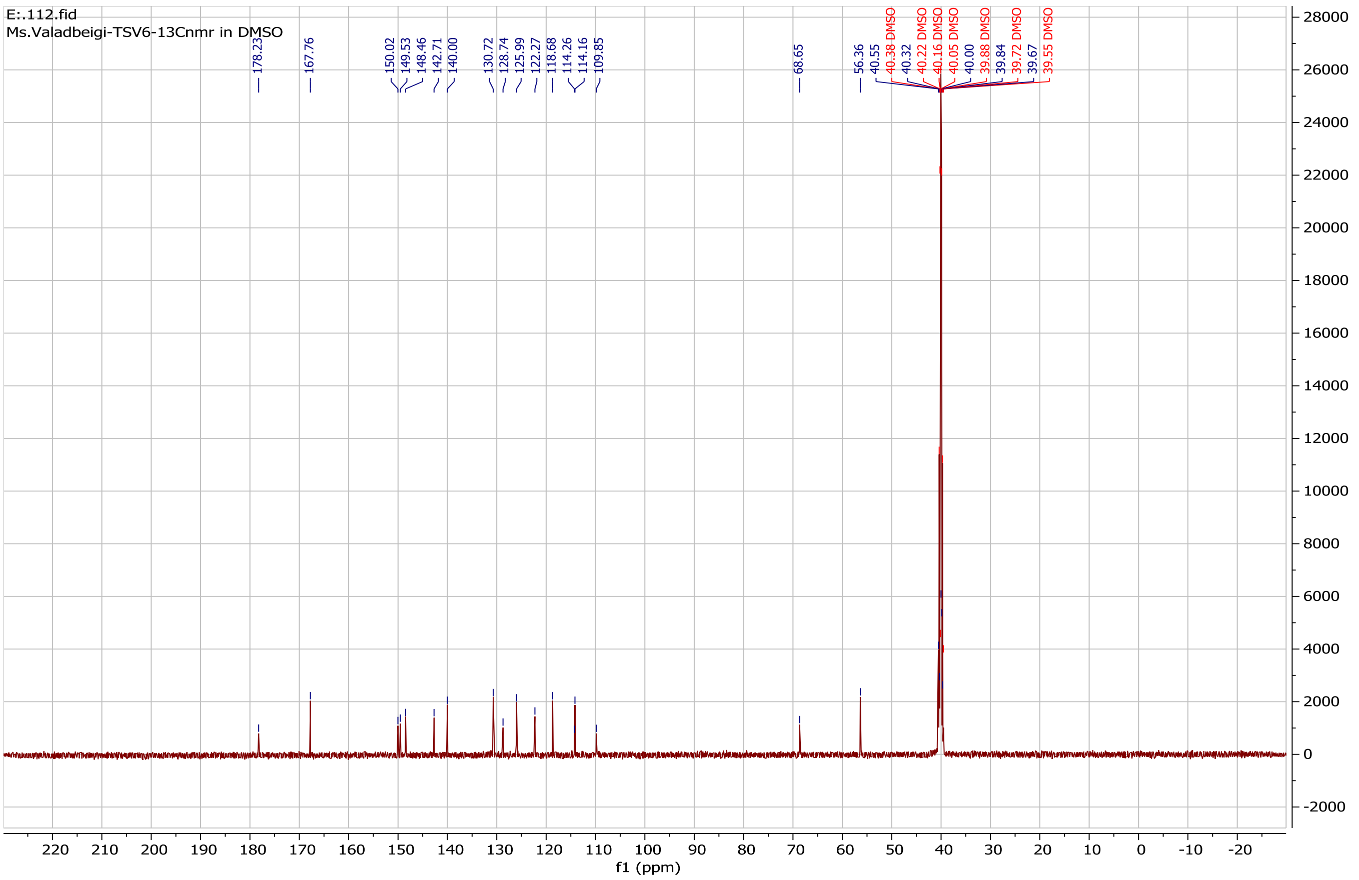

Mass spectra of 7f

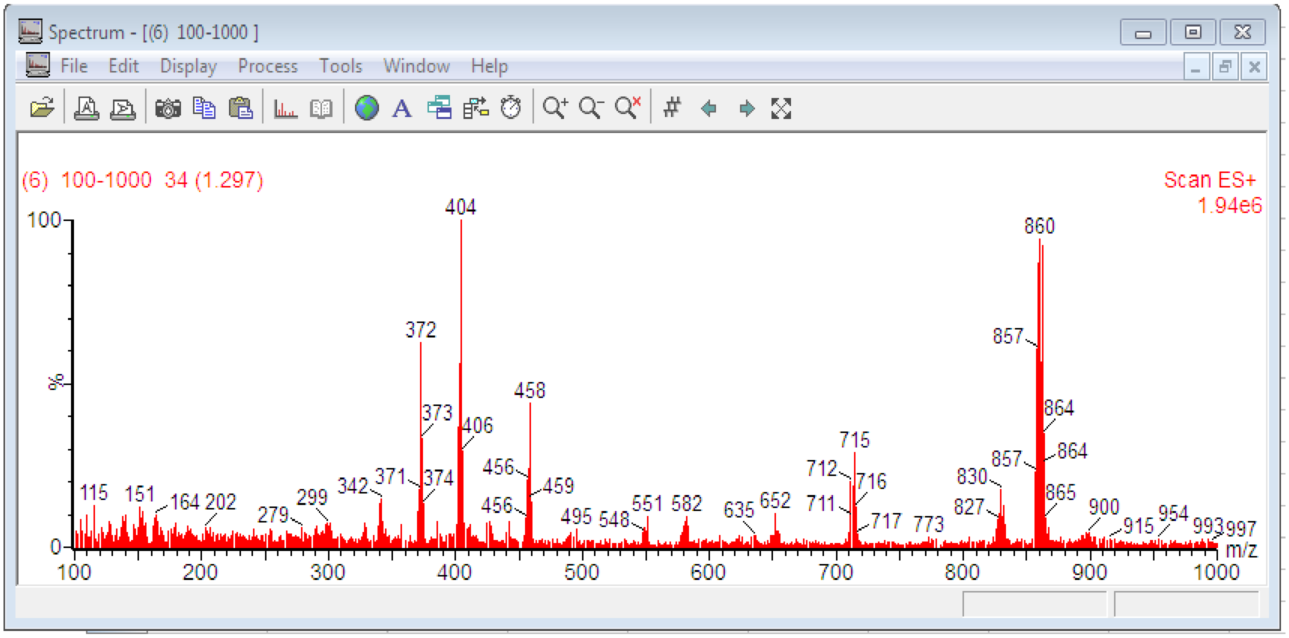

^{1}H NMR

2-{4-[(Z)-[(carbamothioylamino)imino]methyl]-2-methoxyphenoxy}-N-(2-chlorophenyl) acetamid (7g)

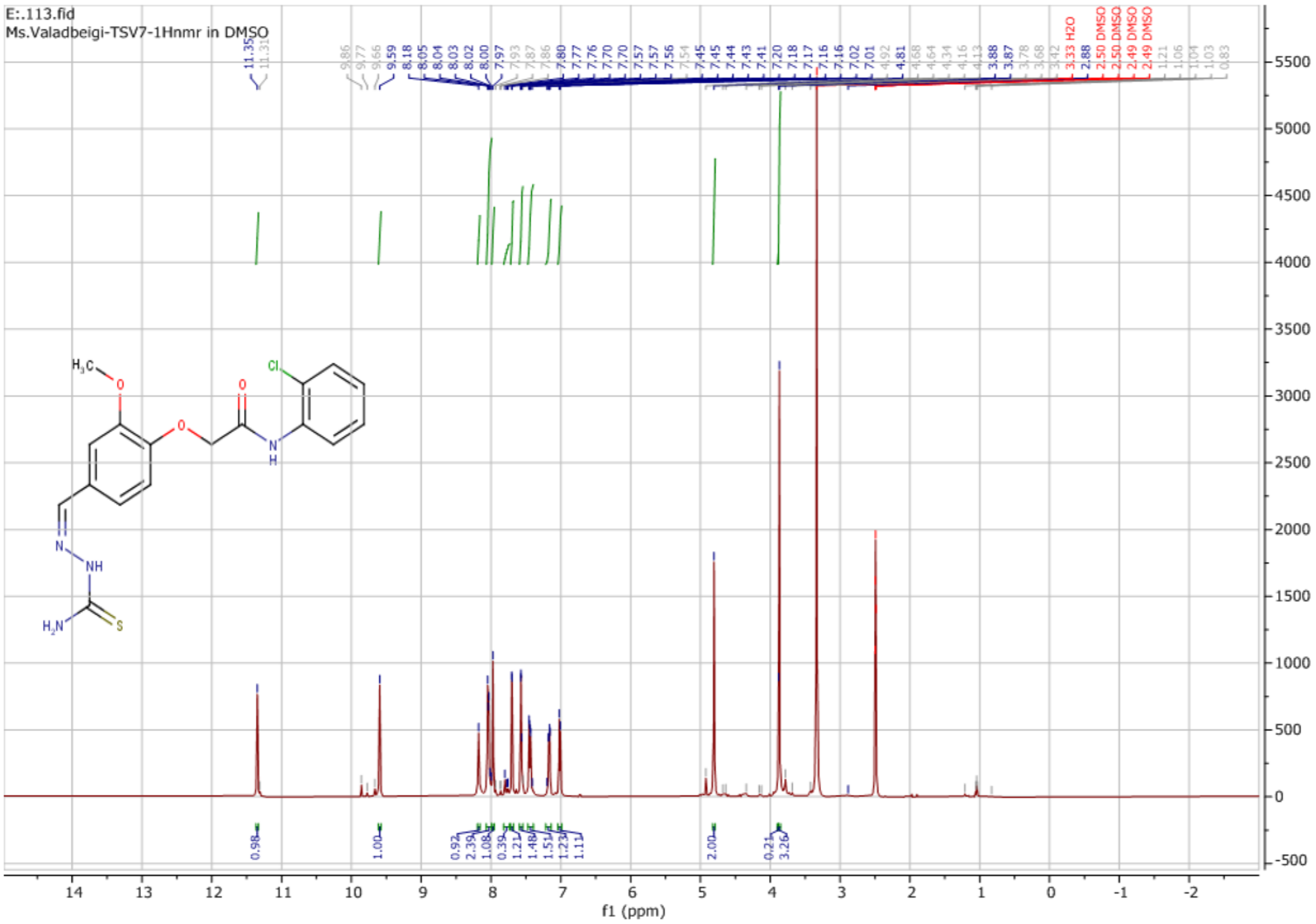

H[1] NMR of 7g

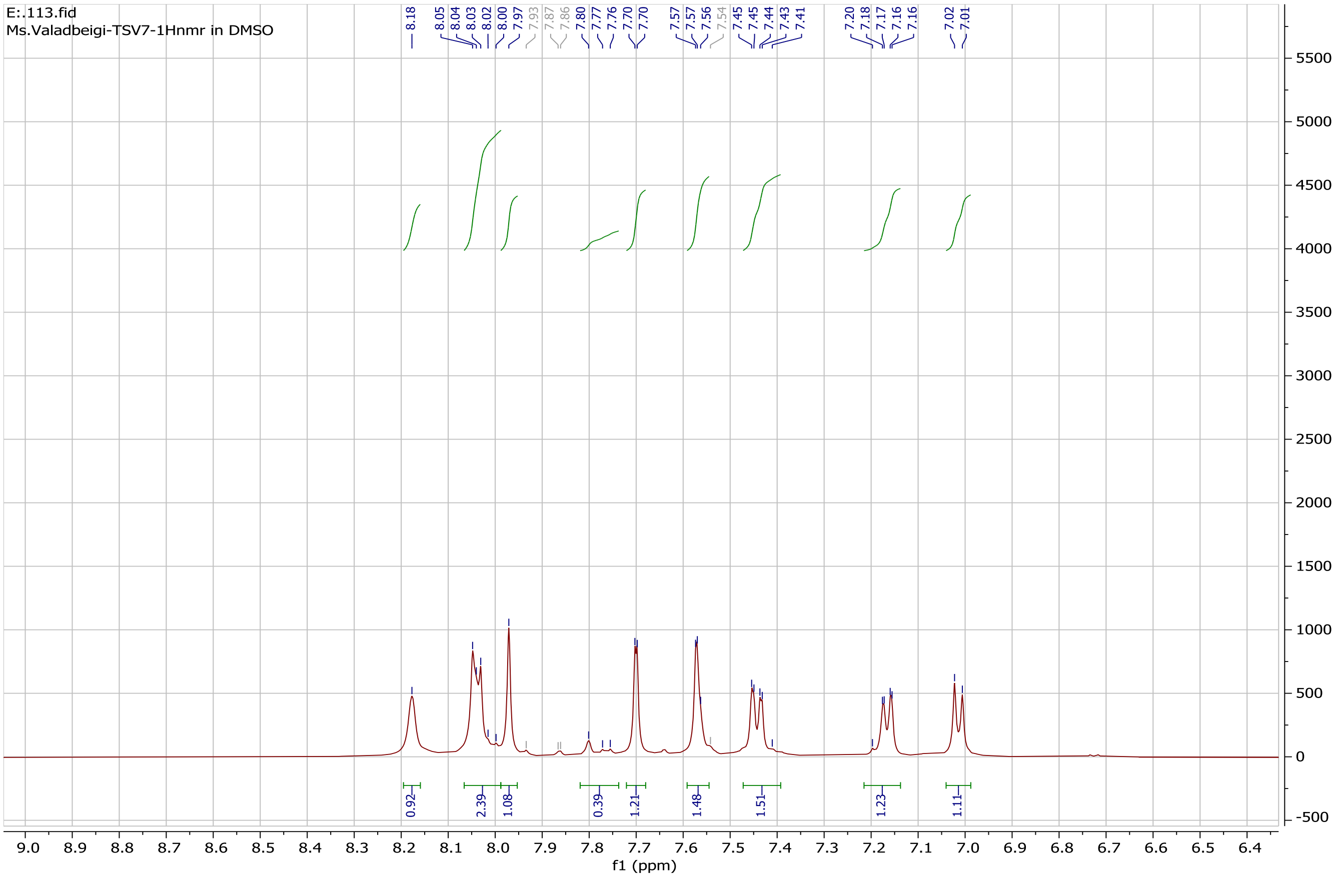
E:.113.fid
Ms.Valadbeigi-TSV7-1Hnmr in DMSO
8.18
8.05
8.04
8.03
8.02
8.00
7.97
7.93
7.87
7.86
7.80
7.77
7.76
7.70
7.70
7.57
7.57
7.56
7.54
7.45
7.45
7.44
7.43
7.41
7.20
7.18
7.17
7.16
7.16
7.02
7.01
0.92
2.39
1.08
0.39
1.21
1.48
1.51
1.23
1.11
f1 (ppm)

[13] C NMR of 7g

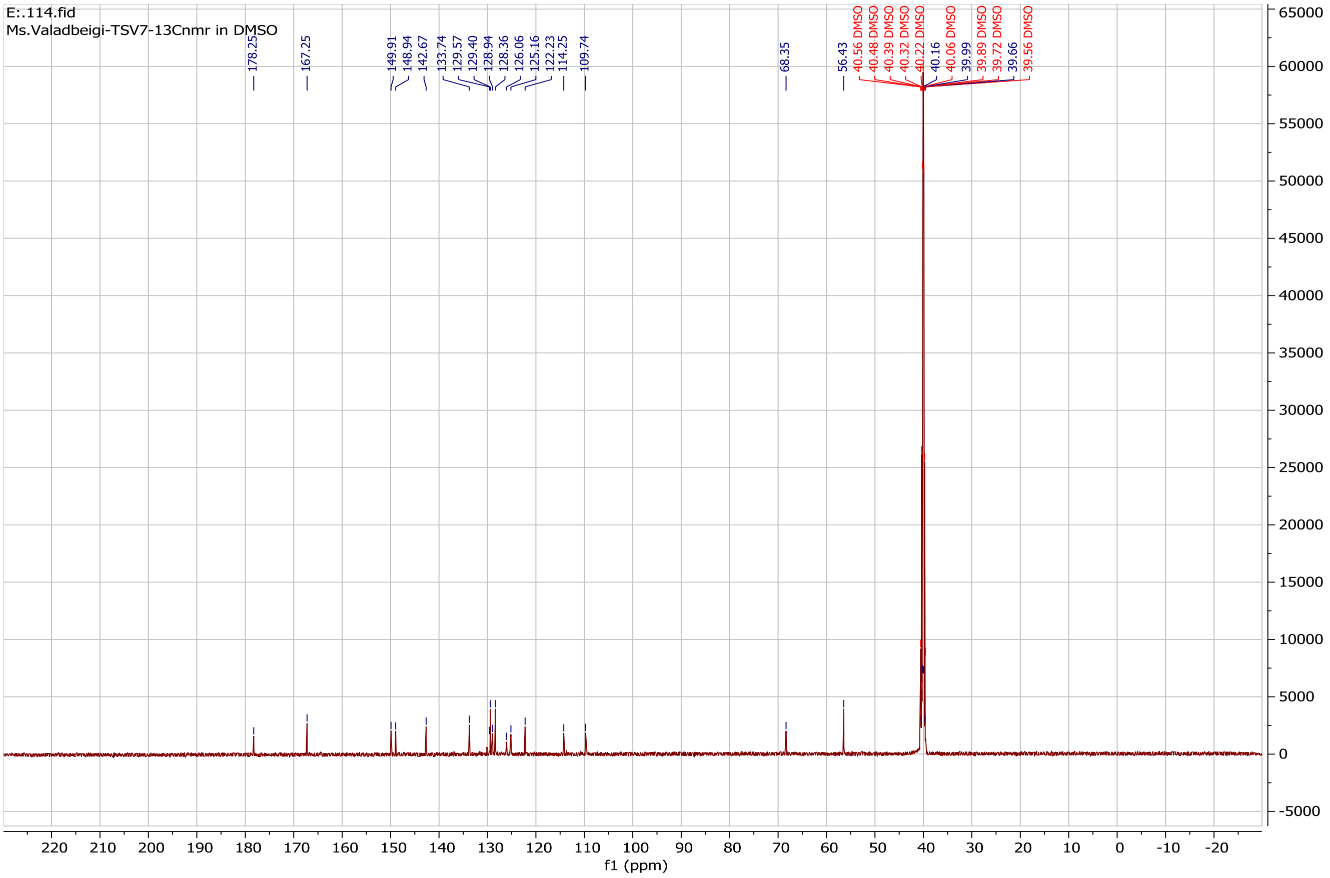

Mass spectra of 7g

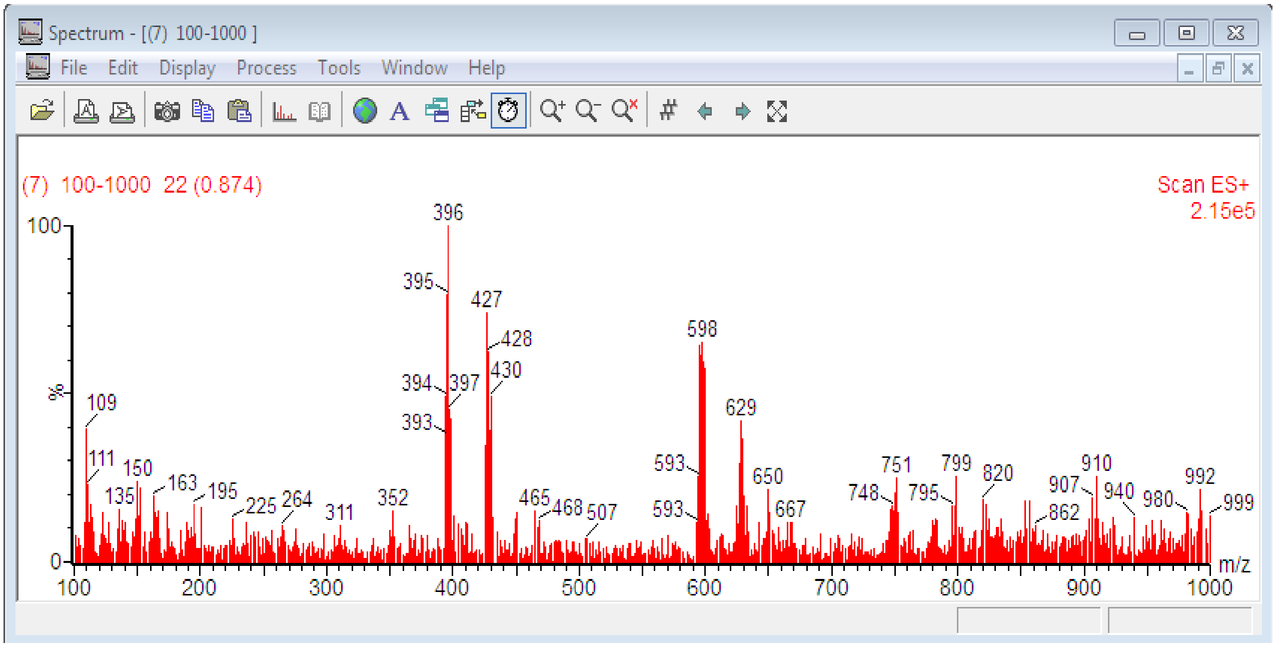

^{1}H NMR

N-(4-bromophenyl)-2-{4-[(Z)-[(carbamothioylamino)imino]methyl]-2- methoxyphenoxy} acetamide (7h)

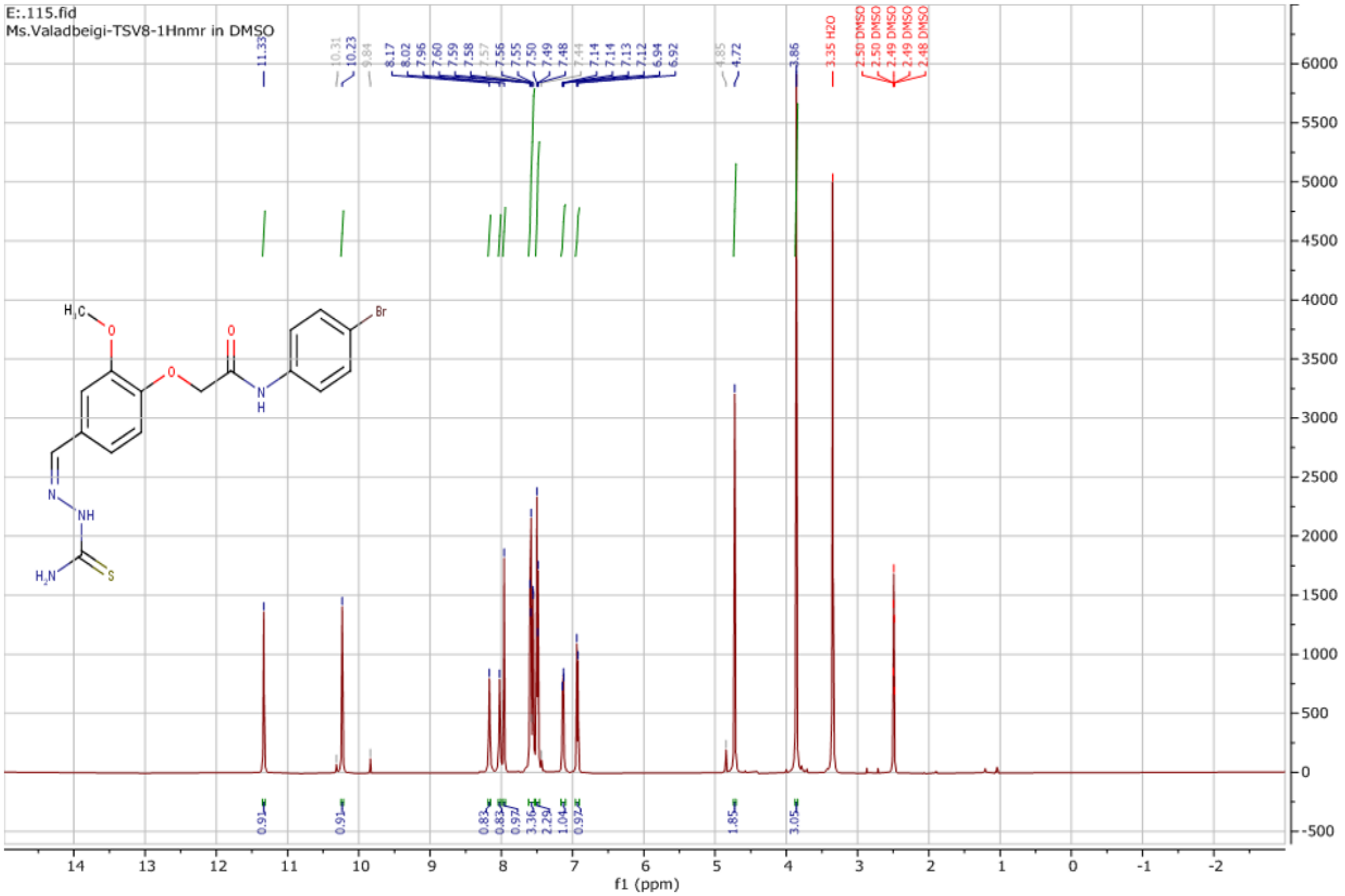

[1] HNMR of 7h

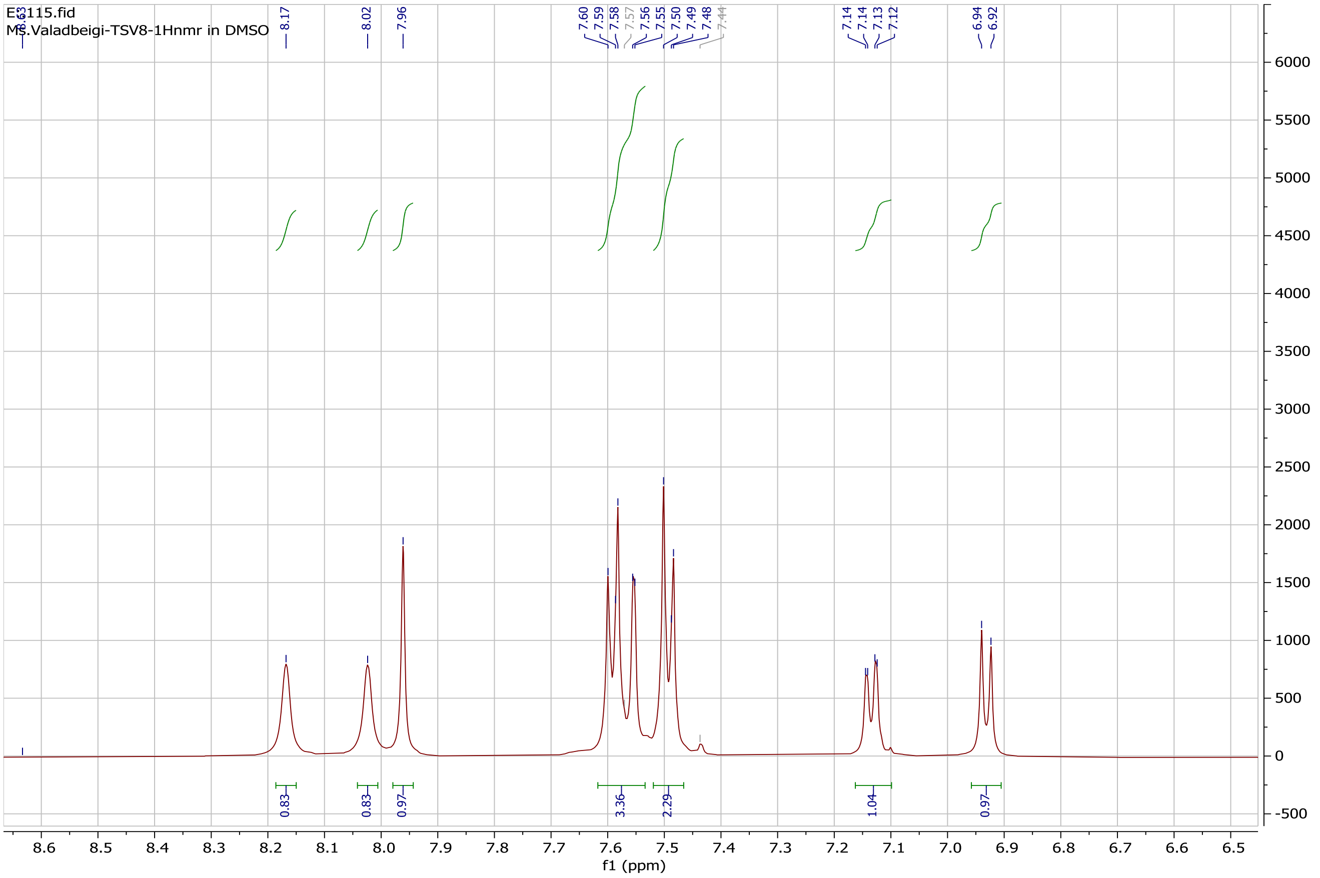
E3115.fid
MS.Valadbeigi-TSV8-1Hnmr in DMSO
8.63
8.17
8.02
7.96
7.60
7.59
7.58
7.57
7.56
7.55
7.50
7.49
7.48
7.44
7.14
7.14
7.13
7.12
6.94
6.92
0.83
0.83
0.97
3.36
2.29
1.04
0.97
f1 (ppm)

^{13}C NMR of 7h

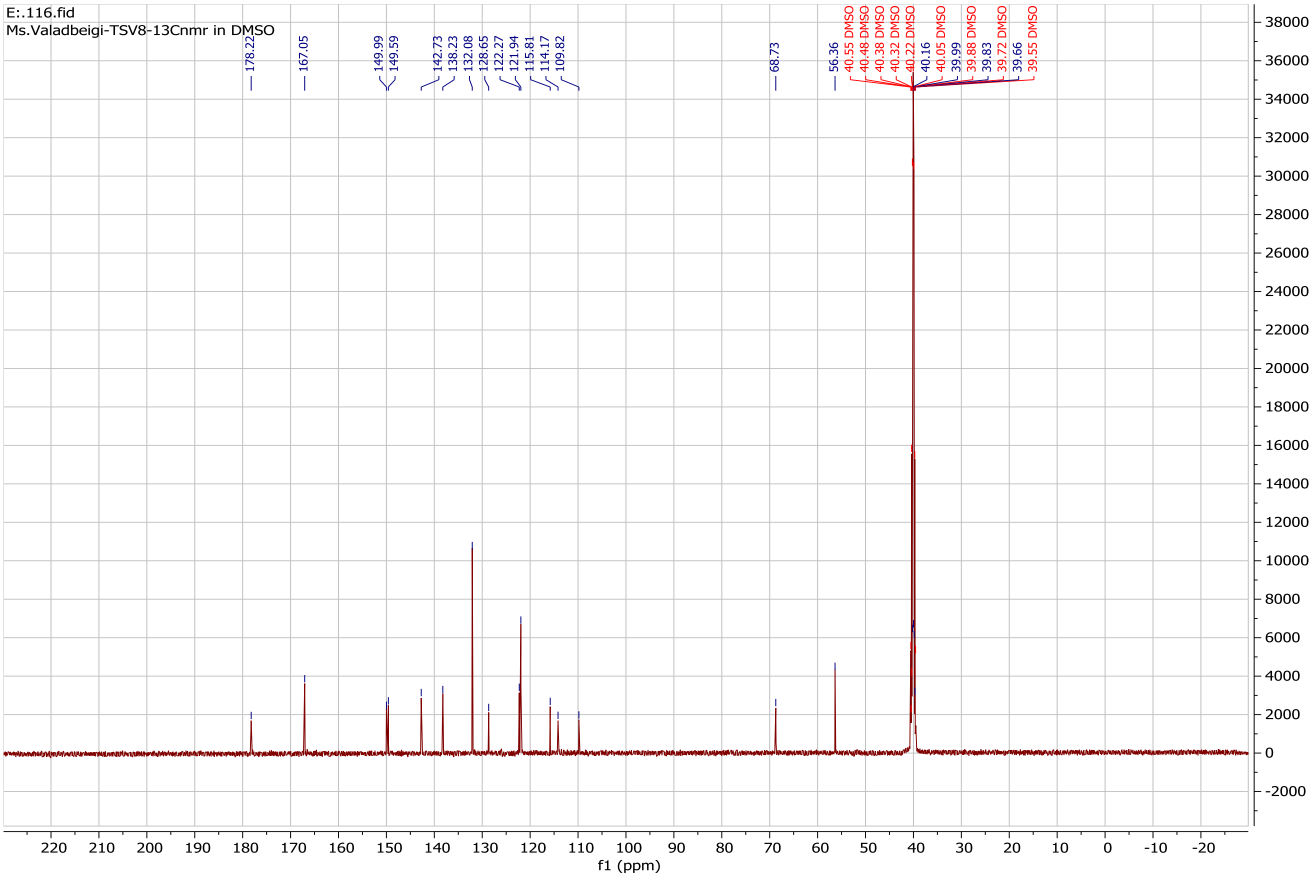

Mass spectra of 7h

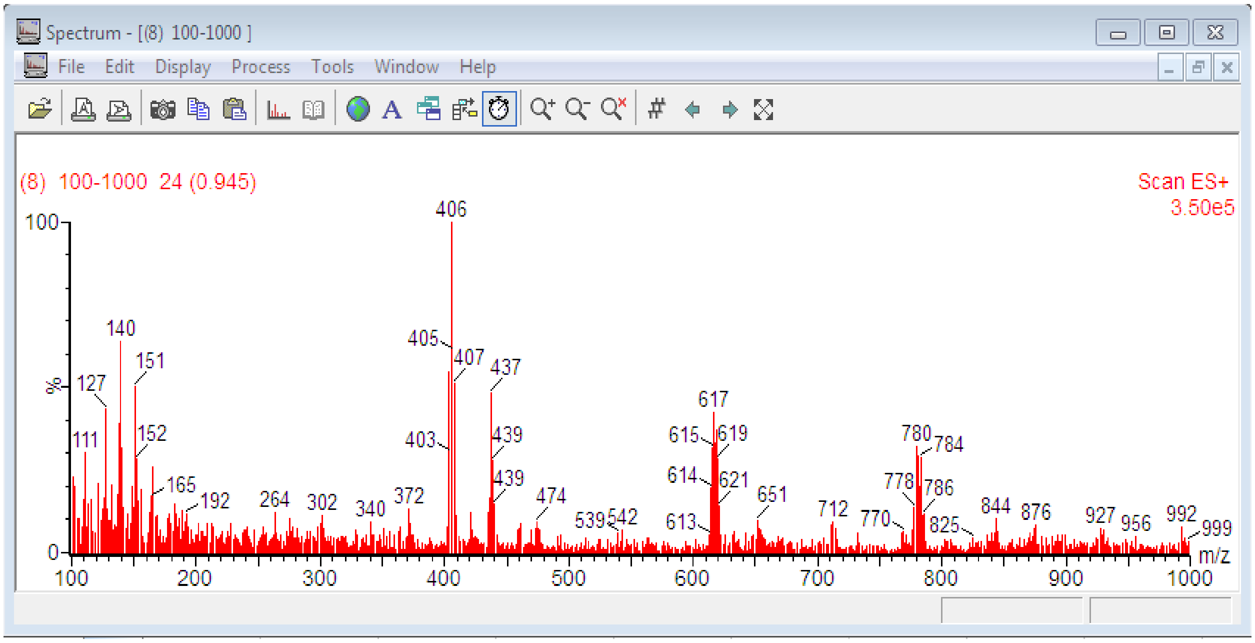

^{1}H NMR

2-{4-[(Z)-[(carbamothioylamino)imino]methyl]-2-methoxyphenoxy}-N-(4- methylphenyl) acetamide (7i)

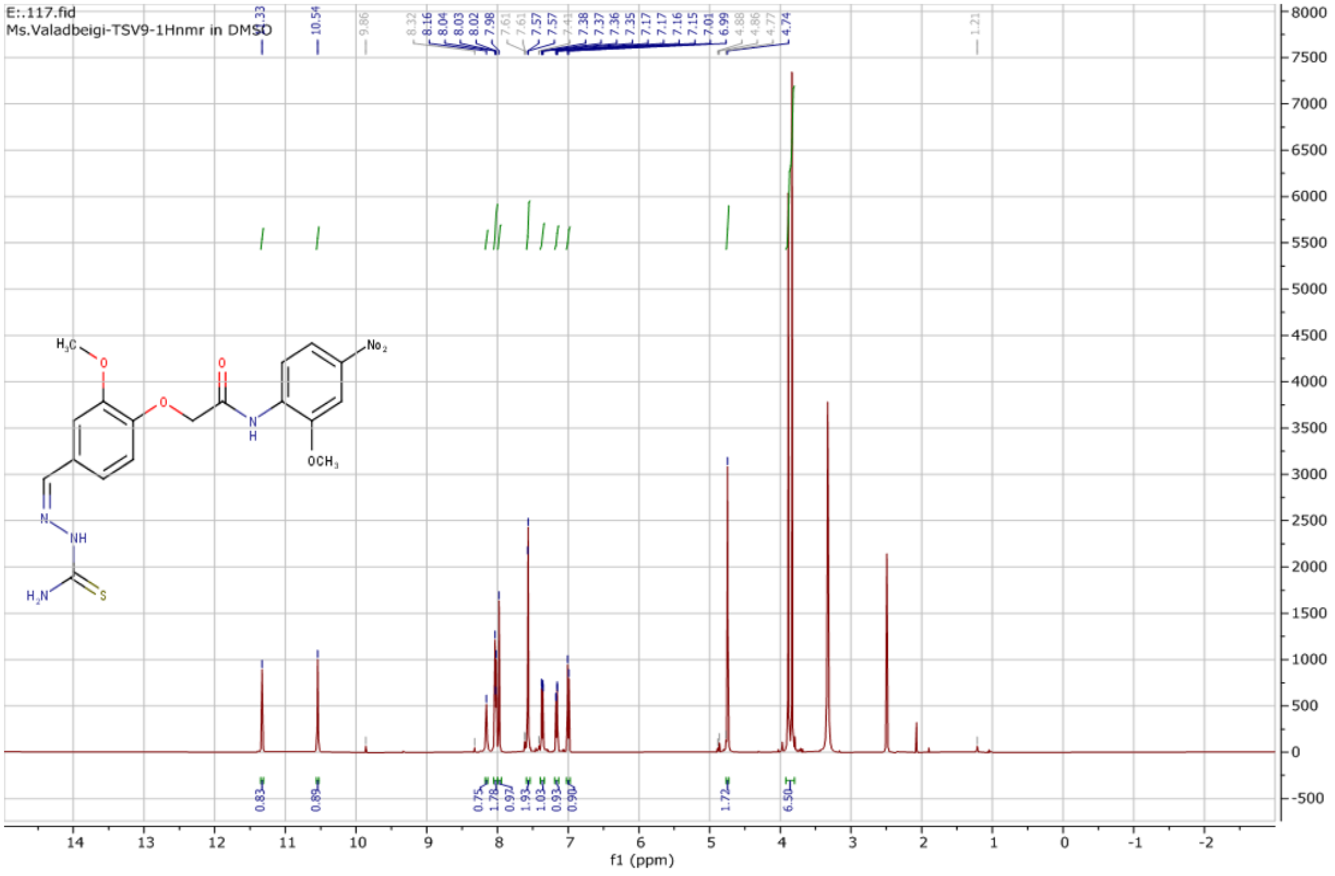

^{1}H NMR of 7i

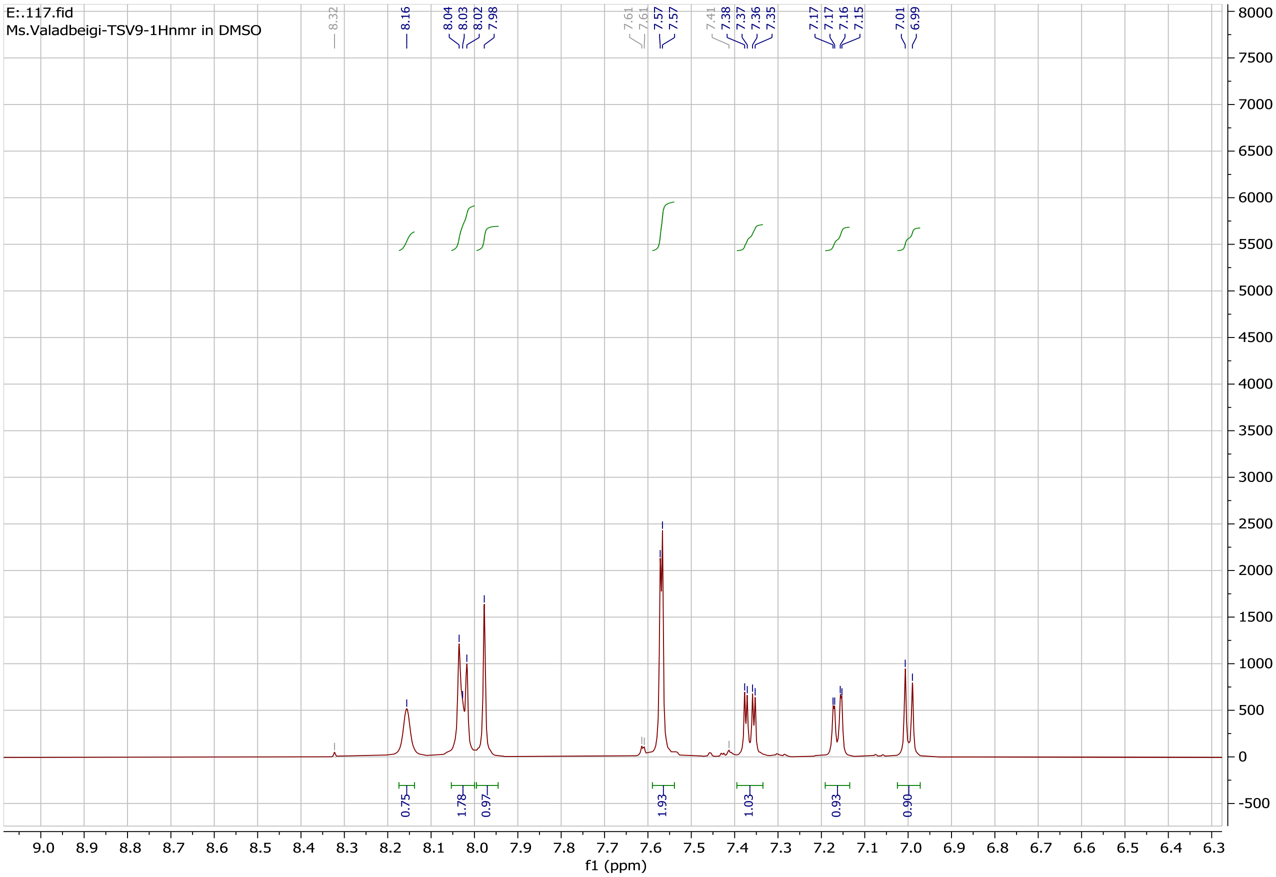

E:.117.fid
Ms.Valadbeigi-TSV9-1Hnmr in DMSO
8.32
8.16
8.04
8.03
8.02
7.98
7.61
7.61
7.57
7.57
7.41
7.38
7.37
7.36
7.35
7.17
7.17
7.16
7.15
7.01
6.99
0.75
1.78
0.97
1.93
1.03
0.93
0.90
f1 (ppm)

^{13}C NMR of 7i

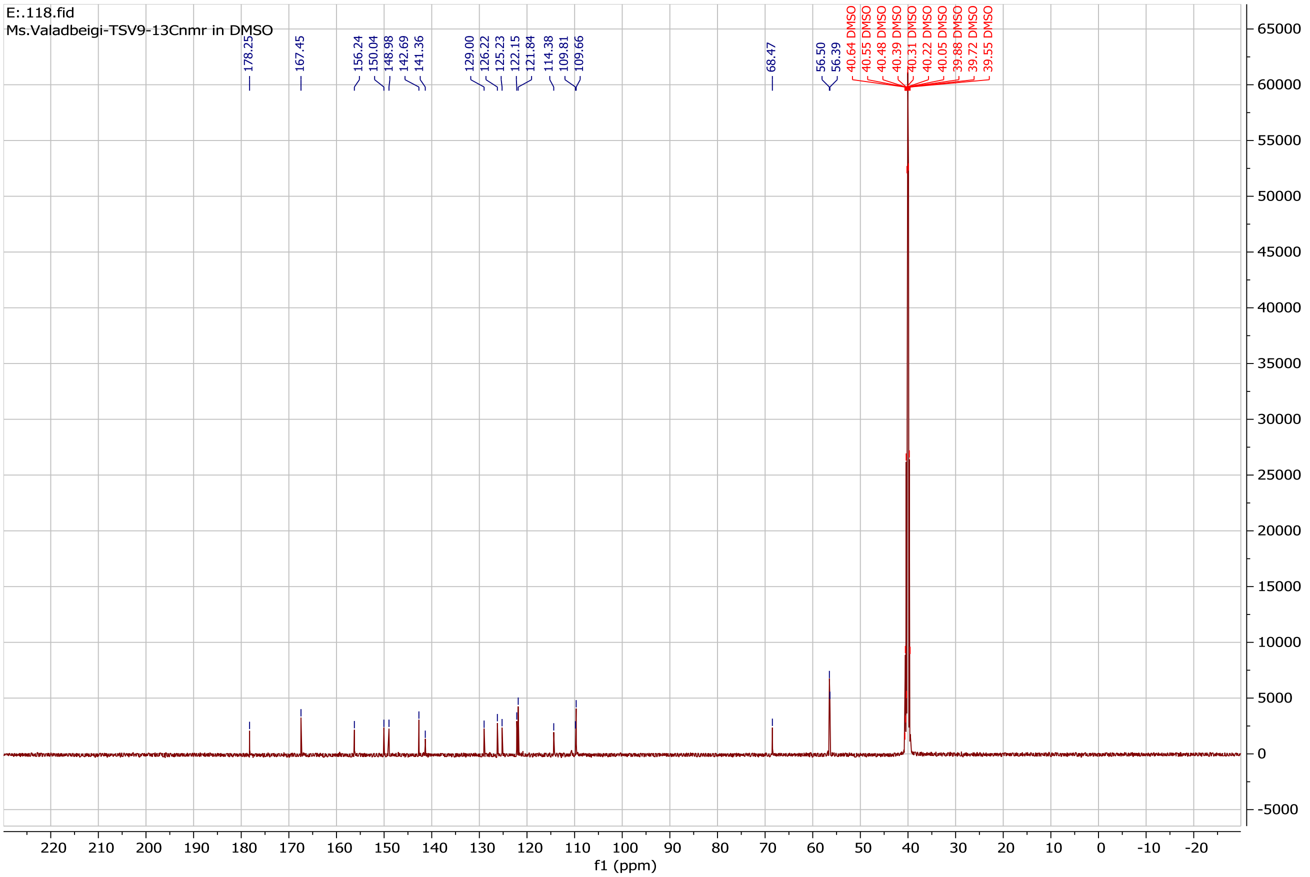

Mass spectra of 7i

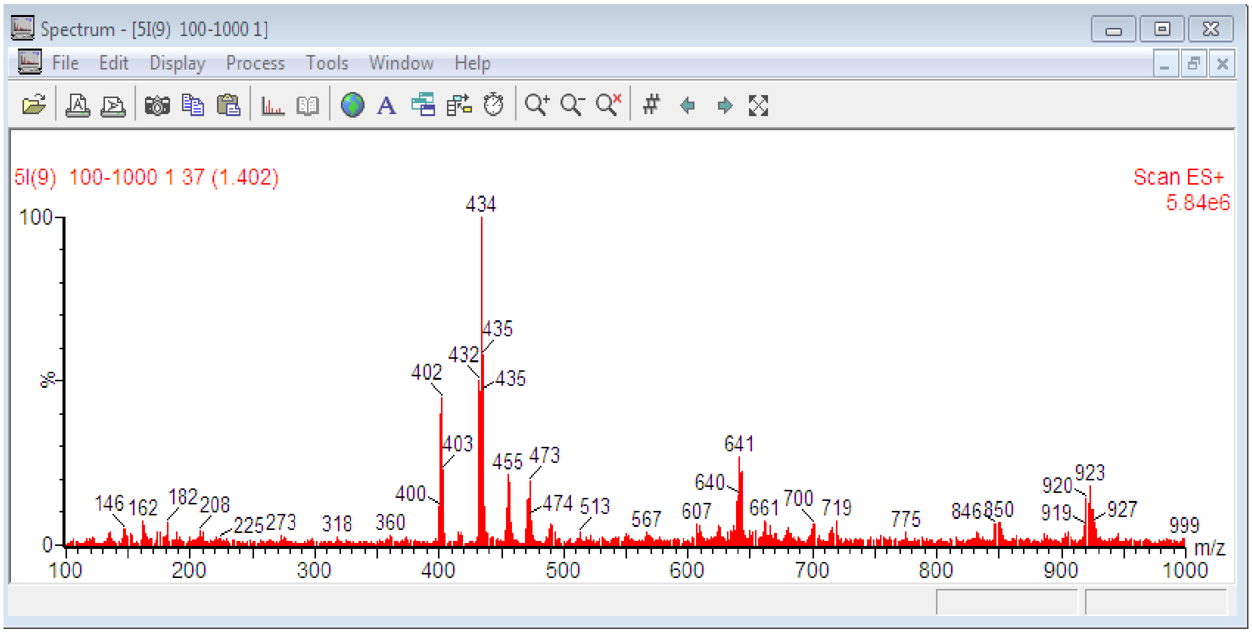

[1] HNMR

2-{4-[(Z)-[(carbamothioylamino)imino]methyl]-2-methoxyphenoxy}-N-(2,3-dimethylphenyl)acetamide (7j)

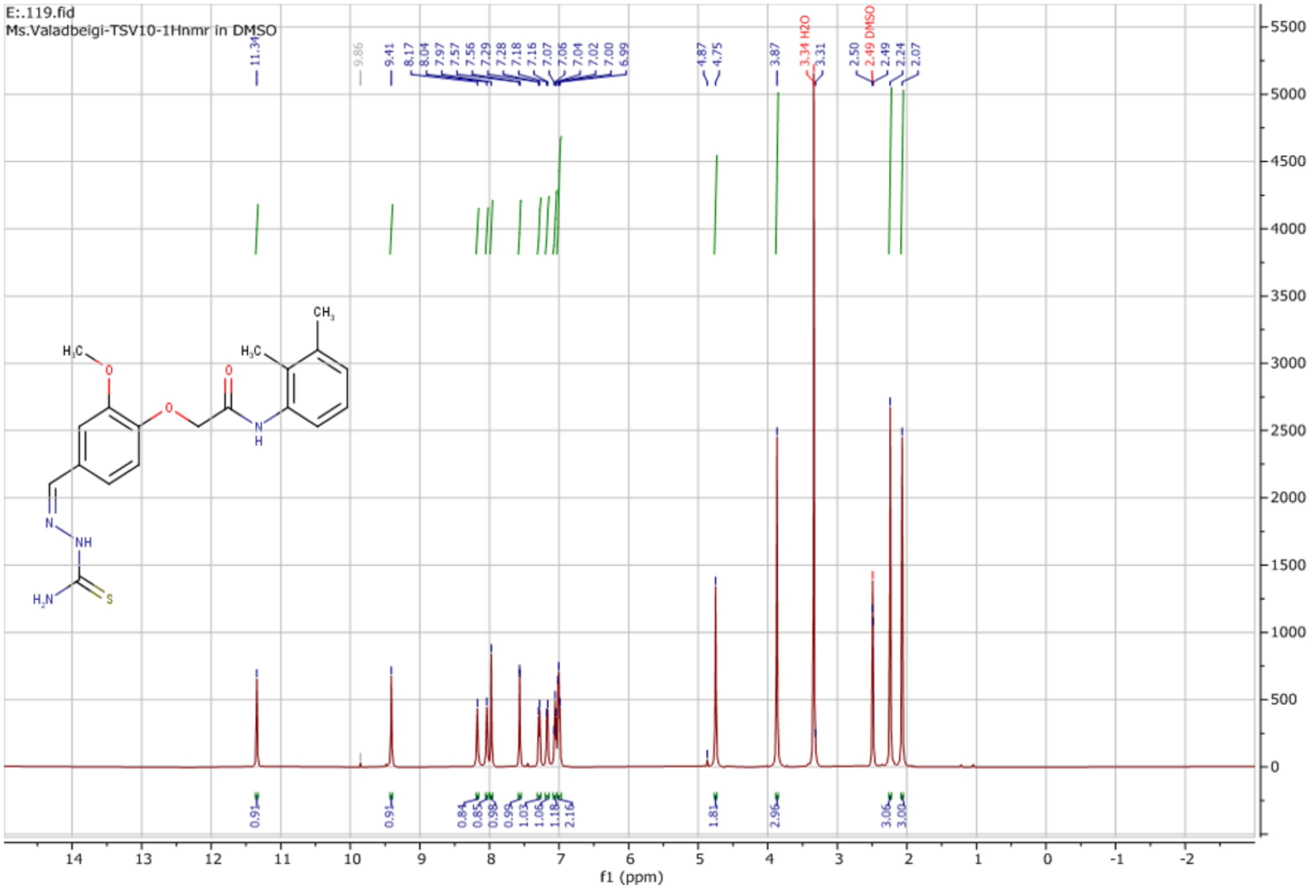

H^1 NMR of 7j

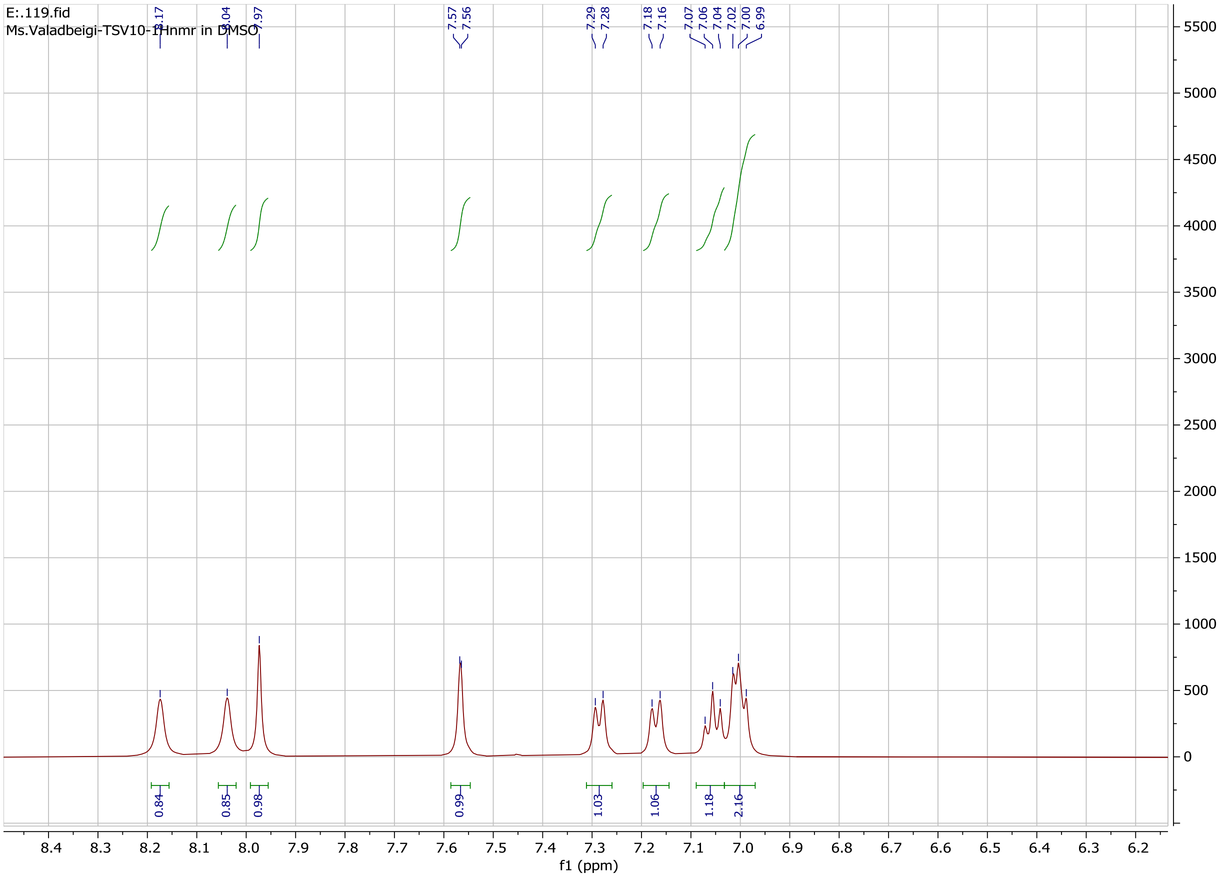

E:.119.fid
Ms.Valadbeigi-TSV10-1Hnmr in DMSO
8.17
8.04
7.97
7.57
7.56
7.29
7.28
7.18
7.16
7.07
7.06
7.04
7.02
7.00
6.99
0.84
0.85
0.98
0.99
1.03
1.06
1.18
2.16
f1 (ppm)

^{13}C NMR of 7j

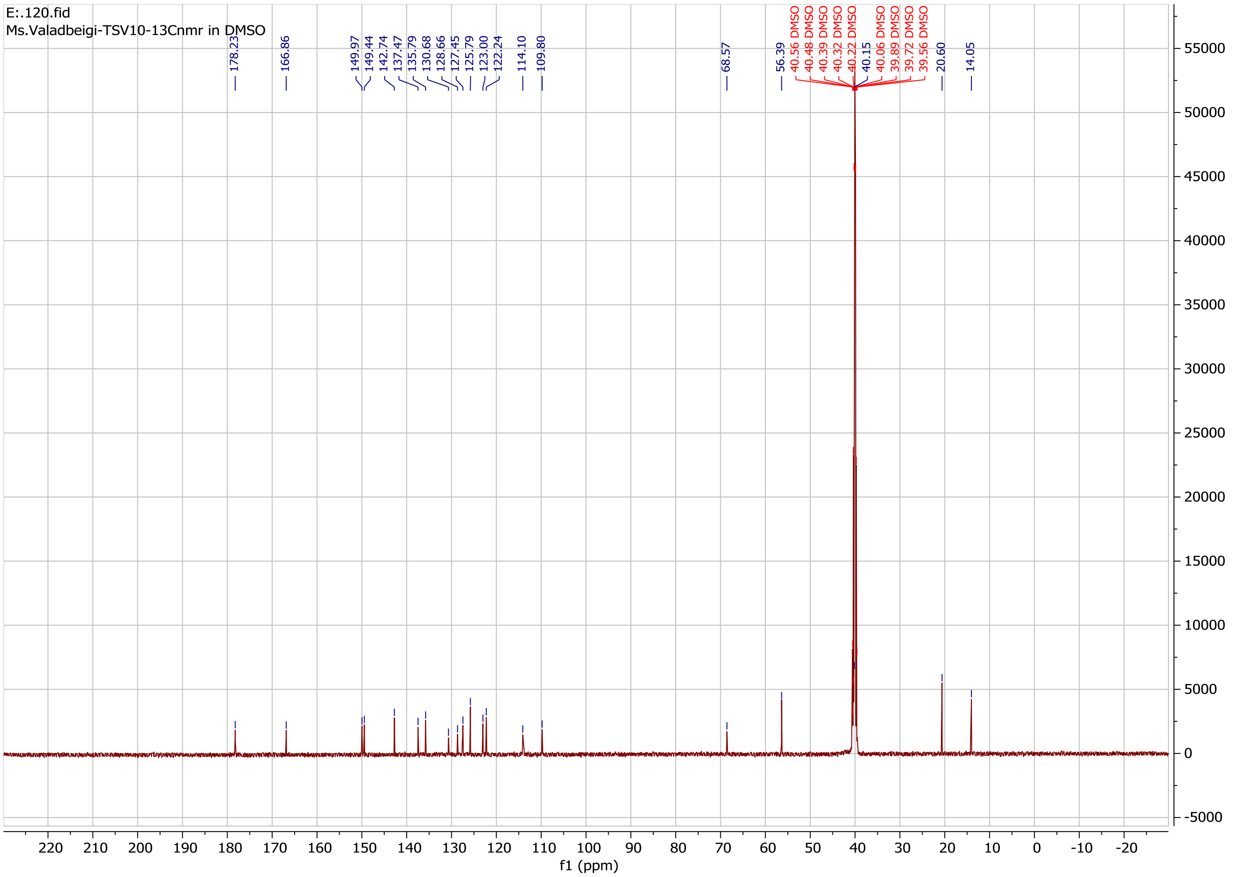

Mass spectra of 7j

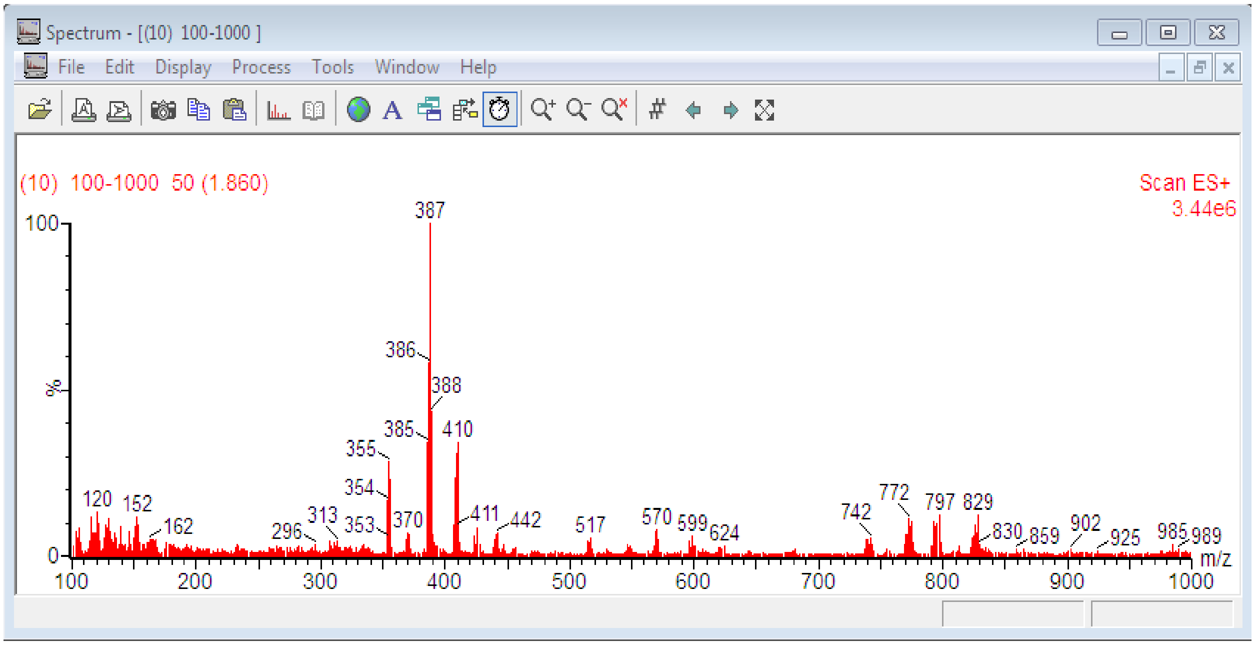

[1] HNMR

2-{4-[(Z)-[(carbamothioylamino)imino]methyl]-2-methoxyphenoxy}-N-(4- chlorophenyl) acetamide (7k)

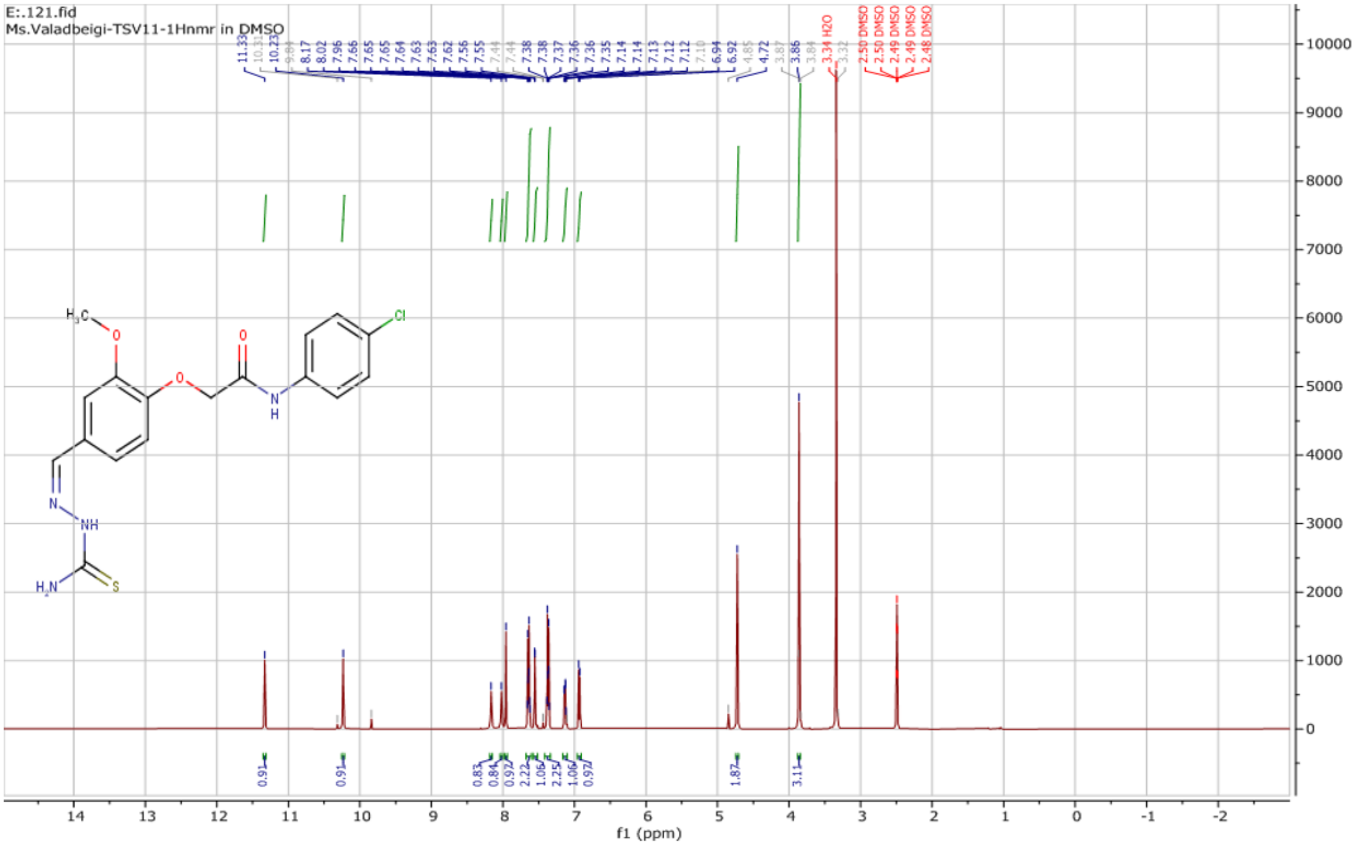

^{1}H NMR of 7k

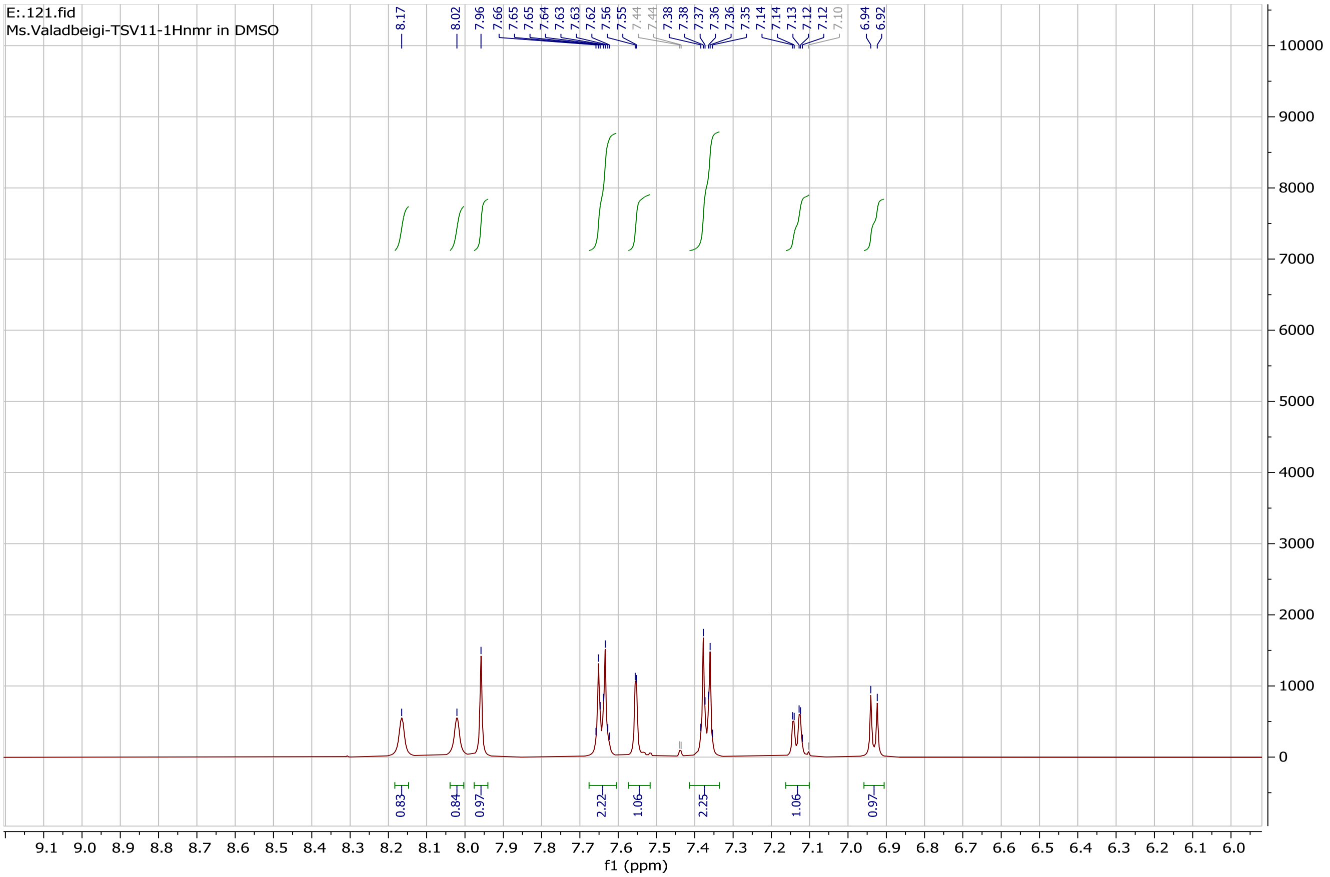
E:.121.fid
Ms.Valadbeigi-TSV11-1Hnmr in DMSO
8.17
8.02
7.96
7.66
7.65
7.65
7.64
7.63
7.63
7.62
7.56
7.55
7.44
7.44
7.38
7.37
7.36
7.36
7.35
7.14
7.14
7.13
7.12
7.12
7.10
6.94
6.92
0.83
0.84
0.97
2.22
1.06
2.25
1.06
0.97
f1 (ppm)

^{13}C NMR of 7k

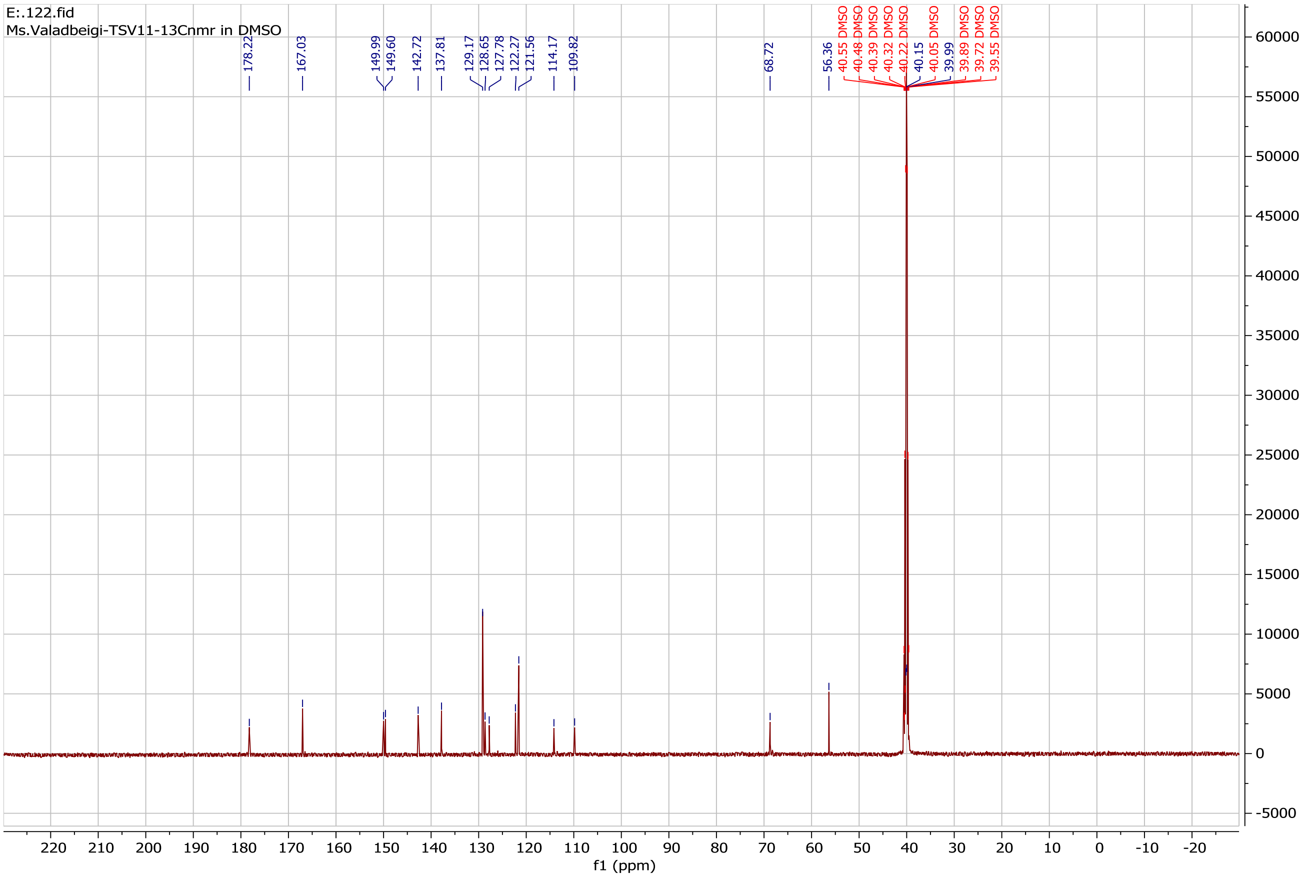

Mass spectra of 7k

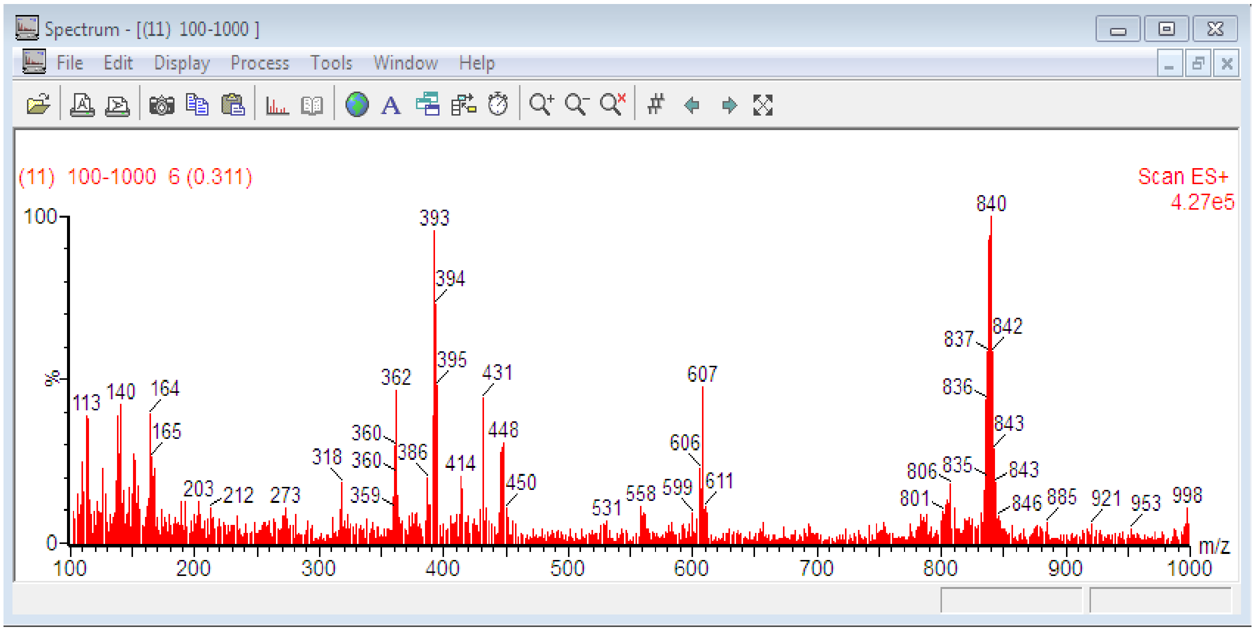

^{1}H NMR

2-{4-[(Z)-[(carbamothioylamino)imino]methyl]-2-methoxyphenoxy}-N-(2,4-dimethylphenyl)acetamide (7l)

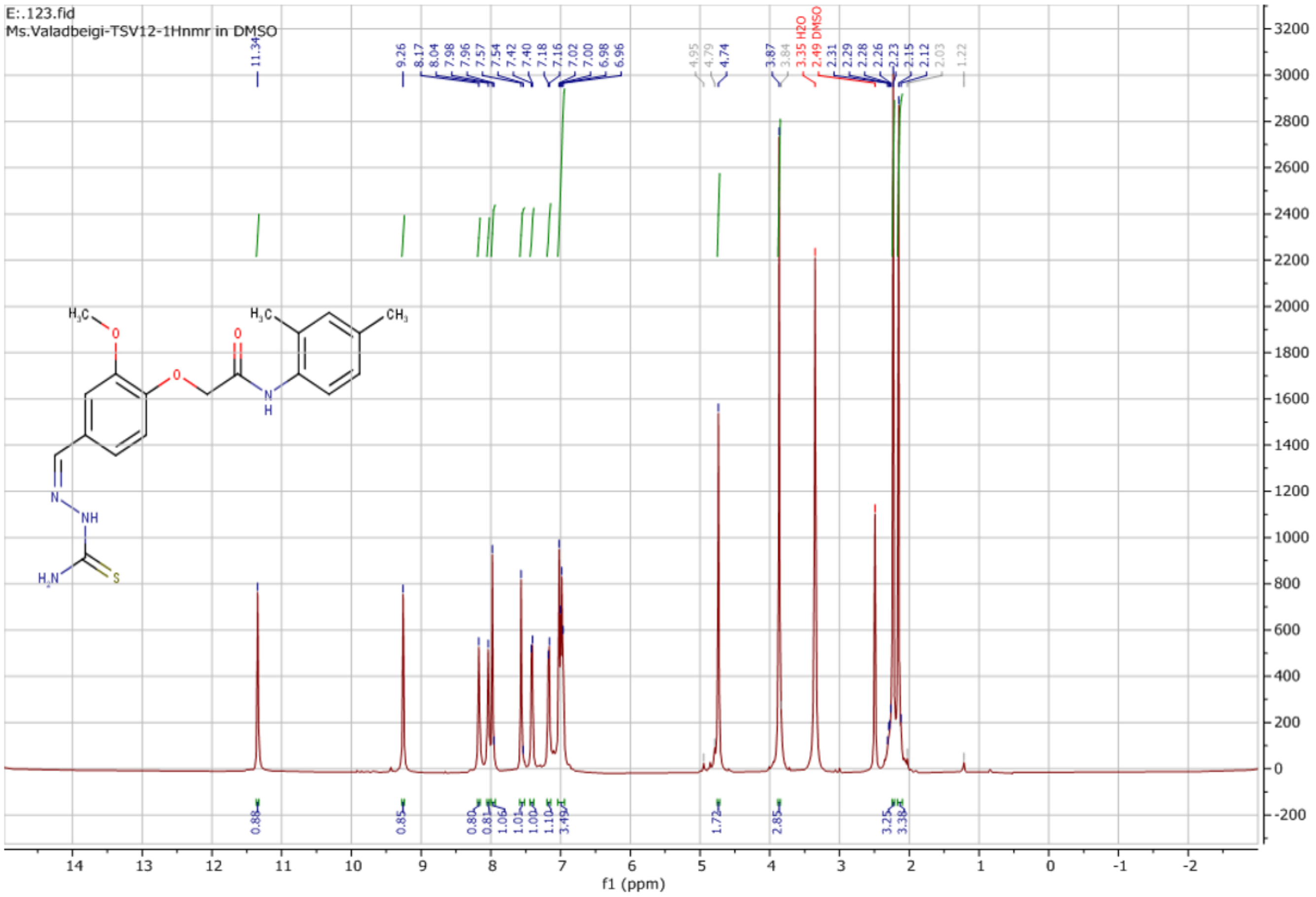

H[1] NMR of 7l

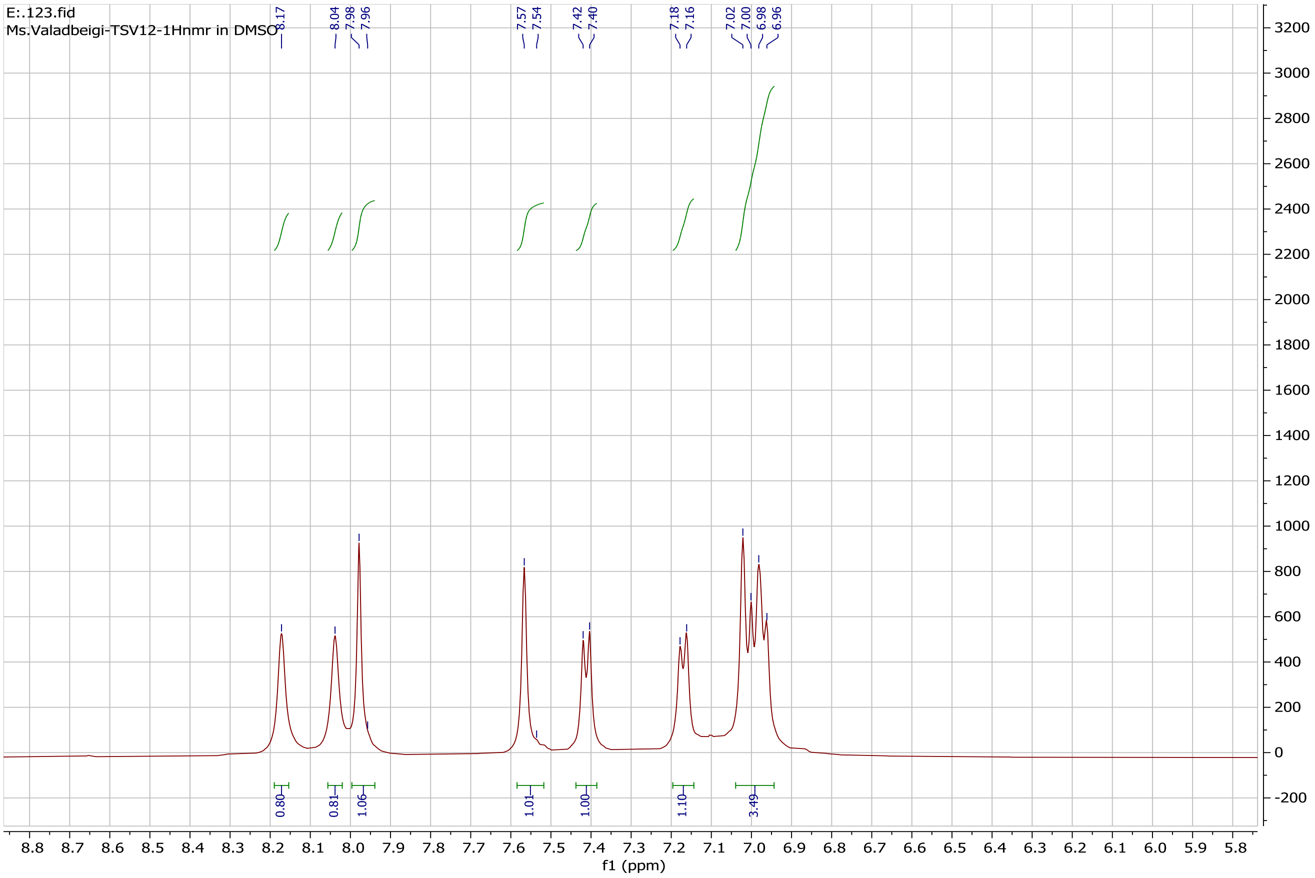
E:.123.fid
Ms.Valadbeigi-TSV12-1Hnmr in DMSO
8.17
8.04
7.98
7.96
7.57
7.54
7.42
7.40
7.18
7.16
7.02
7.00
6.98
6.96
0.80
0.81
1.06
1.01
1.00
1.10
3.49
f1 (ppm)

^{13}C NMR of 7l

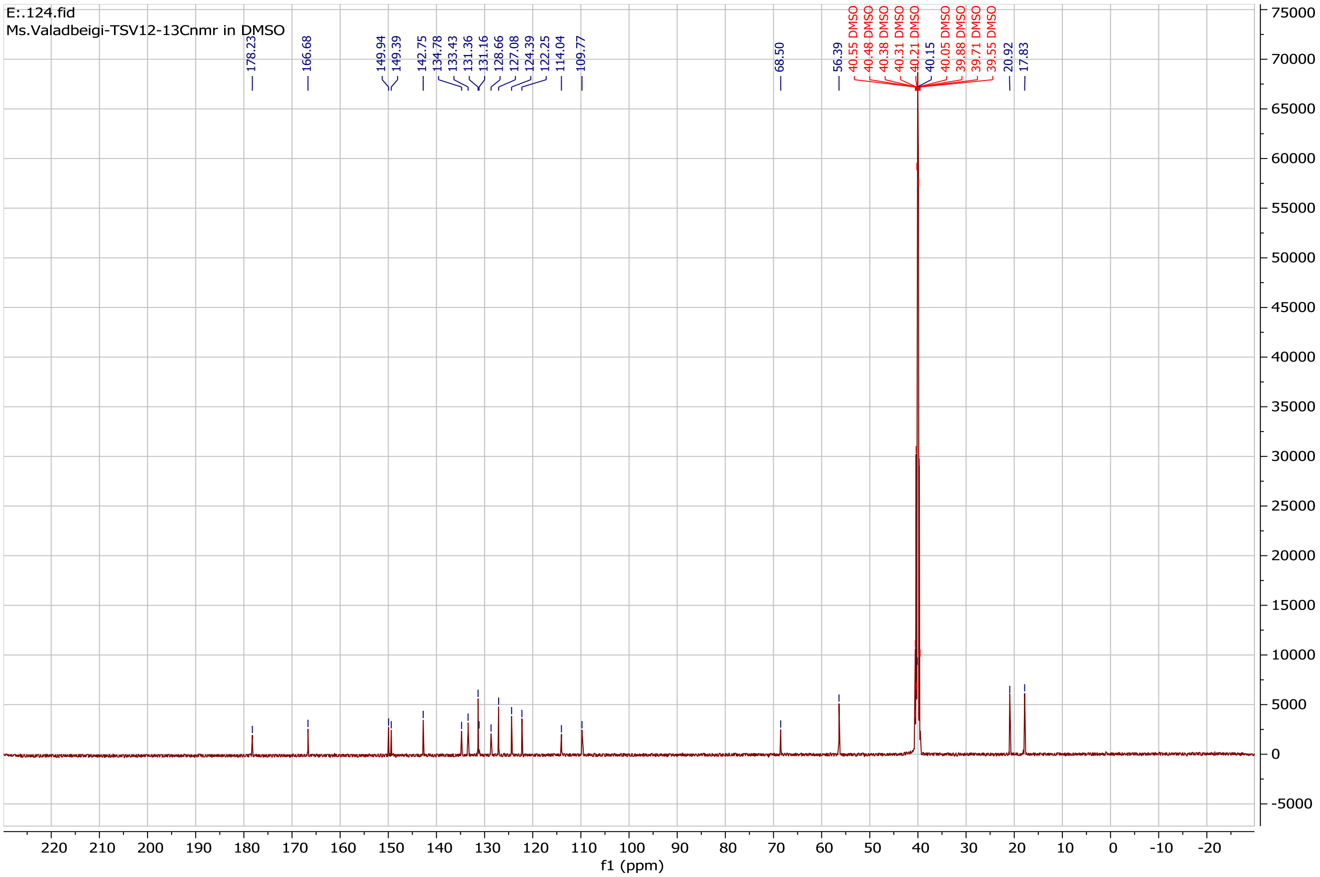

Mass spectra of 7l

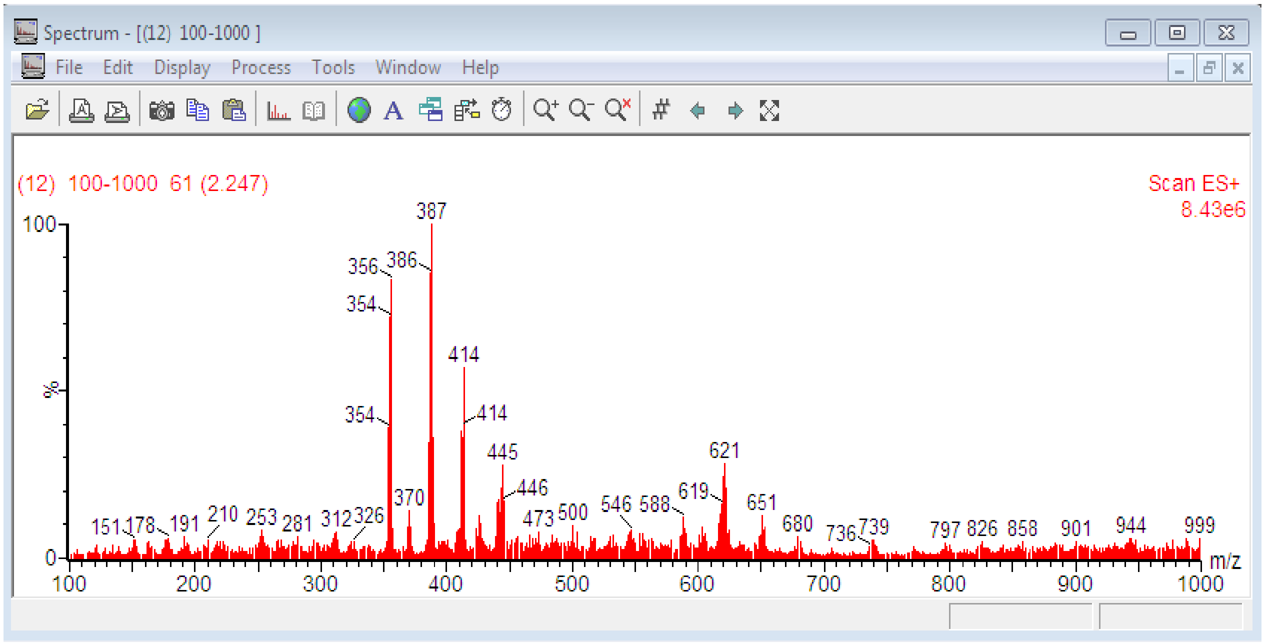

^{1}H NMR

2-{4-[(Z)-[(carbamothioylamino)imino]methyl]-2-methoxyphenoxy}-N-(4-methoxyphenyl) acetamide (7m)

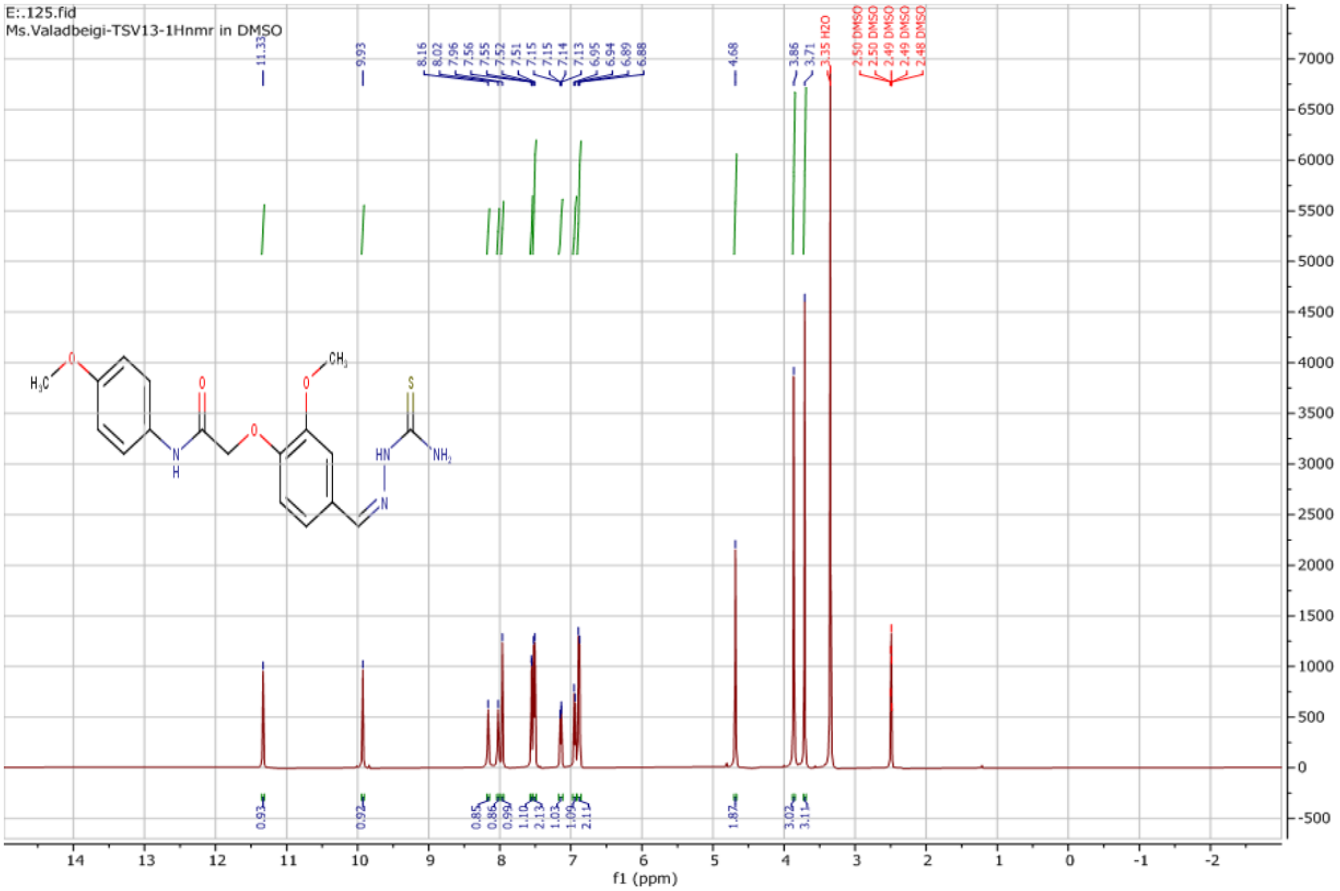

^{1}H NMR of 7m

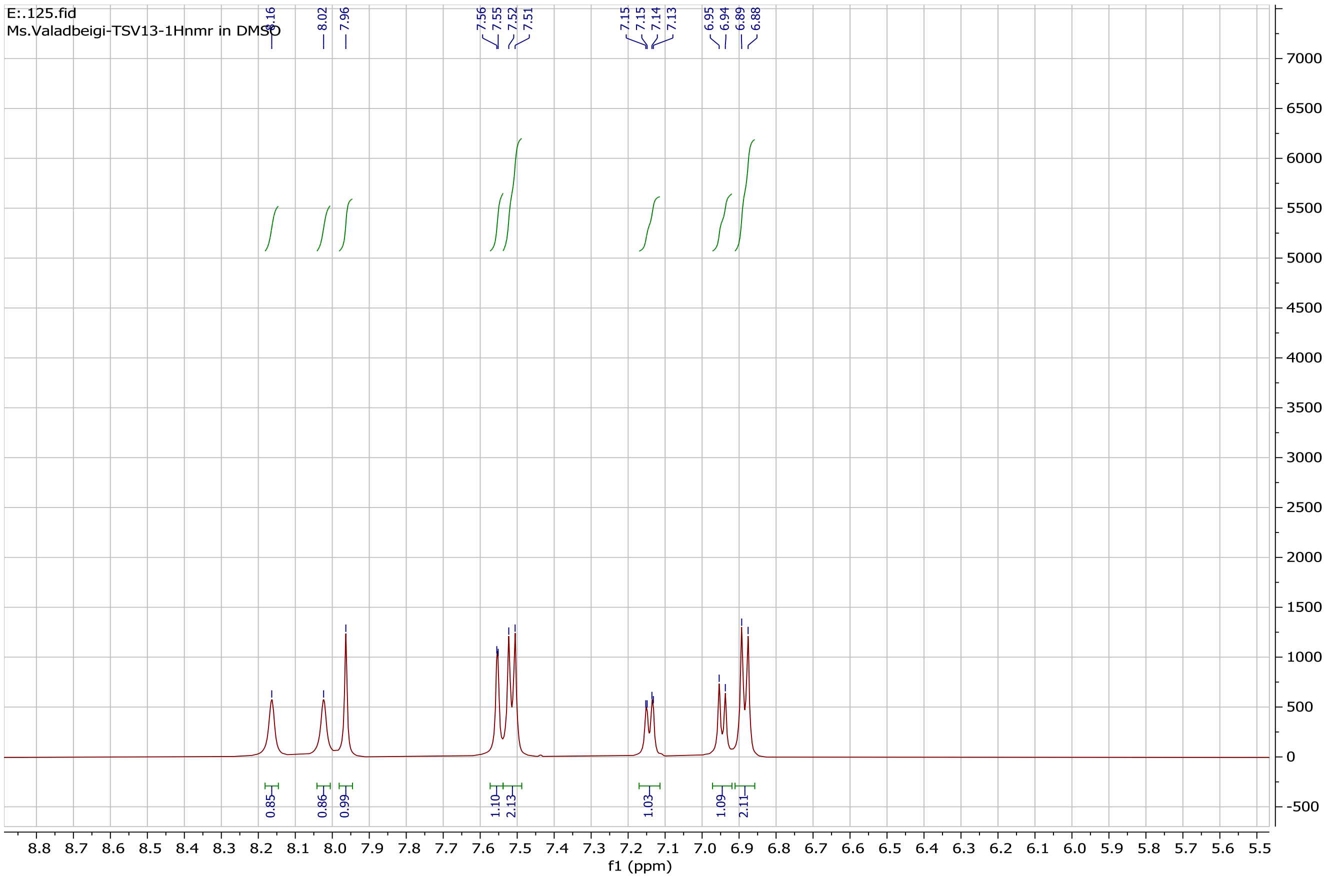

E:.125.fid
Ms.Valadbeigi-TSV13-1Hnmr in DMSO
8.16
8.02
7.96
7.56
7.55
7.52
7.51
7.15
7.15
7.14
7.13
6.95
6.94
6.89
6.88
0.85
0.86
0.99
1.10
2.13
1.03
1.09
2.11
f1 (ppm)

^{13}C NMR of 7m

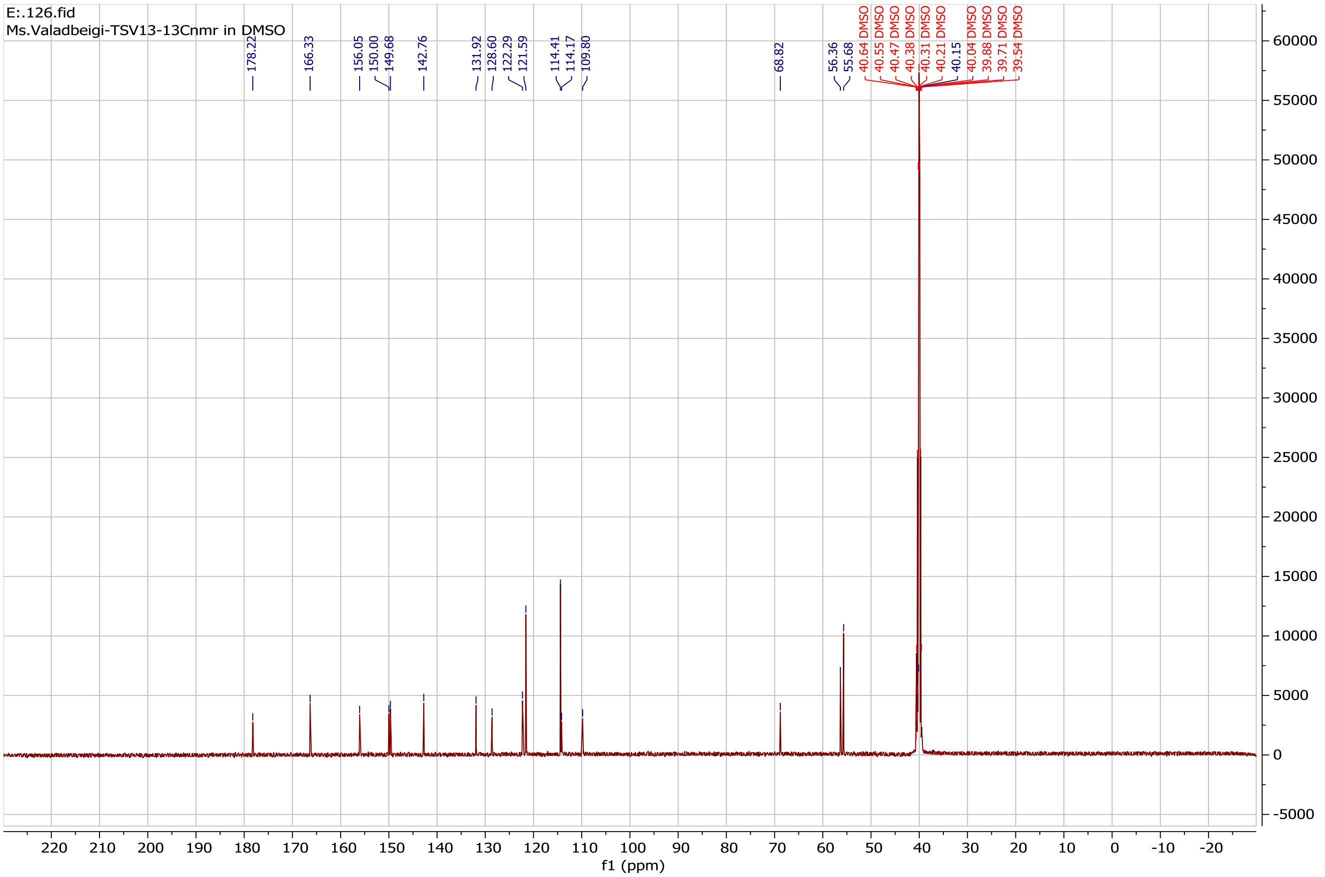

Mass spectra of 7m

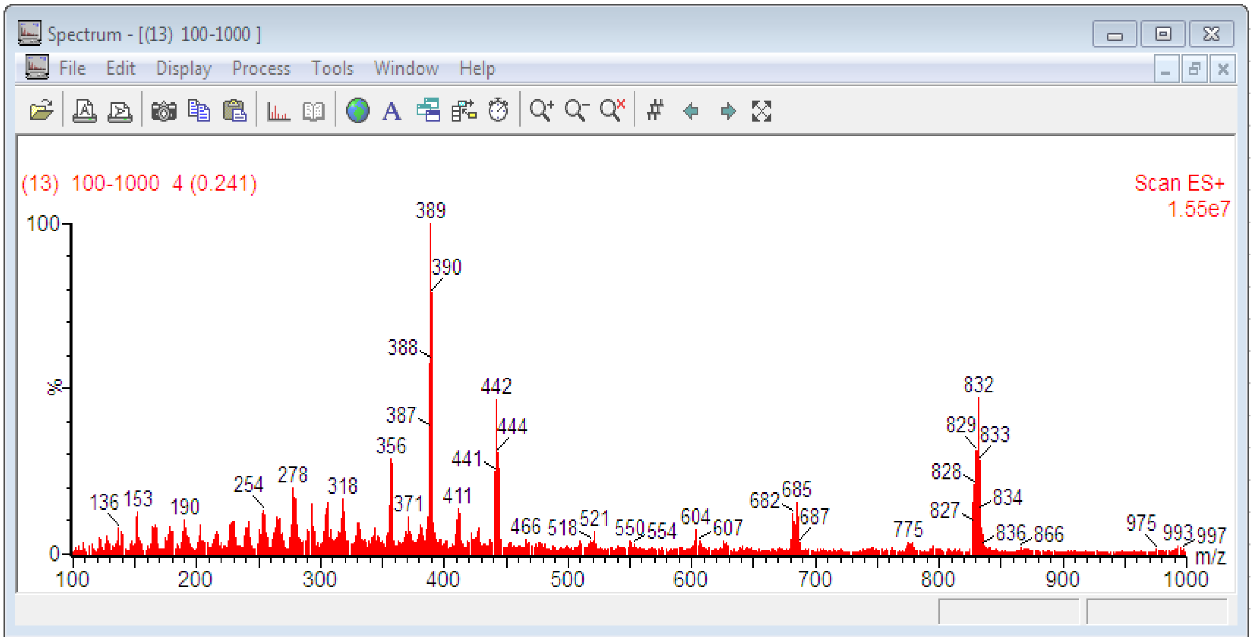

[1] HNMR

2-{4-[(Z)-[(carbamothioylamino)imino]methyl]-2-methoxyphenoxy}-N-(2-fluorophenyl) acetamide (7n)

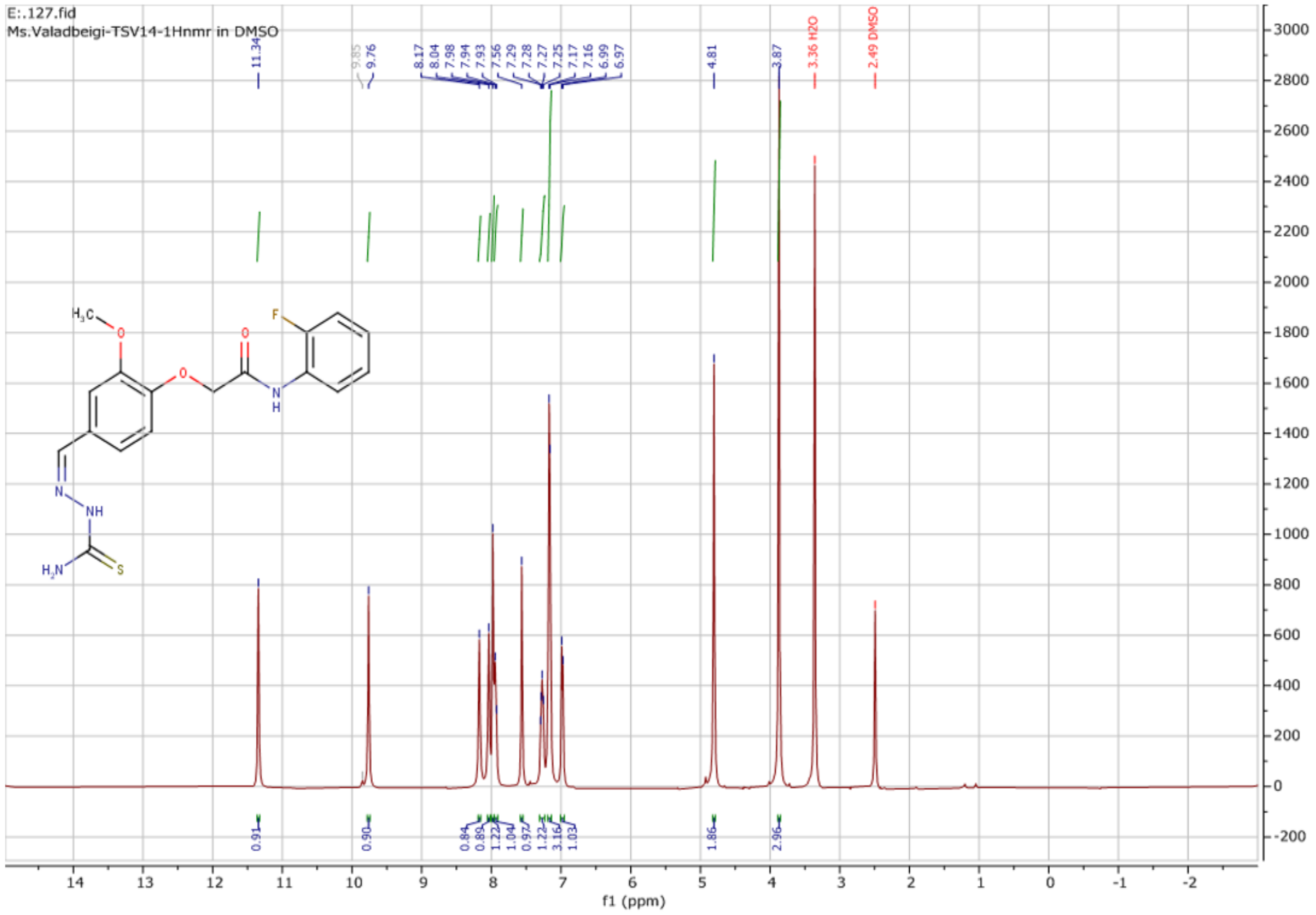

^{1}H NMR of 7n

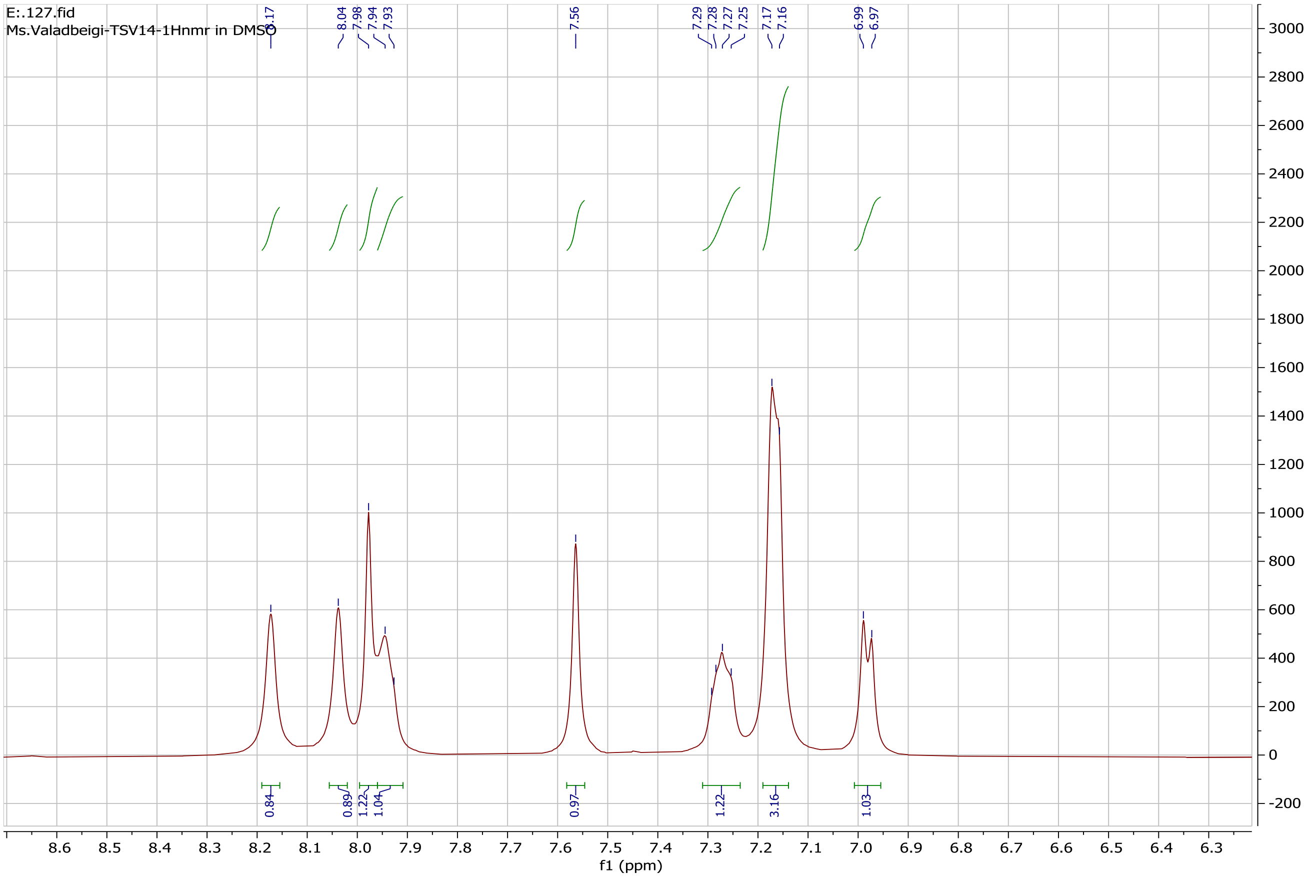
E:.127.fid
Ms.Valadbeigi-TSV14-1Hnmr in DMSO
8.17
8.04
7.98
7.94
7.93
7.56
7.29
7.28
7.27
7.25
7.17
7.16
6.99
6.97
0.84
0.89
1.22
1.04
0.97
1.22
3.16
1.03
f1 (ppm)

^{13}C NMR of 7n

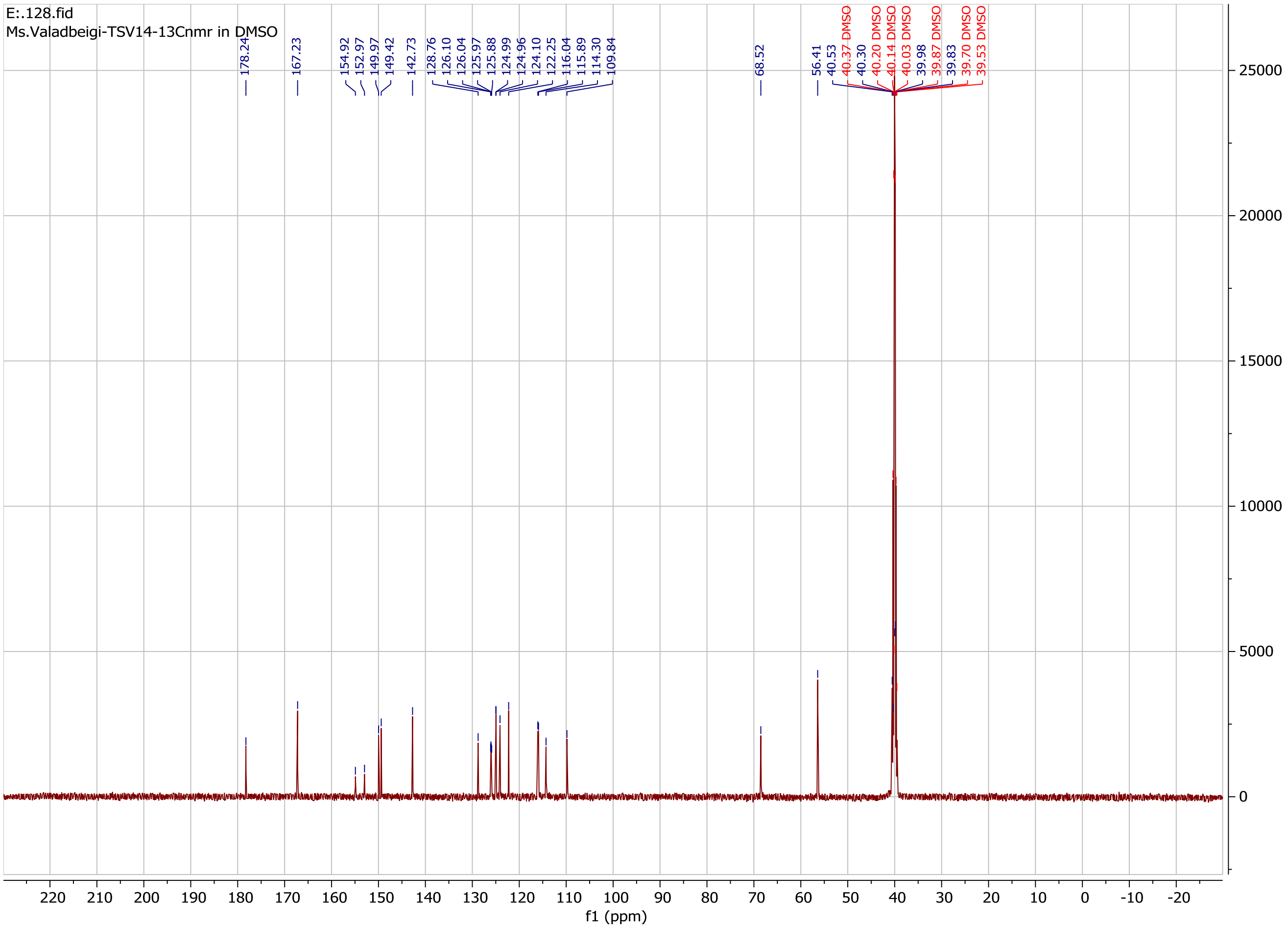

Mass spectra of 7n

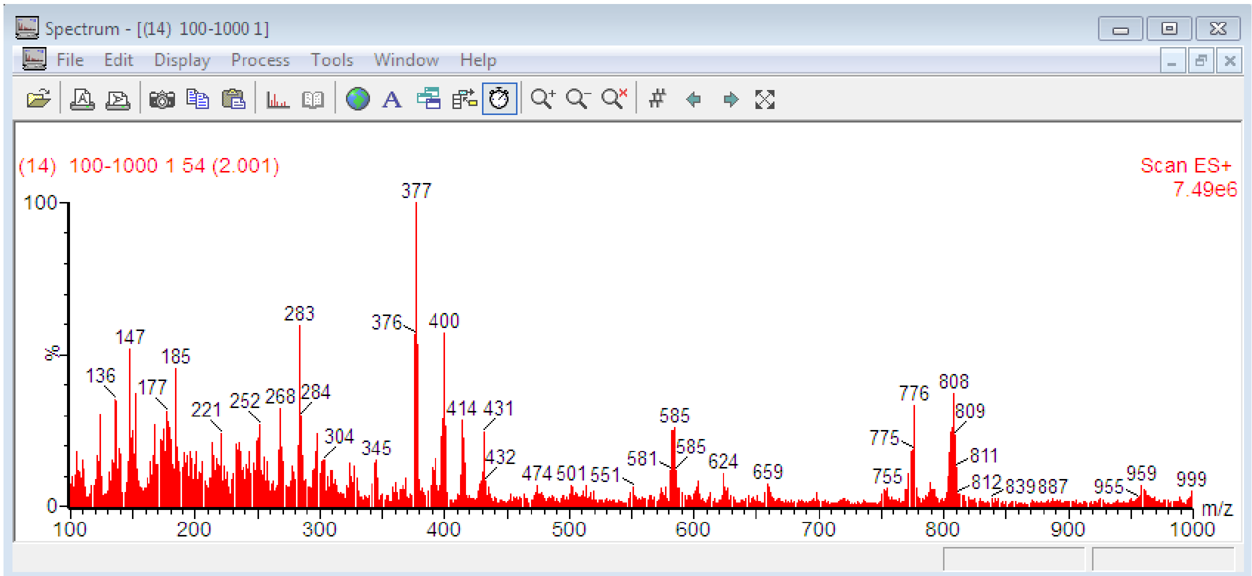

Supplement: supporting information file.pdf [file IENZ_A_2580515_SM1550.pdf]
